# The lipid bilayer strengthens the cooperative network of membrane proteins

**DOI:** 10.1126/sciadv.adv9568

**Published:** 2025-07-02

**Authors:** Shaima MuhammedNazaar, Jiaqi Yao, Matthew R. Necelis, Yein C. Park, Zhongtian Shen, Michael D. Bridges, Ruiqiong Guo, Nicole Swope, May S. Rhee, Miyeon Kim, Kelly H. Kim, Wayne L. Hubbell, Karen G. Fleming, Linda Columbus, Seung-gu Kang, Heedeok Hong

**Affiliations:** ^1^ Department of Chemistry, Michigan State University, East Lansing, MI 48824, USA.; ^2^ Department of Chemistry, University of Virginia, Charlottesville, VA 22904, USA.; ^3^ Thomas C. Jenkins Department of Biophysics, Johns Hopkins University, Baltimore, MD 21218, USA.; ^4^ National Institutes of Health–Johns Hopkins Graduate Partnership Program, Bethesda, MD 20892, USA.; ^5^ Jules Stein Eye Institute, Department of Chemistry and Biochemistry, University of California, Los Angeles, Los Angeles, CA 90095, USA.; ^6^ Department of Biochemistry & Molecular Biology, Michigan State University, East Lansing, MI 48824, USA.; ^7^ SKKU Advanced Institute of Nano Technology (SAINT), Department of Nano Science & Technology, and Department of Nano Engineering, Sungkyunkwan University, Suwon, Gyeonggi-do 16419, Republic of Korea.; ^8^ Computational Biology Center, IBM Thomas J. Watson Research Center, Yorktown Heights, NY 10598, USA.

## Abstract

Membrane proteins fold and function in a lipid bilayer constituting cell membranes. Nonetheless, their structure and function can be recapitulated in diverse amphiphilic assemblies whose compositions deviate from native membranes. It remains unclear how various hydrophobic environments stabilize membrane proteins and whether lipids play any unique role in protein stability compared to other types of amphiphiles. Here, using the evolutionarily unrelated α-helical and β-barrel membrane proteins from *Escherichia coli*, we find that the hydrophobic thickness and the strength of amphiphile-amphiphile packing in amphiphilic assemblies are critical determinants of protein stability. Lipid solvation enhances protein stability by facilitating residue burial in the protein interior, reminiscent of the lipophobic effect. This lipid-mediated mechanism also strengthens the cooperative residue-interaction network, promoting the propagation of local structural perturbations throughout the protein. This study demonstrates the pivotal role of lipid solvation in modulating the stability of membrane proteins and their responses to external stimuli.

## Teaser

Physical properties of lipid membranes are key determinants of the stability and cooperativity of membrane proteins.

## INTRODUCTION

The solvent environment plays a pivotal role in the folding and function of proteins (*1*, *2*). In the case of water-soluble proteins, the hydrophobic effect provides a critical driving force for folding by promoting the cohesion of nonpolar residues in the protein core and the expulsion of solvating water molecules into the bulk aqueous phase (*3*). Involving the collective formation and dismantling of structured hydrogen-bond (H-bond) networks, the solvent effect of water further mediates cooperativity in protein folding and allosteric protein-ligand interactions (*1*, *2*, *4*–*6*).

Unlike water-soluble proteins, membrane proteins fold and function in a lipid bilayer, which serves as a hydrophobic solvent-like environment in cell membranes. The folding of helical membrane proteins can be described using the two-stage model (*7*). In stage I, transmembrane (TM) helices are formed across the bilayer. This stage is primarily driven by the hydrophobic effect inducing the burial of nonpolar polypeptide segments into the nonpolar interior of the bilayer and by the favorable formation of backbone H-bonds therein (*8*, *9*). In stage II, the TM helices associate to form a compact native structure. Recent studies show that the denatured state ensemble (DSE) prior to compaction is highly dynamic and conformationally diverse: The TM helices can flip across the membrane, unfold at the water-membrane interface, or partially associate with one another (*10*–*14*). Because the hydrophobic effect is negligible within the bilayer due to the absence of water, this stage can be driven by various molecular forces, including interhelical van der Waals (vdW) packing and polar interactions (*15*–*18*), backbone and side-chain entropies (*19*, *20*), and selective lipid binding (*21*, *22*). Membrane properties, such as the lateral pressure profile and lipid packing density as well as membrane deformation caused by hydrophobic thickness mismatch between the protein and the bilayer, are known to influence the oligomerization of large membrane proteins and single-spanning TM helices (*23*–*29*). Nonetheless, it is not well understood how those effects involving the membrane affect the folding of multispanning membrane proteins (*30*–*32*).

The lipid composition of cell membranes is highly heterogeneous, varying across species, organelles, the inner and outer leaflets of the bilayer, and in response to environmental stresses (*33*–*35*). Although some studies highlight the importance of native lipid composition for the structure and function of membrane proteins (*22*, *33*, *34*, *36*), others point out that their fold and activity are tolerant to wide variations in lipid composition and can even be recapitulated in a broad range of artificial amphiphilic assemblies (e.g., micelles, bicelles, nanodiscs, liposomes, lipid cubic phases, amphipols, peptidiscs, and amphipathic protein scaffolds), whose chemical compositions vastly deviate from the native membranes (*36*–*46*). Still, there are multiple examples where the structure of a given membrane protein depends on the choice of an amphiphilic assembly (*47*).

The broad environmental sensitivity of membrane proteins raises questions about which properties of hydrophobic environments are critical for the proteins’ conformational stability and whether lipids contribute uniquely to the stability compared to other types of amphiphiles. Although recent studies have primarily focused on high-affinity, selectively bound lipids associated with membrane proteins (*21*, *22*), the role of low-affinity, “solvating lipids” in protein folding and function remains largely unexplored. Here, we investigate the properties of hydrophobic environments that influence stage II of membrane protein folding, as well as the effects of lipid solvation on protein stability and cooperativity. Cooperativity, which links the behaviors of distant sites (*48*), is thought to underlie the function of membrane proteins, including ion channels, receptors, transporters, and enzymes, by enabling the propagation of local physical or chemical stimuli across the structure. It remains elusive whether solvating lipids modulate this cooperativity as water does for water-soluble proteins (*1*, *4*, *6*).

Here, we hypothesized that membrane hydrophobic thickness serves as a minimal requirement for stabilizing the secondary (stage I) and tertiary (stage II) structures of membrane proteins and that additional membrane properties modulate the strength and cooperativity of tertiary interactions. To test this hypothesis, we used the monomeric six-helical bundle membrane protein GlpG from *Escherichia coli*, a member of the universally conserved rhomboid protease family, as a model (*49*). We conducted a comparative analysis of the stability, cooperative residue-interaction network, and solvation dynamics of GlpG in two distinct hydrophobic environments widely used in structural and functional studies of membrane proteins: bicelles, which are discoidal bilayer fragments edge-stabilized by detergents, and detergent micelles (*50*–*53*). To uncover membrane properties critical for protein stability and cooperativity, bicelles offer a valuable model system because their physical characteristics can be continuously tuned by varying the lipid content, ranging from micelles to detergent-rich bicelles and lipid-rich bicelles that closely mimic the bilayer structure in cell membranes (*54*–*57*). In addition, bicelles undergo rapid lipid exchange with one another (*58*), enabling dynamic adjustment of the number of solvating lipids during folding and denaturation transitions occurring within the bilayer. To further test whether the modulation of stability and cooperativity by membrane properties is a generalizable principle, we extended our analysis to an evolutionarily unrelated β barrel membrane protein, OmpLA from *E. coli*.

Our study reveals a pivotal role of lipid solvation in shaping the folding energy landscape and internal residue coupling of membrane proteins. We find that physical properties of amphiphilic assemblies, including hydrophobic thickness and the strength of amphiphile-amphiphile packing, are key determinants of membrane protein stability. Compared to micelles, lipid bilayers enhance protein stability by promoting residue burial within the protein interior. Unexpectedly, bilayers also facilitate the propagation of local structural perturbations throughout the protein by strengthening the cooperative network of residue interactions. Molecular dynamics (MD) simulations suggest that these lipid-induced effects stem from inefficient protein solvation by lipids, which favors intraprotein interactions over lipid-protein interactions. These findings provide an insight into how lipid bilayers mediate the stability and function of membrane proteins.

## RESULTS

### Steric trapping strategy to measure GlpG stability

**Fig. 1.**
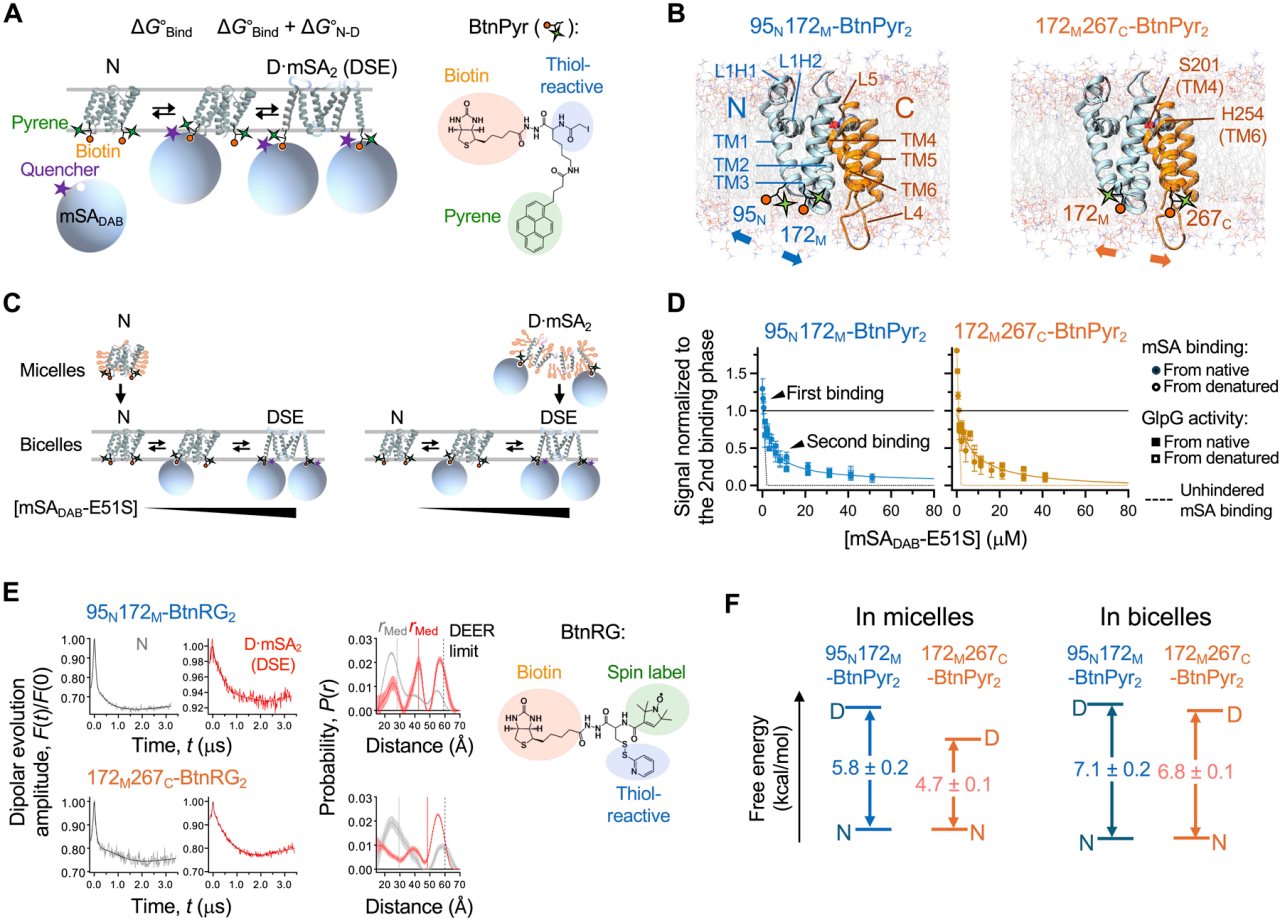
Thermodynamic stability of GlpG using steric trapping. (**A**) Steric trapping scheme to determine the thermodynamic stability ( $∆{G{}^{\circ}}_{N-D}$ ) of GlpG by measuring the binding of mSA to the biotin tags on GlpG. BtnPyr: a thiol-reactive biotin derivative with fluorescent pyrene; N: the native state; D⋅mSA_2_: the denatured state with two bound mSA molecules; DSE: the denatured state ensemble; mSA_DAB_: mSA labeled with the dabcyl quencher. (**B**) Structure of GlpG annotated with the secondary structural elements, N- and C-subdomains, the positions of the biotin pair, and the catalytic dyad for proteolysis (Ser^201^/His^254^). (**C**) Scheme for testing the reversibility of GlpG folding and mSA binding, and the coupling between them. (**D**) Binding isotherms between double-biotinylated variants of GlpG and mSA_DAB_-E51S. GlpG activity for the TM model substrate, LYTM2, with increasing concentrations of mSA_DAB_-E51S is overlaid. Errors denote ± SEM (*N* = 4 biological replicates). (**E**) DEER spectroscopy. Dipolar evolution data were fitted to yield the interspin distances. The error bar at each distance denotes ± SD of the fitted probability. “DEER limit”: the maximal nominal interspin distance detectible with confidence; “*r*_Med_”: the median interspin distance. (**F**) Subdomain stability of GlpG in DDM micelles (*60*) and DMPC:CHAPS bicelles.

Determining the thermodynamic stability of a helical membrane protein ( $∆{G{}^{\circ}}_{N-D}$ is the free energy change from the denatured to the native state in the membrane) is a daunting task due to the inherent difficulty of achieving reversible folding in a lipid bilayer environment (*32*). Here, we overcame this challenge using the steric trapping strategy, which exploits the coupling between the spontaneous denaturation of a doubly biotinylated protein to the simultaneous binding of two bulky monovalent streptavidin (mSA: a tetramer engineered to have only one active biotin-binding subunit; 52 kDa) molecules (Fig. 1A and fig. S1A) (*59*–*61*).

Briefly, GlpG is labeled with biotin tags at two specific residues, which are spatially close in the native tertiary structure but distant in the primary sequence. A first mSA binds unhindered to either biotin tag ( $∆{G{}^{\circ}}_{\text{Bind}}$ ). Because of steric hindrance from the first bound mSA, a second mSA can bind only when the tertiary contacts between the biotinylated residues are spontaneously disrupted. The binding affinity of mSA to the biotin tag can be tuned through amino acid substitutions in its active subunit (fig. S1B) (*59*–*61*). The coupling between GlpG denaturation and mSA binding leads to attenuated second binding ( $∆{G{}^{\circ}}_{\text{Bind}}+∆{G{}^{\circ}}_{N-D}$ ). By quantifying the degree of attenuation of the second mSA binding event, the $∆{G{}^{\circ}}_{N-D}$ of GlpG can be determined. This strategy allows thermodynamic analysis of stage II of membrane protein folding under native conditions.

GlpG is a two-domain protein consisting of the divergent N-terminal cytosolic domain (residues 1 to 74) and the conserved C-terminal catalytic TM domain (residues 87 to 276). It has been shown that the N-terminal domain facilitates diffusion of the protein in the membrane and may modulate GlpG’s proteolytic activity (*62*, *63*). Here, we focus on the stability of the isolated catalytic TM domain excluding potential interference from the extramembranous N-terminal domain.

### Stability enhancement of GlpG in lipid-enriched bicelles

The primary amphiphilic assemblies used for stability comparison were neutral bicelles composed of zwitterionic DMPC (1,2-dimyristoyl-*sn*-glycero-3-phosphocholine) and CHAPS {3-[(3-cholamidopropyl)dimethylammonio]-1-propane sulfonate} at a lipid-to-detergent molar ratio (*q* = [DMPC]/[CHAPS]) of 1.5 and neutral micelles formed by *n*-dodecyl-β-d-maltoside (DDM). Cryo–electron microscopy (cryo-EM) of protein-free bicelles revealed uniform discoidal particles with an average diameter (<*d*>_bicelles_) of ~90 Å, indicative of a lipid-enriched bilayer structure (fig. S2). In contrast, DDM micelles adopt an oblate-spheroidal shape, appearing more globular and smaller than bicelles, with <*d*>_micelles_ = ~60 Å (fig. S2) (*64*).

The steric trapping strategy has been established for measuring GlpG stability in DDM micelles (*60*). Here, we validated the strategy in bicelles (Fig. 1). Two double-cysteine variants of GlpG were labeled with BtnPyr, the thiol-reactive biotin derivative with fluorescent pyrene (Fig. 1A), resulting in the double-biotinylated constructs, 95_N_172_M_-BtnPyr_2_ and 172_M_267_C_-BtnPyr_2_ (*60*) (Fig. 1B). In these variants, 95, 172, and 267 indicate the residue positions of engineered cysteine, located on the N-terminal (N), middle (M), and C-terminal (C) helices, respectively. GlpG stability was thus assessed in the N- or C-terminal half (referred to as N- and C-subdomains), depending on the position of the biotin pair. Stability measurements were performed at room temperature (24.5° ± 0.5°C) under conditions where the bilayer region of lipid-enriched bicelles is expected to be predominantly fluid, although both gel and fluid phases likely coexist (see the section ‘Critical influences of physical proerties of amphiphilic assemblies on GlpG stability’ for bicelle phase transition data).

**Fig. 2.**
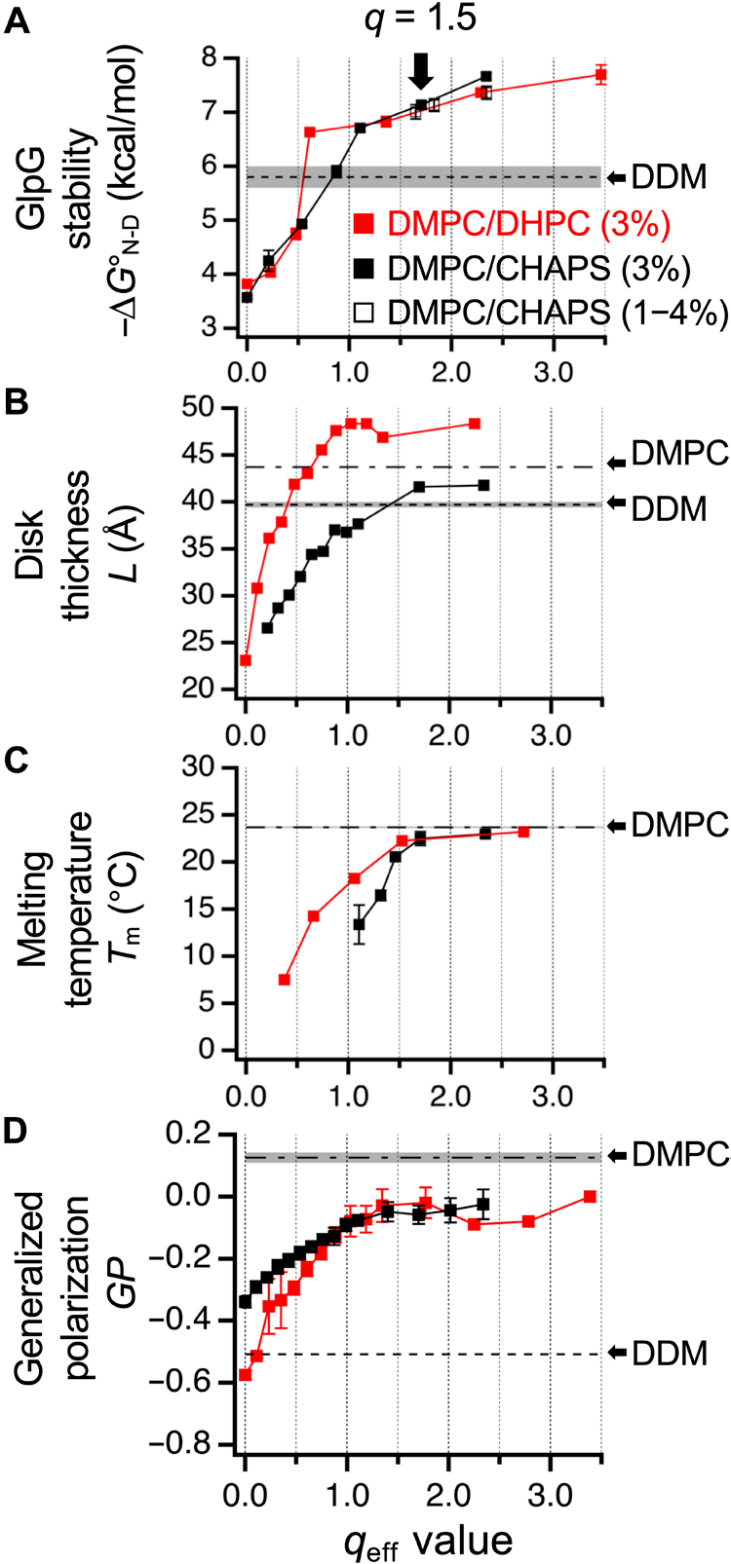
Relationship between GlpG stability and physical properties of bicelles measured as a function of lipid content. Lipid content, *q*_eff_, in DMPC:CHAPS or DMPC:DHPC bicelles was calculated using the fixed critical bicelle concentrations (Eq. 8 in Materials and Methods). (**A**) Stability (−$∆{G{}^{\circ}}_{N-D}$ ) of the double-biotinylated variant of GlpG, 95_N_172_M_-BtnPyr_2_. The *q*_eff_ was varied either by fixing the total amphiphile (i.e., lipids and detergents) concentration at 3% (w/v) or by diluting the bicelle solution at *q* = 1.5 (DMPC:CHAPS) to various final total amphiphile concentrations from 1 to 4% (w/v). Errors denote means ± SD from fitting. (**B**) Bicelle thickness (*L*) measured by SAXS. Thickness of DDM micelles was adapted from ref. (*64*). (**C**) Gel-fluid phase transition temperature (*T*_m_) measured by fluorescence anisotropy of diphenylhexatriene (DPH) incorporated into bicelles. The data for DHPC:DMPC bicelles were adapted from ref. (*54*). Each temperature-dependent anisotropy data was fitted to a sigmoid function, yielding *T*_m_ representing the inflection point during phase transition. Errors denote ± SEM (*N* = 3 or 4 biological replicates). (**D**) *GP* of Laurdan incorporated into bicelles. Errors denote ± SEM (*N* = 3 biological replicates).

To construct the binding isotherm between the double-biotinylated GlpG and mSA, either native or sterically denatured GlpG prepared in DDM micelles was transferred to bicelles by dilution in the presence of increasing concentrations of mSA_DAB_-E51S (an mSA variant labeled with a dabcyl quencher and engineered for reduced biotin affinity compared to mSA-WT) (Fig. 1C and figs. S3 and S4). Transfer efficiency ranged from 94 to 99% with a final [DMPC]/[DDM] ratio between 140 and 280 (fig. S3). The resulting binding isotherm, monitored by quenching of pyrene fluorescence from BtnPyr conjugated to GlpG (Fig. 1, A and D), exhibits tight unhindered first binding of mSA_DAB_-E51S followed by optimally attenuated second binding. The second binding phases obtained with initially folded and denatured GlpG agreed with each other. In parallel, the proteolytic activity of GlpG was measured as an indicator of folding. Activity decreased with increasing concentrations of mSA_DAB_-E51S, and this inactivation phase was consistent with the second binding phase (Fig. 1D and fig. S5). These results confirmed that GlpG denaturation is coupled to the second mSA binding and is reversible, thereby validating the steric trapping strategy.

We further characterized the conformational properties of the denature states generated via steric trapping. The doubly biotinylated variants of GlpG bound by two mSA molecules were more susceptible to proteolysis by Proteinase K compared to unbound GlpG, indicating increased conformational flexibility and water accessibility upon denaturation (fig. S6). To assess compactness of the denatured states, we used a spin-labeled, thiol-reactive biotin derivative (BtnRG) (Fig. 1E) (*60*). When doubly conjugated to GlpG, BtnRG enables both trapping of the denatured state via mSA binding and measurement of interspin distances using double electron-electron resonance spectroscopy (DEER) (*10*, *60*). Upon simultaneous mSA binding, the median interspin distance between the two BtnRG labels increased from 28 to 42 Å for 95_N_172_M_-BtnRG_2_ and from 29 to 48 Å for 172_M_267_C_-BtnRG_2_ (Fig. 1E and table S1). These results indicate that the TM helices are substantially separated in the denatured state of GlpG.

Last, fitting the attenuated second binding phases yielded $∆{G{}^{\circ}}_{N-D,{\text{bicelle}}^{N}}=-7.1\pm 0.2$ kcal/mol for N-subdomain and $∆{G{}^{\circ}}_{N-D,{\text{bicelle}}^{C}}=-6.8\pm 0.1$ kcal/mol for C-subdomain in bicelles (Fig. 1, D and F, and fig. S7). In contrast, in DDM micelles, the two subdomains exhibit distinct folding behaviors (Fig. 1F, left) (*60*). That is, N-subdomain ( $∆{G{}^{\circ}}_{N-D,{\text{micelle}}^{N}}=-5.8\pm 0.2$ kcal/mol), whose disruption induces global denaturation, is more stable than C-subdomain ( $∆{G{}^{\circ}}_{N-D,{\text{micelle}}^{C}}=-4.7\pm 0.1$ kcal/mol), which undergoes subglobal denaturation (*60*). Resultantly, compared to micelles, the bilayer environment of bicelles enhanced GlpG stability in two notable ways: Both subdomains were significantly stabilized by −1.3 ± 0.3 kcal/mol (N-subdomain) and −2.1 ± 0.3 kcal/mol (C-subdomain), and their stabilities became nearly uniform  $\begin{array}{c} \left(\left|∆{G{}^{\circ}}_{N-D,{\text{bicelle}}^{N}}-∆{G{}^{\circ}}_{N-D,{\text{bicelle}}^{C}}\right|=0.3\pm 0.3\;\text{kcal}/\text{mol}\right) \end{array}$ (Fig. 1F, right). The stability directly measured under native conditions (~−12⋅*k*_B_*T*) is larger than the stability extrapolated to zero force (~−6.5⋅*k*_B_*T*) in a molecular tweezer study (*65*–*67*), likely reflecting differences in the conformational properties and the degrees of freedom of the denatured states in the two experimental systems (fig. S8).

### Critical influences of physical properties of amphiphilic assemblies on GlpG stability

Next, we sought to identify the molecular basis for GlpG stabilization in the bilayer environment. As the lipid content (*q* value) increases in bicelles, lipid molecules preferentially segregate at the center to form a discoidal bilayer, whereas detergent molecules are excluded toward the periphery to stabilize the bilayer edges (*54*, *68*). However, lipid-detergent segregation is incomplete, and some detergent molecules remain incorporated in the bilayer, with the extent of partitioning depending on the lipid content (*54*). Thus, by tuning the *q* value, the physical properties of bicelles can be systematically controlled (*54*–*57*, *69*, *70*), providing a valuable opportunity to dissect the environmental factors that contribute to membrane protein stabilization.

To this end, we assessed GlpG stability in two types of bicelles, DMPC:CHAPS and DMPC:DHPC (1,2-dihexanoyl-*sn*-glycero-3-phosphocholine), as a function of lipid content in bicelles (*q*_eff_ is an effective *q* value) (Fig. 2A, fig. S9, and Materials and Methods) (*68*). We then compared changes in GlpG stability with alterations in key physical properties of the bicelles, including disk thickness [*L* is defined as the average headgroup-to-headgroup distance along the short axis of an oblate-ellipsoidal model determined by small-angle x-ray scattering (SAXS)] (*54*, *71*), the degree of lipid segregation (*T*_m_ is the gel-fluid phase transition temperature of the bilayer region) (*54*), and the strength of amphiphile-amphiphile packing [*GP* (generalized polarization) derived from Laurdan fluorescence] (*72*, *73*) (Fig. 2, B to D, and fig. S10, A to C). Notably, *T*_m_ and *GP* are related parameters: *T*_m_ reflects lipid-lipid packing, whereas *GP* captures the overall packing strength among all amphiphiles in the bicelles. These parameters were measured in the absence of GlpG to assess the effects of individual intrinsic bicelle properties on protein stability. We also note that GlpG has a narrower hydrophobic span (27 to 29 Å) than typical cellular membranes (30 to 40 Å), resulting in local membrane thinning (*62*, *74*, *75*). This thinning is known to influence the proteolytic activity and diffusion rate of GlpG in the membranes (*62*, *74*, *75*). Both fluid DMPC bilayers and DDM micelles provide hydrophobic thicknesses (24 to 28 Å for DMPC and 28 to 32 Å for DDM) that closely match GlpG’s hydrophobic belt (fig. S10D) (*64*, *71*, *75*–*77*).

GlpG exhibited significantly lower stability in CHAPS and DHPC micelles ( $-\;∆{G{}^{\circ}}_{N-D^{N}}=$ ~3.5 kcal/mol at *q*_eff_ = 0) compared to DDM micelles ( $-\;∆{G{}^{\circ}}_{N-D^{N}}=5.8$ kcal/mol) (Fig. 2A). As the lipid content increased, stability rose steeply in both bicelle systems, surpassing the level in DDM micelles until *q*_eff_ reached 0.6 in DMPC:DHPC and 1.1 in DMPC:CHAPS. With further increases in *q*_eff_, the stabilities in the two bicelle systems converged and continued to rise gradually, reaching ~8 kcal/mol, which is the upper limit detectable with the current steric trapping toolkit. This steady increase in stability was driven solely by lipid content, independent of the specific detergent used.

Similar to stability, all three parameters (*L*, *T*_m_, and *GP*) exhibited steep increases as *q*_eff_ increased in the low *q*_eff_ regimes (≤0.6 in DMPC:DHPC and ≤1.1 in DMPC:CHAPS) (Fig. 2, B to D). These increases were more pronounced in DMPC:DHPC, indicating a higher sensitivity to lipid content and more efficient lipid segregation compared to DMPC:CHAPS. Consistent with this, a cooperative gel-to-fluid transition emerged in DMPC:DHPC at *q*_eff_ = 0.3 (Fig. 2C and fig. S10B) (*54*), whereas a comparable transition was not observed in DMPC:CHAPS until *q*_eff_ = 1.1. At the higher *q*_eff_ regimes, each parameter gradually approached its respective value in pure DMPC liposomes in both bicelle systems. Notably, the difference in the near-saturation point of *GP* was obscure between the two bicelle systems (Fig. 2D), likely reflecting the partitioning of Laurdan (a lauric acid chain-conjugated naphthalene derivative) into both lipid-rich and detergent-rich domains within the bicelles.

Resultantly, in both bicelle types, GlpG stability showed a strong correlation with all three structural parameters (*L*, *T*_m_, and *GP*) as lipid contents varied (Fig. 2 and fig. S11). Because of the strong correlation, it was challenging to pinpoint which parameter most directly influences GlpG stability. However, we noticed that DDM micelles, which closely match GlpG’s hydrophobic thickness (fig. S10D), also provided substantial stabilization ( $-\;∆{G{}^{\circ}}_{N-D^{N}}=5.8$ kcal/mol), surpassing the stabilities observed in low *q*_eff_ bicelles (Fig. 2, A and B). This suggests a positive relationship between protein stability and the degree of thickness matching. In contrast, although stabilizing GlpG, DDM micelles exhibited weak amphiphile-amphiphile packing (*GP* = −0.5) comparable to low *q*_eff_ bicelles (*GP* = −0.6 to −0.3) (Fig. 2D), indicating a weaker correlation between protein stability and the strength of amphiphile packing. Collectively, these findings highlight that improved thickness matching is likely the primary driver of the steep stability increase observed in the low *q*_eff_ range.

In both bicelle systems, the transition from a steep to a more gradual increase in GlpG stability occurred at *q*_eff_ values where *T*_m_ exceeded ~10°C (~0.6 for DMPC:DHPC and ~1.1 for DMPC:CHAPS) (Fig. 2, A and C). This threshold suggests that lipid segregation and the resultant formation of a bilayer in bicelles are critical determinants of GlpG stability. Although disk thickness (*L*) was saturated near these *q*_eff_ values (Fig. 2B), continued, modest increases in lipid segregation and amphiphile packing (~+0.8°C/*q*_eff_ for *T*_m_ and ~+0.02/*q*_eff_ for *GP*; Fig. 2, C and D) contributed meaningfully to stabilization, accounting for ~25% of the overall change in protein stability.

Together, these findings demonstrate the profound influence of the physical properties of the hydrophobic environment on membrane protein stability. Among these, hydrophobic thickness matching between the amphiphilic assembly and the native protein emerges as a major determinant of stability. Notably, the strength of amphiphile-amphiphile (or lipid-lipid) packing, which does not directly involve an interaction with the protein, provides an additional, distinct layer of stability modulation. The specific chemical identity of the constituent detergents, however, appears to play a less important role in this modulation. The physical characteristics of our primary bicelle system (DMPC:CHAPS at *q* = 1.5) closely resemble those of DMPC liposomes (Fig. 2, B to D). Moreover, GlpG exhibits higher proteolytic activity in lipid-rich DMPC:CHAPS bicelles than in DDM micelles or DMPC:DHPC bicelles, reaching activity levels comparable to those observed in DMPC liposomes (fig. S12). Thus, although not a perfect mimic, our primary bicelle system provides a reasonable approximation of the lipid solvation environment found in the fluid DMPC bilayers.

### Facilitation of residue burial into the protein interior by lipid solvation

**Fig. 3.**
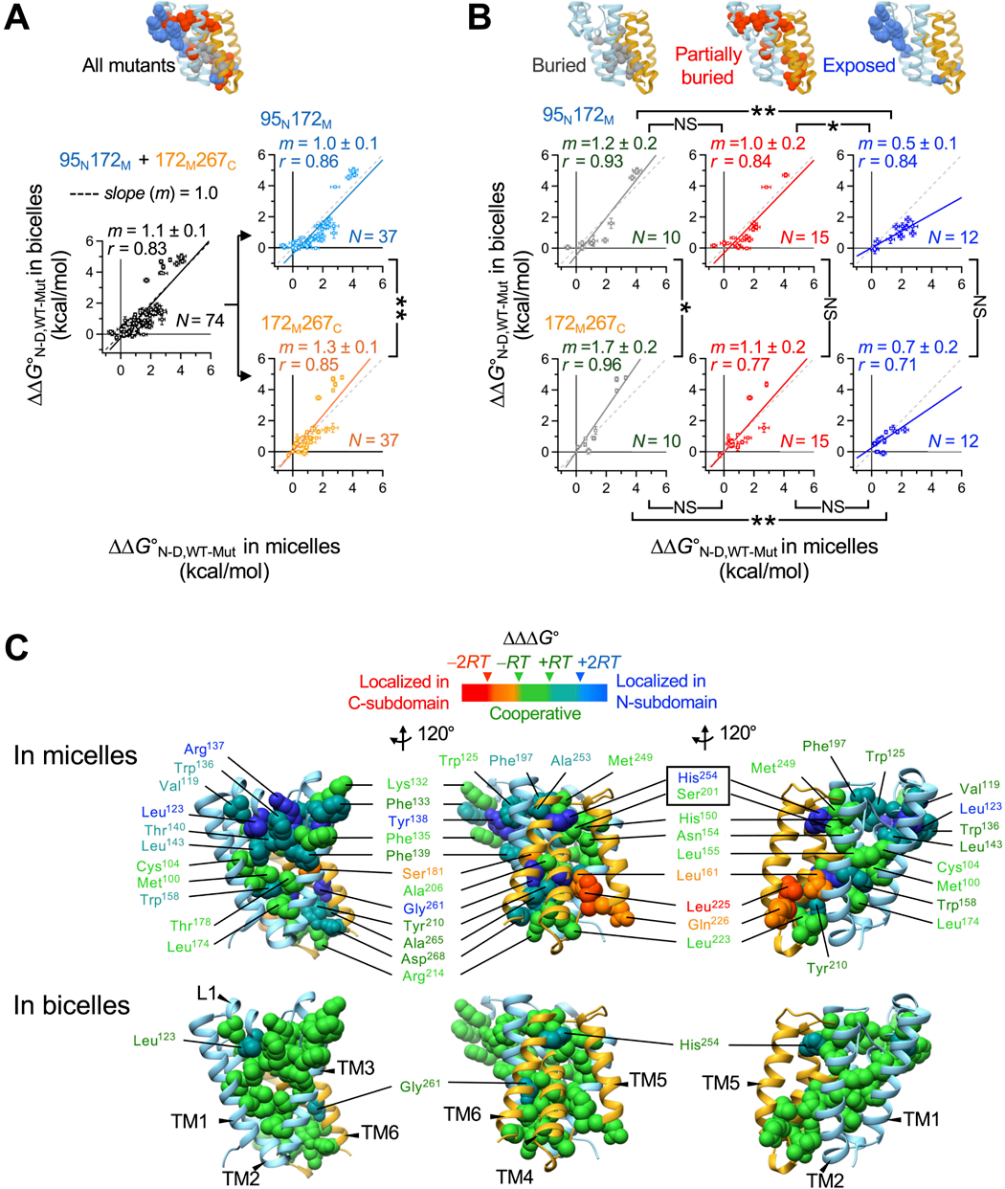
Mutation-induced stability changes ( $∆\;∆{G{}^{\circ}}_{N-D,\text{WT}-\text{Mut}}$ values) and cooperativity profiling of GlpG in micelles versus bicelles. (**A**) (Left) All $∆\;∆G_{N-D,\text{WT}-\text{Mut}}^{o}$ values measured at N-subdomain (95_N_172_M_) and C-subdomain (172_M_267_C_) in micelles versus bicelles. (Right) $∆\;∆{G{}^{\circ}}_{N-D,\text{WT}-\text{Mut}}$ values depending on the location of the biotin pair in micelles versus bicelles. (**B**) $∆\;∆{G{}^{\circ}}_{N-D,\text{WT}-\text{Mut}}$ values depending on the location of the biotin pair and on the degree of burial of mutated residues in micelles versus bicelles. “Buried”: *f*_ASA_, the fraction of solvent-accessible residue surface area = 0; “Partially buried”: 0 < *f*_ASA_ ≤ 0.1; “Exposed”: *f*_ASA_ > 0.1. Errors denote means ± SD from fitting. Statistical significance of the difference in correlation slope (*m*) was evaluated using Chow’s test [not significant (NS): *P* ≥ 0.05; *P < 0.05; **P < 0.005). The *r* denotes Pearson’s coefficient. (**C**) Cooperativity profiles mapped onto the GlpG structure. The color code of each residue’s cooperativity profile: “cooperative” (green, ∣ΔΔΔ*G*∣ ≤ *RT* = 0.6 kcal/mol), “moderately localized in N-subdomain” (tin, 2*RT* ≥ ΔΔΔ*G* > *RT*), “localized in N-subdomain” (blue, ΔΔΔ*G* > 2*RT*), “moderately localized in C-subdomain” (orange, −*RT* > ΔΔΔ*G* ≥ −2*RT*), and “localized in C-subdomain” (red, −2*RT* > ΔΔΔ*G*). The cooperativity profiles of 20 residues in micelles have previously been assigned (*60*) (table S3).

Our primary micelle (DDM) and bicelle (DMPC:CHAPS at *q* = 1.5) systems offer comparable hydrophobic thickness matching for GlpG but differ markedly in the strength of amphiphile-amphiphile packing (Fig. 2). To assess whether these distinct properties of the hydrophobic environment influence the contribution of individual residue interactions to membrane protein stability, we performed mutational analysis targeting 37 residues spanning a range of burial depths within the protein interior. Most mutations involved large-to-small side-chain substitutions. The resulting changes in stability ( $∆∆{G{}^{\circ}}_{N-D,\text{WT}-\text{Mut}}$ ), measured independently at N- and C-subdomains (Fig. 3A, left; fig. S13; and tables S2 and S3), were collectively plotted for micelles versus bicelles. The $∆∆{G{}^{\circ}}_{N-D,\text{WT}-\text{Mut}}$ values exhibited a linear correlation with a slope close to unity (*m* = 1.1 ± 0.1), which might suggest that individual residue interactions contribute similarly to GlpG stability in both hydrophobic environments despite their differences in amphiphile packing strength.

The impact of mutations on GlpG stability exhibited subdomain-specific sensitivity to the surrounding hydrophobic environment. Although mutations caused comparable destabilization of N-subdomain in micelles and bicelles (slope *m* = 1.0 ± 0.1) (Fig. 3A, right), the same mutations led to larger destabilization of C-subdomain in bicelles compared to micelles (*m* = 1.3 ± 0.1 with *P* < 0.005 based on Chow’s test in Materials and Methods) (*78*). To investigate the origin of this differential sensitivity, we analyzed the mutational effects based on the degree of residue burial. For residues fully buried in the protein interior (Fig. 3B, left), the fitted slopes exceeded unity in both subdomains (*m* = 1.2 ± 0.2 in N-subdomain and *m* = 1.7 ± 0.2 in C-subdomain), indicating a stronger contribution of buried residues to stability in bicelles. Notably, this effect was more pronounced in C-subdomain than in N-subdomain (*m* = 1.7 versus 1.2), regardless of the location of mutation. These results suggest that the lipid bilayer environment in bicelles more effectively stabilizes the protein core (particularly in C-subdomain) than detergent micelles.

The differential environmental sensitivity of mutational impacts between N- and C-subdomains can be attributed to distinct stabilizing motifs. The stability of C-subdomain is primarily governed by extended backbone-backbone contacts between conserved Gly-zipper motifs (Gly-xxx-Gly-xxx-Gly, where Gly may also be Ala or Ser, and x is any residue) in the TM helices TM4 and TM6 (fig. S14) (*60*, *79*, *80*). The more dehydrated interior of bicelles (Fig. 2D) appears to enhance these weakly polar backbone contacts more effectively than micelles (*81*), leading to greater stabilization of C-subdomain compared to micelles (by −2.0 kcal/mol in Fig. 1F and *m* = 1.7 in Fig. 3B). On the other hand, the stability of N-subdomain mainly relies on the extensive vdW contacts among large aromatic and aliphatic residues such as Trp, Phe, Met, Leu, Val, and Cys (fig. S14). Although this packing interaction also conferred increased stabilization in bicelles relative to micelles, the effect was less pronounced (by −1.2 kcal/mol in Fig. 1F; *m* = 1.2 in Fig. 3B). These findings suggest that the lipid environment more favorably supports backbone-mediated interactions, particularly in C-subdomain, while still modestly enhancing stability driven by side-chain packing in N-subdomain.

As the solvent exposure of mutated residues increased, the correlation slope between stability changes in micelles versus bicelles gradually decreased reaching *m* = 0.5 to 0.7 (Fig. 3B, right). Thus, bicelles attenuated the destabilizing effects of surface mutations more effectively than micelles. It is unexpected that perturbations of the residue interactions at the protein surface influence stability differently depending on the surrounding hydrophobic environment. This result implies that direct residue-amphiphile interactions at the protein surface are as important as residue-residue interactions in the protein interior to stabilizing a protein fold. The lower degree of destabilization caused by mutations at the lipid-contacting protein surface could be explained by two scenarios that are not mutually exclusive: (i) Lipids more effectively compensate for the structural perturbations created by surface mutations than detergents, or (ii) lipids provide a more adaptable solvation environment rendering the stability of protein fold less sensitive to surface perturbations. Our MD simulations support the latter scenario (see below).

### Strengthening of the cooperative network of GlpG by lipid solvation

Do the observed lipid effects on GlpG stability act locally confined to the subdomain or mutated regions, or do they exert global influences on the residue interaction network in the protein structure? To address this, we used cooperativity profiling, an analysis designed to identify whether a given residue engages in local versus cooperative interactions with its structural surroundings (*60*). This experimental approach quantifies how the structural perturbations induced by point mutations spatially propagate within the protein by measuring the differential impact of the mutations on the stabilities of N- and C-subdomains (i.e., $\begin{array}{c} ∆∆∆G{}^{\circ}=∆∆{G{}^{\circ}}_{\text{WT}-{\text{Mut}}^{N}}-∆∆{G{}^{\circ}}_{\text{WT}-{\text{Mut}}^{C}} \end{array}$ ) (*60*). To categorize the cooperativity profile of each residue, we applied four standard threshold values, ∆∆∆G° = −2*RT*, −*RT*, +*RT*, and +2*RT* (*R* is the gas constant, and *T* is the absolute temperature) (Fig. 3C and tables S2 and S3).

Mapping the cooperativity profiles onto the structure of GlpG revealed distinct classes of residue interactions: “cooperative” (mutations induce similar destabilization in both subdomains), “localized” (mutations preferentially destabilizes the subdomain in which they reside), and “overpropagated” (mutations in one subdomain cause larger destabilization in the other). In micelles, cooperative residue interactions clustered into multiple specific regions in the structure (Fig. 3C), notably within the central packing core near the bilayer midplane (Met^100^, Cys^104^, Leu^174^, and Thr^178^) (*60*) and along the narrow water channel (Met^249^, His^150^, and Asn^154^) connected to the catalytic dyad Ser^201^/His^254^ (*82*). Furthermore, a set of residues at the conserved TM4/TM6 interface (Ala^253^, His^254^, Tyr^260^, Gly^261^, Ala^265^, and Asp^268^) harboring the catalytic dyad exhibited overpropagated interactions (*60*).

Notably, the cooperativity map revealed a substantially different pattern in lipid-enriched bicelles (Fig. 3C). Many interactions that appeared localized or overpropagated in micelles turned into cooperative in bicelles. Resultantly, nearly the entire set of residue-packed regions formed a single cooperative unit. When narrower cutoff values ( ∆∆∆G° = −*RT*, −1/2⋅*RT*, +1/2⋅*RT*, and +*RT*) were applied, the resulting cooperativity clusters in bicelles resembled those identified in micelles using the standard thresholds (fig. S15). Thus, the cooperativity architectures in micelles were partially preserved in bicelles.

These findings demonstrate that the lipid environment globally stabilizes the residue interaction network compared to micelles, thereby enhancing the propagation of structural perturbations across the protein. Thus, the observed lipid effects (i.e., the stabilization of the subdomains, the near-uniform subdomain stability, and the favorable residue burial in the protein interior) likely stemmed from the globally reinforced cooperative network of the protein. GlpG catalyzes proteolysis through coordinated motions of multiple structural elements such as L5-cap opening, TM5-gate displacement, substrate engagement at the TM2-TM5 interface, TM6 pivoting against TM3, and L1 rearrangement (Fig. 3C) (*83*–*85*). Our data show that perturbations in these dynamic segments by mutation cooperatively propagate in bicelles, possibly highlighting the integration of these motions into a cohesive interaction network. Notably, bicelles promoted an ~5-fold increase in GlpG activity relative to micelles for both membrane-anchored and soluble substrates (figs. S12 and S16) (*86*). This activity enhancement may be driven by the augmented cooperativity conferred by the lipid environment, which may strengthen the coupling between key conformational changes essential for proteolytic function.

### Inefficient solvation of GlpG by lipids

To understand the molecular basis of the environmental dependence of GlpG stability and cooperativity, we performed all-atom MD simulations of GlpG embedded in either a lipid bilayer or DDM micelles, as well as simulations of micelles alone, extending up to 2.3 μs. For the micelle systems, we selected two aggregation numbers of DDM per protein, *N*_A,DDM_ = 120 (DDM120) and 150 (DDM150) within the experimentally observed range (*N*_A,DDM_ = 90 to 150) (fig. S17) (*64*, *71*). Although a comprehensive evaluation of protein stability ideally requires analysis of both native and denatured states, atomistic modeling and efficient sampling of denatured conformations remain challenging for membrane proteins. Accordingly, our simulations focused on the native conformation of GlpG to examine its structural dynamics in distinct hydrophobic solvation environments.

**Fig. 4.**
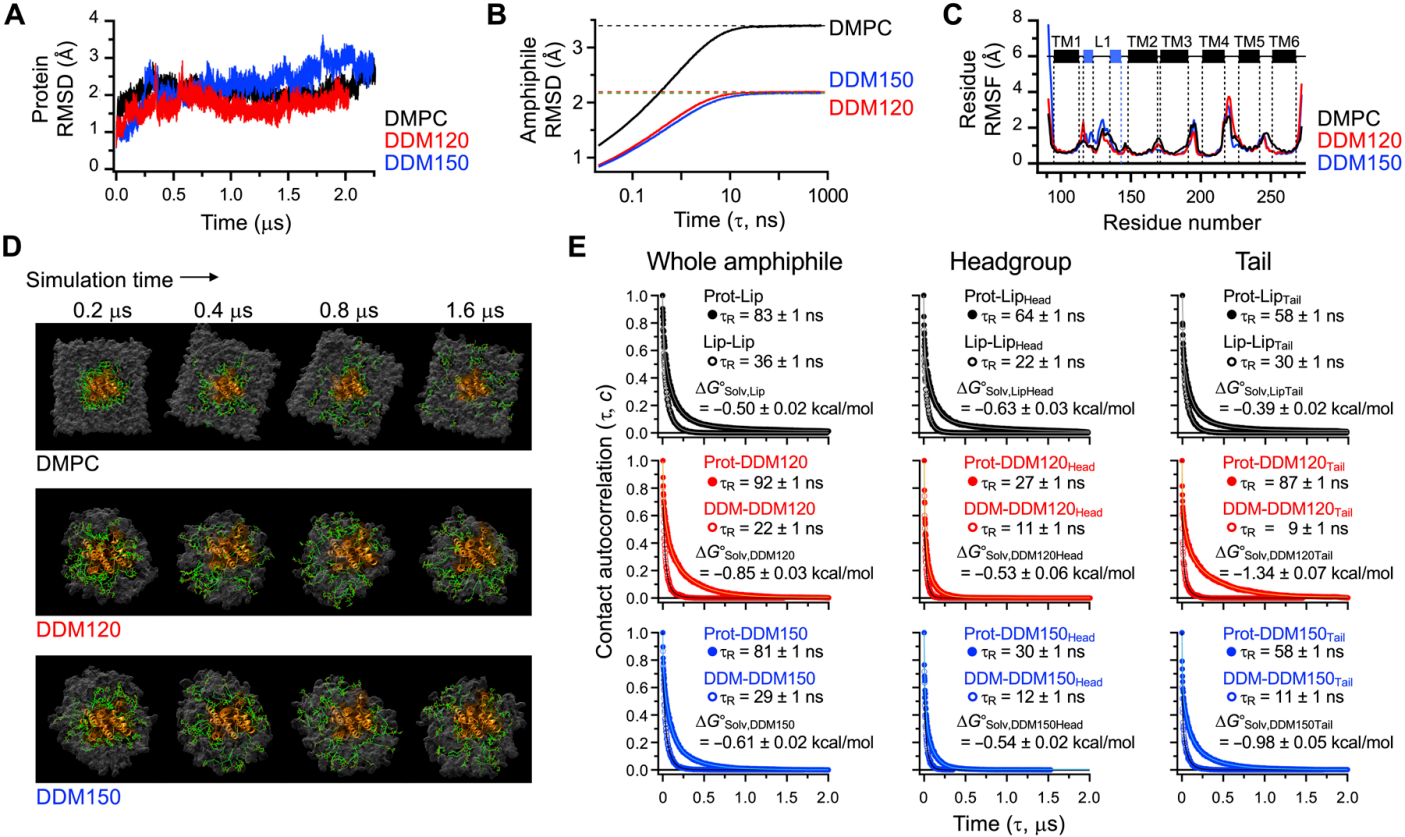
All-atom MD simulation of GlpG in the lipid bilayer and micelles. (**A**) RMSD of backbone heavy atoms. DDM120: *N*_A,DDM_ = 120; DDM150: *N*_A,DDM_ = 150. *N*_A,DDM_ denotes the aggregation number of DDM molecules in each micelle model. (**B**) RMSD(τ) of lipid or detergent conformations in the bulk amphiphilic assemblies as a function of time lag τ. (**C**) Residue root mean square fluctuation (RMSF) in each amphiphile system. (**D**) Structural snapshots showing the solvation dynamics of amphiphiles on GlpG in the DMPC bilayer and DDM micelles. Forty lipid or detergent molecules (green) randomly selected from the first solvation shell of GlpG (orange) at the simulation time, *t* = 0.2 μs, were tracked as a function of time. In all snapshots, the viewing angle and orientation of GlpG (i.e., from the extracellular side) are fixed. (**E**) Time-dependent contact autocorrelation for measuring the residence time (τ_R_) of lipid or detergent molecules on GlpG or on themselves (left). These protein-amphiphile or amphiphile-amphiphile contacts in each system were also separately monitored for the headgroup (middle) and tail (right) regions of amphiphiles. Each data was fitted to a triple-exponential decay function (solid lines) (table S4). Errors denote means ± SD from fitting.

Following conformational equilibration of both GlpG and amphiphiles during simulation (after ~300 ns; Fig. 4, A to C), we examined the solvation dynamics by computing time-dependent contact autocorrelation values for protein-amphiphile and amphiphile-amphiphile interactions (Fig. 4, D and E, and Materials and Methods) (*87*). In all systems, autocorrelation values decayed to below 1%, indicating that intermolecular interactions involving amphiphiles overall reached equilibrium and that both DMPC and DDM molecules predominantly served as solvation amphiphiles transiently associating with the protein. We further quantified the residence times (τ_R_ is the time when the contact autocorrelation amplitude decays to 1/*e* of its initial value) for amphiphiles interacting with the protein and with another amphiphile. Notably, both lipids and detergents exhibited longer residence times on GlpG (80 to 90 ns) than among themselves (20 to 40 ns), indicating that amphiphiles preferentially interact with the protein surface.

Building on the equilibrated amphiphile interactions and the residence time data, we quantified the solvation free energy per amphiphile molecule ( $∆{G{}^{\circ}}_{\text{Solv}}=-\mathrm{RT}$·ln [τ_R,protein-amphiphile_/τ_R,amphiphile-amphiphile_]) by normalizing the residence time of protein-amphiphile contacts to that of amphiphile-amphiphile contacts in bulk (Fig. 4E and table S4). Given the dual hydrocarbon tails of lipid molecules, we anticipated stronger lipid-protein interactions compared to single-tailed detergent molecules. Unexpectedly, DMPC lipids exhibited weaker interactions with GlpG ( $∆{G{}^{\circ}}_{\text{Solv},\text{Lip}}=-0.50\pm 0.02$ kcal/mol) than DDM detergents ( $∆{G{}^{\circ}}_{\text{Solv},\text{DDM}120}=-0.85\pm 0.03$ kcal/mol and $∆{G{}^{\circ}}_{\text{Solv},\text{DDM}150}=-0.61\pm 0.02$ kcal/mol) (Fig. 4E). This weaker lipid interaction was primarily due to the longer τ_R_ of lipid-lipid contacts, coupled with shorter or comparable τ_R_ values for protein-lipid interactions compared to the respective values for detergents. Increasing the aggregation number of DDM from 120 to 150 (i.e., increasing the micelle volume) diminished the strength of protein-detergent interactions. This likely reflects increased detergent-mixing entropy and stronger interaction between detergents in the enlarged micelle, where detergent molecules are tightly packed (fig. S17). In addition, the solvation strength of lipids on the protein involved similar contributions from lipid headgroups and tails, whereas in detergents, tail interactions dominated (Fig. 4E).

MD simulations reveal a counterintuitive insight into protein-lipid interactions as a modulator of membrane protein stability: The lipid bilayer, which is commonly regarded as the native solvent for membrane proteins, acts as a weaker solvent for GlpG than nonnative micelles. This weaker lipid-protein interaction primarily arises from the stronger lipid-lipid interactions relative to detergent-detergent interactions (Fig. 2D), which promotes lipid dissociation from the protein surface (Fig. 4E). Consequently, the reduced strength of lipid-protein interactions shifts the energetic balance in favor of intraprotein interactions, enhancing the stability of the compact native state (Figs. 1 to 3). In this view, the residue interactions needed for stabilizing the protein’s native fold are strengthened due to the weak lipid-protein interaction that would otherwise favorably solvate the denatured state.

### Influence of membrane properties on the folding of OmpLA

Last, we tested whether the modulation of stability and cooperativity by membrane properties is a generalizable hypothesis for the folding of other types of membrane proteins. Accordingly, we used a β-barrel membrane protein, OmpLA, which is evolutionarily unrelated to the helical membrane protein GlpG. The reversible folding of OmpLA has been established using guanidine hydrochloride (GdnHCl) as a denaturant in a 1,2-dilauroyl-*sn*-glycero-3-phosphocholine (DC12PC) bilayer (*88*). Here, we achieved reversible folding in a 1,2-diundecanoyl-*sn*-glycero-3-phosphocholine (DC11PC) bilayer (fig. S18). In their unperturbed states, the DC11PC bilayer exhibits a smaller hydrophobic thickness than the DC12PC bilayer (~18 Å versus ~20 Å) (*89*).

**Fig. 5.**
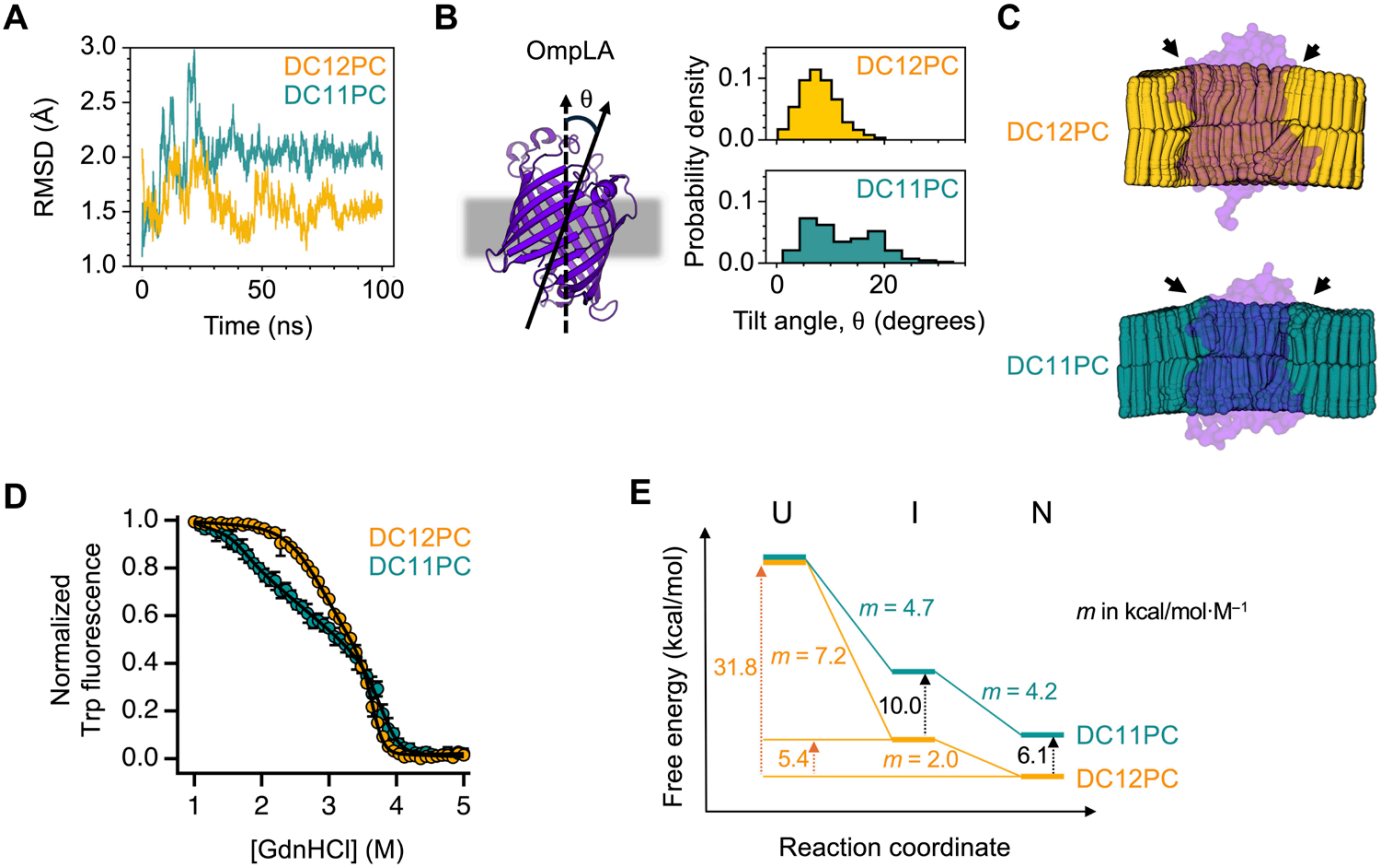
All-atom MD simulations and stability of the β barrel membrane protein, OmpLA, in the DC12PC and DC11PC bilayers. (**A**) RMSD of the backbone C_α_ atoms. (**B**) Distribution of tilt angles (θ) between the molecular axis of OmpLA and the membrane normal in each bilayer system. (**C**) Time-averaged lipid configurations sampled during MD simulations. Black arrows indicate bilayer thickening defects in the DC11PC bilayer. (**D**) Folding titrations of OmpLA. Errors denote ± SEM (*N* = 3 biological replicates). GdnHCl titration data were fitted to a three-state equilibrium folding model (Eq. 19 in Materials and Methods). (**E**) Folding free energy landscape of OmpLA. The free energy level of each state and the *m* value for each transition were obtained from fitting of GdnHCl titration data in (D). The unfolded state (U), which partitions to the aqueous phase in both bilayer systems, was used as a reference state.

We first investigated how the change in bilayer thickness affects the conformational dynamics of OmpLA and the surrounding lipids using all-atom MD simulations (up to ~100 ns). OmpLA in the thinner DC11PC bilayer displays larger backbone fluctuations [root mean square deviation (RMSD): ~2 Å versus ~4 Å] and a broader distribution of tilt angles relative to the membrane normal (θ: 0° to 20° versus 0° to 30°) than in the DC12PC bilayer (Fig. 5, A and B). Moreover, compared to the DC12PC bilayer, time-averaged lipid conformation in the DC11PC bilayer shows stiffening of the aliphatic chains of the solvating lipids, leading to noticeable thickening defects in the extracellular leaflet of the bilayer around the protein (Fig. 5C). These results indicate that the DC12PC bilayer provides a close hydrophobic-thickness matching condition for OmpLA.

The equilibrium folding data were fitted to a three-state model with the native (N), intermediate (I), and unfolded (U) states (*88*) (Fig. 5D and table S5). In the DC12PC bilayer, the transition free energies of OmpLA in the absence of GdnHCl were $∆{G{}^{\circ}}_{N-I,l,w}=-5.4\pm 0.5$ kcal/mol between the N and I states in the membrane and $∆{G{}^{\circ}}_{I-U,l,w}=-26.4\pm 0.1$ kcal/mol between the I and U states in the membrane and aqueous phases, respectively, yielding a total free energy change of $∆{G{}^{\circ}}_{I-U,l,w}=-31.8\pm 0.6$ kcal/mol (Fig. 5E) (*88*). Compared to the DC12PC bilayer, the hydrophobic-mismatching DC11PC bilayer induced substantial destabilization of both the N and I states by 6.1 and 10.0 kcal/mol, respectively (Fig. 5E).

The degree of surface exposure of protein during an unfolding transition is translated into the degree of cooperativity of the transition (*90*). When the surface exposure of OmpLA was evaluated using the *m* value (*d*($∆{G{}^{\circ}}_{\text{unfold}}$)/*d*[GdnHCl]) (Fig. 5E) (*90*), a major portion of the protein surface area was exposed during the I-to-U transition in the DC12PC bilayer (*m*_I-U_ = 7.2 kcal/mol M^−1^ versus *m*_N-I_ = 2.0 kcal/mol M^−1^ for N-to-I). In contrast, similar surface areas were exposed in the N-to-I and I-to-U transitions (*m*_N-I_ and *m*_I-U_ = ~4.5 kcal/mol M^−1^) in the DC11PC bilayer.

In summary, compared to the hydrophobic mismatching condition in the DC11PC bilayer, the hydrophobic matching in the DC12PC bilayer led to stabilization of the native and intermediate states of OmpLA formed in the membrane. In addition, hydrophobic matching induced larger cooperativity in the unfolding transition from the intermediate state in the membrane to the unfolded state in the aqueous phase. These results demonstrate that modulation of protein stability and cooperativity by the physical properties of a bilayer is a common principle for both β barrel and α-helical membrane proteins.

## DISCUSSION

Here, we demonstrated the profound impact of lipid solvation on the stability and cooperativity of membrane proteins. Compared to micelles, lipid-enriched bicelles increase protein stability by promoting residue burial within the protein interior. They also facilitate propagation of local structural perturbations throughout the protein by strengthening the cooperative network. This enhancement of stability and cooperativity is linked to the formation of a lipid bilayer in bicelles, involving improved hydrophobic matching with the protein, increased strength of amphiphile-amphiphile packing, and diminished solvation strength on the protein by amphiphiles, compared to detergent micelles. These physical properties conferred by the lipid bilayer may serve as formative forces shaping the folding energy landscape and cooperative network of membrane proteins in a manner distinct from other types of amphiphilic assemblies. Our results also suggest that, although the fold of a membrane protein is encoded by its primary sequence, the energetics of intraprotein interactions depend on the properties of the hydrophobic environment solvating the protein.

Previously, we have shown that the bilayer induces contraction of the denatured state of GlpG, allowing partial association of TM helices (*10*). In the current study, we observed that the lipid environment facilitates the burial of residues into the protein interior, enhancing protein stability. These results point to a “lipophobic effect” (i.e., an aversion of the protein to lipids) in the membrane, analogous to the hydrophobic effect in water. That is, the lipid environment tends to induce compaction of polypeptide chains inserted into the membrane, although not to the extent of complete collapse (*10*). Our MD simulations predict that the solvation free energy of lipids on the protein ( $∆{G{}^{\circ}}_{\text{Solv}}=$ ~0.5 kcal/mol per lipid molecule) is comparable to the thermal energy (~0.6 kcal/mol). Thus, protein interactions encoded by the amino acid sequence, whether global or local, intra- or intermolecular, specific or nonspecific, can drive compaction if their strengths surpass thermal fluctuations.

Theories suggest that the lipophobic effect stems from perturbations in lipid conformation (e.g., immobilization, stretching, and compression of lipid molecules) around proteins relative to bulk lipids (*91*–*93*). The release of these perturbed lipids into the bulk fluid bilayer is entropically favorable, thereby driving protein association to reduce the total lipid-accessible protein surface area. This entropic effect is expected to influence membrane protein folding across various amphiphilic assemblies, including micelles and bilayers. Our comparative study suggests that lipids have a larger capacity to drive folding than detergents. Although quantitative analysis of this entropic effect is beyond the scope of the current study, we propose an additional physical mechanism based on the observed correlation between GlpG stability and the strength of amphiphile-amphiphile interactions. The denatured state of a helical membrane protein is expanded and thus solvated by a larger number of lipid molecules compared to the compact native state (*10*). When lipid-lipid interactions are strong, the resulting high membrane tension may facilitate folding into the native state by driving the release of excess solvating lipids from the denatured state to the tight lipid-lipid interaction network of the bulk bilayer. This mechanism does not suggest that lipid-induced protein (or helix) association is driven solely by entropy. Instead, the overall strength of lipid-lipid interactions, including both enthalpic and entropic contributions, can shift the energetic balance between folding and denaturation in the membrane.

The hydrophobic thickness mismatch between a bilayer and an embedded protein (or a single-spanning TM helix) has been recognized as an important driving force for protein oligomerization (*24*, *26*–*29*). Our study presents experimental evidence that the same physical force can modulate the stability of multispanning membrane proteins including both α-helical and β barrel types. This modulatory effect observed for lipid bilayers may be generalizable to other types of amphiphilic assemblies. For example, micelles are highly dynamic assemblies undergoing shape fluctuation, chain disordering, and exchange of detergent molecules between the micellar and aqueous phases (*94*). Hence, one might expect that micellar shapes readily adapt to various structural features of membrane proteins. However, we show that, when the intrinsic hydrophobic thickness of a micelle does not match that of a protein, adjusting the micelle’s thickness to the protein’s incurs an energetic cost, leading to protein destabilization (Fig. 2). Thus, physical characterization of amphiphilic assemblies will be beneficial for optimizing membrane protein stability in structural and functional studies (*54*, *64*, *71*, *95*, *96*).

The structures of GlpG crystallized in detergents and bicelles are essentially identical (RMSD: 0.65 Å) (*97*). Moreover, GlpG retains activity in various hydrophobic environments such as micelles, bicelles, liposomes, and nanodiscs (figs. S12 and S16) (*63*, *98*). These results indicate that GlpG has a robust structural fold and that key intraprotein interactions stabilizing the fold are preserved across various hydrophobic environments. If the fold of a membrane protein is not stable enough, however, different environments may induce alternative structures due to the distinct physical constraints that each imposes on the protein. Such examples have been thoroughly discussed in the previous literature (*47*).

The cell membranes are composed of a diverse set of lipids with varying headgroups, aliphatic chain lengths, and degrees of chain unsaturation, collectively conferring a distinct global or local hydrophobic thickness on each membrane (*33*, *99*). An x-ray scattering study indicates that the average hydrophobic thickness of an organelle membrane containing proteins significantly deviates by up to ~5 Å from the thickness of the membrane depleted of proteins (*99*). This suggests that the hydrophobic thicknesses of membrane proteins are not naturally matched to those of the membranes, consistently inducing strain on the membranes (*99*). Recent lipidomic analyses, MD simulation, and protein conformational studies point out that certain types of lipids can preferentially partition around membrane proteins modulating their oligomerization, activity, and conformational equilibrium (*22*, *24*, *63*, *100*, *101*). Considering the negative impact of hydrophobic mismatch on protein stability observed in this study, it is plausible to hypothesize that local membrane deformation by mismatch can be relieved in part by the preferential partitioning of hydrophobically matching lipids to the protein surface in chemically heterogeneous cell membranes (*24*). Such dynamic lipid sorting at the protein-lipid interface may serve to lower the overall free energies of the membrane systems.

Last, the lipid-induced enhancement of cooperativity both sheds light on and cast shadows over the function, folding, and quality control of membrane proteins. Regarding function, many membrane proteins such as ion channels, transporters, receptors, and enzymes require conformational changes (e.g., helix tilting, rotation, domain/subdomain rearrangement, etc.) in response to external stimuli, which often span the entire lengths of the proteins (*46*, *102*–*104*). Our results indicate that the bilayer serves as a conducive medium for the efficient transmission of local structural perturbations throughout the protein structure, facilitating these conformational changes in a cooperative manner and thereby supporting protein function.

In contrast, the efficient propagation of local structural perturbations through a strengthened cooperative network can render the conformational integrity of membrane proteins particularly vulnerable to missense mutations in cell membranes. Most disease-causing mutations in proteins are known to be detrimental to protein stability rather than directly disrupting active-site residues (*105*–*107*). Mapping disease-causing mutations onto the structures of G protein–coupled receptors, ion channels, and transporters shows a strong bias toward mutations occurring in TM regions rather than in the extramembranous regions (*108*). In TM regions, this bias is even more pronounced for residues buried in the protein interior compared to those exposed to lipids (*108*). Our results, showing the amplification of destabilizing effects by internal mutations through the strengthened cooperative network in the bilayer, may provide a physical basis for explaining the biased distribution of disease mutations in the interiors of membrane proteins.

Although we were able to derive a few generalizable principles regarding the role of hydrophobic environments in the folding and cooperativity of membrane proteins, the range of physical properties of the amphiphilic assemblies tested in this study is limited. For example, the hydrophobic thicknesses and strengths of amphiphile packing in our assemblies do not exceed those of the DMPC and DC12PC bilayers for GlpG and OmpLA, respectively. Thus, it remains an open question how the two competing forces (i.e., thickness mismatching as a destabilizing force and increased lipid-lipid interaction strength as a stabilizing force) would affect protein stability if the bilayer thickness further increases. In addition, our study mainly concerns solvating lipids that weakly interact with proteins. The effects of selectively bound lipids on the structure and function of membrane proteins have rigorously been addressed using native mass spectrometry (*109*). Establishing a detergent-free lipid bilayer system for stability measurements will be necessary to investigate broader environmental effects induced by various types of lipids such as cholesterol, charged lipids, unsaturated lipids, nonbilayer-forming lipids, or gel phase-forming lipids. We were able to interpret the stability and cooperativity data reasonably well based on the native structures of the proteins. However, it remains unclear how mutations and the properties of amphiphilic assemblies affect the conformation and free energy of the denatured states. Therefore, to better understand the folding energetics of membrane proteins, it is essential to elucidate how amphiphiles and water molecules solvate the denatured states.

## MATERIALS AND METHODS

### Expression and purification of GlpG

*E. coli* BL21(DE3)-RP cells were transformed with pET21a vector encoding the TM domain of GlpG (residues 87 to 276) (*60*). The cells in Luria-Bertani (LB) broth were grown at 37°C until the optical density at 600 nm (OD_600nm_) reached 0.9. Protein expression was induced by the addition of 0.5 mM isopropyl β-d-thiogalactopyranoside (IPTG), followed by overnight cultivation at 15°C. Resuspension of the total membrane pellet was solubilized by 0.7% (w/v) DDM, 1 mM tris-(2-carboxyethyl)phosphine (TCEP), 0.25 mM phenylmethylsulfonyl fluoride (PMSF), 200 mM NaCl, and 50 mM 2-amino-2-(hydroxymethyl)propane-1,3-diol dihydrochloride (tris-HCl) buffer (pH 8.0). GlpG was purified using nickel-nitrilotriacetic acid (Ni-NTA; Qiagen) affinity chromatography in 50 mM tris-HCl buffer [pH 8.0, 200 mM NaCl and 0.1% (w/v) DDM].

### Biotinylation of GlpG

The double-cysteine variant of GlpG (50 μM; P95C/G172C or G172C/V267C) was incubated with 0.5 mM TCEP for 2 hours at room temperature (24.5° ± 0.5°C). The reaction was incubated overnight at room temperature upon addition of a 40 molar excess of BtnPyr-IA in dimethyl sulfoxide (DMSO). Excess free labels were removed by washing GlpG bound to Ni-NTA resin with 50 mM tris-HCl buffer [pH 8.0, 200 mM NaCl and 0.1% (w/v) DDM] and further by dialysis in the same buffer containing 0.02% (w/v) DDM. Labeling efficiency was determined by measuring the absorbance of pyrene (ε_346nm_ = 42,000 M^−1^ cm^−1^), and the protein concentration by 660-nm assay (Bio-Rad). An SDS–polyacrylamide gel electrophoresis (PAGE) shift assay was carried out on ice without sample heating to identify single mSA-bound, double mSA-bound, and unbound GlpG. Ten microliters of 5 μM GlpG was mixed with 20 μl of the SDS sample buffer followed by the addition of 10 μl of 25 μM mSA-WT (each step incubated for 30 min without heating).

### Preparation of mSA

Detailed procedures were described previously (*60*, *110*). The streptavidin (active or inactive) gene encoded by pET21a vector was expressed as an inclusion body in *E. coli* BL21(DE3)-RP cells. To label mSA with a thiol-reactive dabcyl quencher (dabcyl-maleimide; AnaSpec), Tyr^83^ near the biotin-binding pocket in the active subunit was substituted with cysteine. Active subunit: WT streptavidin or weaker biotin affinity variants (W79M, S45A, S27A, and E51S) with a C-terminal His_6_ tag; inactive subunits: the triple mutant (N23A/S27D/S45A) without His_6_ tag (*111*).

### Expression and purification of the GlpG substrate SN-LYTM2

Procedures for expression and purification of the second TM segment of the *E. coli* lactose permease fused to the C terminus of staphylococcus nuclease (SN-LYTM2) were described previously (*60*). In LYTM2, the residue five residue upstream of the scissile bond was mutated to cysteine to conjugate the thiol-reactive, environment-sensitive fluorophore, iodoacetyl-7-nitrobenz-2-oxa-1,3-diazol (IA-NBD amide; Setareh Biotech). The initial slope of changes in NBD fluorescence versus time represented the proteolytic activity of GlpG. NBD fluorescence was monitored on a SpectraMax M5e plate reader (Molecular Devices) with λ_Ex_ = 485 nm and λ_Em_ = 535 nm.

### Cryo-EM of bicelles and micelles

DMPC:CHAPS bicelles [3% (w/v), *q* = 1.5] and 3% (w/v) DDM micelles were prepared in 40 mM 4-(2-hydroxyethyl)-1-piperazineethanesulfonic acid (Hepes) buffer (pH 7.5, 40 mM KCl) in the absence of GlpG, respectively. Cryo-EM grids were frozen using a Vitrobot Mark IV (Thermo Fisher Scientific). Briefly, 3.5 μl of each sample was applied to a glow-discharged Quantifoil Cu 1.2/1.3 holey carbon 200-mesh grid. The grid was blotted for 3.5 s prior to plunge freezing in liquid ethane. Cryo-EM images were recorded on a Talos Arctica (Thermo Fisher Scientific) operated at 200 kV and equipped with a Falcon 3EC direct electron detector camera. Images were recorded in counting mode using EPU software at a nominal magnification of ×92,000 (1.12 Å/pixel), with a defocus of −2.5 mm. Micrographs were collected as single-frame images with a total exposure time of 1.5 s and a total dose of 30 electrons/Å^2^. A total of 6538 particles from 10 images were autopicked and extracted into 192 pixel–by–192 pixel boxes. The particles were then subjected to two-dimensional (2D) classification using cryoSPARC into 50 classes. The diameters of the bicelles or micelles in each class average were measured to obtain their size distributions.

### Preparation of native and sterically denatured GlpG in micelles

The double-biotin variant of GlpG (20 μM; 95_N_172_M_-BtnPyr_2_ or 172_M_267_C_-BtnPyr_2_) was incubated with a 2.4 times molar excess of mSA_DAB_-E51S in 20 mM Hepes buffer [pH 7.5, 40 mM KCl, 0.5 mM dithiothreitol (DTT), and 5 mM DDM] at room temperature. Denaturation was monitored every 24 hours using GlpG activity as a folding indicator. For 172_M_267_C_-BtnPyr_2_, maximum denaturation was reached in 24 hours. For 95_N_172_M_-BtnPyr_2_, 8 mM SDS was added to facilitate denaturation and incubated for 5 hours.

### Fluorescence quenching assay to measure incorporation of GlpG into bicelles

As a positive control for full incorporation of GlpG in bicelles, DMPC was mixed with dabcyl-1-palmitoyl-2-oleoyl-*sn*-glycero-3-phosphoethanolamine (dabcyl-POPE) (Avanti Polar Lipids) at the molar ratio of 99.5:0.5 in chloroform in a glass tube and dried under a stream of nitrogen. After further drying in vacuum for 4 hours, the lipid mixture was solubilized in 500 μl of 20 mM Hepes buffer (pH 7.5), 5% (w/v) β-octylglucopyranoside (Anatrace) at the final lipid concentration of 7.5% (w/v). GlpG variant 95_N_172_M_-BtnPyr_2_ or 172_M_267_C_-BtnPyr_2_ in DDM was added to the resuspension and incubated on ice for 30 min. Biobeads (Bio-Rad) were added to remove the detergents in three steps [for each step, wet Biobeads (0.2 g/ml) for 6 to 12 hours at room temperature]. Resulting proteoliposomes were extruded 11 times through a 200-nm pore-size membrane. Total lipid concentrations were measured using an organic phosphate assay. On the basis of the lipid concentrations, CHAPS was added to form bicelles of *q* = 1.5. Protein concentrations were measured using a 660-nm assay (Bio-Rad). As a negative control for no incorporation, water-soluble mSA-E51S/Y83C labeled with *N*-(1-pyrene)maleimide (Thermo Fisher Scientific) was used. To prepare an experimental sample, native (“N”) or sterically denatured GlpG (“D⋅mSA_2_”) in DDM was directly injected to the solution of the bicelles containing dabcyl-POPE [20 mM Hepes buffer (pH 7.5) and 40 mM KCl). In the control and experimental samples, the final concentrations of pyrene labels, DDM, and bicelles were adjusted to 1 μM, 5 mM, and 3% (w/v) ([DMPC] + [CHAPS] = 46 mM), respectively. After incubation of the mixtures at room temperature for 24 hours, pyrene fluorescence was measured with λ_Ex_ = 345 nm and λ_Em_ = 390 nm. The degree of quenching, which was related to the degree of GlpG incorporation into bicelles, was determined by the equation, [*F*_Negative control_ − *F*_Experiment_]/[*F*_Negative control_ − *F*_Positive control_] (*F* is the fluorescence intensity of pyrene).

### Measuring the biotin affinity of mSA variants in bicelles

mSA_DAB_-W79M [fluorescence resonance energy transfer (FRET) acceptor], mSA variant with a weaker biotin-binding affinity, was titrated to the 100 nM single-cysteine variant of GlpG singly labeled with BtnPyr (FRET donor) at P95C, G172C, or V267C in 20 mM Hepes buffer [pH 7.5, 3% (w/v) DMPC:CHAPS bicelles, 40 mM KCl, and 0.5 mM DTT]. Pyrene fluorescence was measured with λ_Ex_ = 345 nm and λ_Em_ = 390 nm. After 24 hours, to dissociate bound mSA, excess free biotin was added to 2 mM and incubated for another 24 hours. The measured pyrene fluorescence with free biotin served as a background. Data were averaged from three fluorescence readings (i.e., technical replicates). Background-subtracted data were fitted to Eq. 1 to obtain *K*_d,biotin_ of mSA_DAB_-W79M in bicelles (*60*)

$$\begin{array}{c} \begin{array}{c} F=A1\cdot \\ \frac{\left(P_{T}+\left[\text{mSA}\right]+K_{d,\text{biotin}}\right)-\sqrt{{\left(P_{T}+\left[\text{mSA}\right]+K_{d,\text{biotin}}\right)}^{2}-4P_{T}\cdot \left[\text{mSA}\right]}}{2P_{T}}+A2 \end{array} \end{array}$$ (1)

where *F* is the measured fluorescence intensity, *P*_T_ is the total GlpG concentration, [mSA] is the total mSA_DAB_ concentration, *K*_d,biotin_ is the dissociation constant of mSA_DAB_ from the biotin label, *A*1 is the total net change in pyrene fluorescence, and *A*2 is the fluorescence level without mSA_DAB_. The *K*_d,biotin_ of a stronger biotin-binding mSA variant (W79M, S45A, or S27A) was measured by a FRET-based competition assay. G172C-BtnPyr (1 μM) was pre-equilibrated with a 2 or 5 times molar excess of a dabcyl-labeled mSA variant for 3 hours at room temperature (the quenched state). Next, a weaker biotin-affinity mSA variant without the dabcyl label was titrated against the quenched state and dequenching of pyrene fluorescence was measured. Once equilibrium was reached (24 to 48 hours), free biotin at the final concentration of 2 mM was added to dissociate bound mSA and further equilibrated for 7 to 24 hours. This fluorescence data served as a background signal. Background-subtracted data were fitted to Eq. 2 (*112*)

$$\begin{array}{c} \begin{array}{c} F=A1\cdot \\ \frac{-\left[P_{T}+\left[\text{mSA}\right]+\frac{K_{\text{unlabel}}}{K_{\text{label}}}\cdot \left(C_{T}-P_{T}\right)\right]+\sqrt{{\left(P_{T}+\left[\text{mSA}\right]+\frac{K_{\text{unlabel}}}{K_{\text{label}}}\cdot \left(C_{T}-P_{T}\right)\right)}^{2}+4P_{T}\left[\text{mSA}\right]\cdot \frac{K_{\text{unlabel}}}{K_{\text{label}}}}}{2P_{T}\cdot \frac{K_{\text{unlabel}}}{K_{\text{label}}}}+A2 \end{array} \end{array}$$ (2)

where *K*_unlabel_ is the dissociation constant of mSA without dabcyl and *K*_label_ is the dissociation constant of mSA_DAB_. Fitted values include *A*1, *A*2, and *K*_unlabel_ or *K*_label_. To measure *K*_d,biotin_ of mSA_DAB_-E51S, 1.5 μM G172C-BtnPyr was first titrated with increasing concentrations of mSA-S27A without dabcyl, and pyrene fluorescence was measured. This signal served as a background. Then, 2 μM mSA_DAB_-E51S was added, and quenching of pyrene fluorescence was measured. After reaching an equilibrium (48 hours), the background-subtracted data were fitted to Eq. 2.

### Testing the folding reversibility of GlpG

Native or sterically denatured GlpG in DDM micelles was directly injected into DMPC:CHAPS bicelles [*q* = 1.5, 3% (w/v)] with increasing concentrations of mSA_Dab_-E51S in 20 mM Hepes buffer (pH 7.5, 40 mM KCl, 0.5 mM DTT) to initiate denaturation or refolding at 24.5° ± 0.5°C, respectively. The final concentrations of GlpG, DDM, DMPC, and CHAPS were 0.5 μM, 0.1 to 0.2 mM, 28 mM, and 18 mM, respectively. Thus, there was one DDM molecule in every 225 to 450 DMPC and CHAPS molecules in bicelles. To monitor mSA binding, pyrene fluorescence was measured with λ_Ex_ = 345 nm and λ_Em_ = 390 nm every 24 hours until an equilibrium was reached (48 to 72 hours). In parallel, GlpG activity as a folding indicator was measured at a 20 times molar excess of SN-LYTM2 incorporated in bicelles after the equilibrium was reached.

### Construction of binding isotherms to determine $∆{G{}^{\circ}}_{N-D}$ of GlpG

GlpG (95_N_172_M_-BtnPyr_2_ or 172_M_267_C_-BtnPyr_2_) in DDM was added to bicelle solutions with increasing concentrations of mSA_DAB_ in 20 mM Hepes buffer [pH 7.5, 3% (w/v) bicelles, 40 mM KCl, and 1 mM DTT]. The final concentrations of GlpG, DDM, DMPC, and CHAPS were 1.0 μM, 0.2 to 0.4 mM, 28 mM, and 18 mM, respectively. Thus, there was one DDM molecule in every 112.5 to 225 DMPC and CHAPS in bicelles. Depending on the stability of GlpG mutant, multiple mSA variants with weaker biotin affinities (mSA_DAB_-W79M, mSA_DAB_-S45A, mSA_DAB_-S27A, and mSA_DAB_-E51S) were screened until an optimal second binding phase in the range from 0 to 60 μM [mSA] was obtained. The titrated samples were transferred to a 96-well plate and incubated at 24.5° ± 0.5°C. Binding was measured by quenching of pyrene fluorescence with λ_Ex_ = 345 nm and λ_Em_ = 390 nm until an equilibrium was reached. Data were averaged from three fluorescence readings (i.e., technical replicates).

### Fitting of the second binding phase to obtain $∆{G{}^{\circ}}_{N-D}$ of GlpG

The attenuated second binding of mSA_DAB_ was fitted to the equation derived from the following reaction scheme (*59*, *60*)

$$\begin{array}{c} \begin{array}{l} N\cdot \mathrm{mSA}\overset{K_{D}}{⇄}D\cdot \mathrm{mSA}\;\text{where}\;K_{D}=\frac{\left[D\cdot \mathrm{mSA}\right]}{\left[N\cdot \mathrm{mSA}\right]}\\ D\cdot \mathrm{mSA}+\mathrm{mSA}\underset{K_{d,\text{biotin}}}{⇄}D\cdot 2\mathrm{mSA}\;\text{where}\;K_{d,\text{biotin}}=\frac{\left[D\cdot \mathrm{mSA}\right]\left[\mathrm{mSA}\right]}{\left[D\cdot 2\mathrm{mSA}\right]} \end{array} \end{array}$$ (3)

The fitting equation was

$$F=\frac{1}{\left[1+\left(K_{d,\text{biotin}}+\frac{K_{d,\text{biotin}}}{K_{D}}\right)\cdot \frac{1}{\left[\text{mSA}\right]}\right]}\cdot \left(F_{\infty }-F_{o}\right)+F_{o}$$ (4)

$$∆{G{}^{\circ}}_{N-D}=-\mathrm{RT}\cdot \text{ln}\frac{1}{K_{D}}$$ (5)

where *F* is the measured fluorescence intensity; *F*_o_ and *F*_∞_ are the fluorescence intensities from BtnPyr conjugated to GlpG at [mSA_DAB_] = 0 and at [mSA_DAB_] = ∞, respectively; [mSA] is the total mSA_DAB_ concentration; *K*_d,biotin_ is the unhindered biotin affinity of mSA_DAB_; and *K*_D_ is the equilibrium constant for denaturation of GlpG.

### Proteinase K digestion of native and sterically denatured GlpG

Native or sterically denatured GlpG doubly labeled with BtnRG (*60*) (95_N_172_M_-BtnRG_2_ or 172_M_267_C_-BtnRG_2_) was directly injected into DMPC:CHAPS bicelles [*q* = 1.5, 3% (w/v)] in 20 mM Hepes buffer (pH 7.5, 40 mM KCl and 5 mM DDM) at the final concentrations of 5 μM GlpG and 25 μM mSA-WT. After incubation at room temperature for 24 hours, Proteinase K was added to the final concentration of 3.4 mg/ml. The samples were withdrawn at each time point followed by the addition of 10 mM PMSF to quench proteolysis. DTT (10 mM) was added and incubated for 1 hour to dissociate BtnRG and bound mSA-WT from GlpG by cleaving the disulfide linkage between GlpG and BtnRG. SDS-PAGE was run on ice.

### DEER for native and sterically denatured GlpG

To label the double-cysteine variants (95C172C and 172C267C) of GlpG with the thiol-reactive paramagnetic biotin derivative, BtnRG, the GlpG stock in DDM was diluted to ~50 μM in 1.0% (w/v) DDM and 50 mM Tris HCl (pH 8.0, 200 mM NaCl) and incubated with 2 mM TCEP-HCl for 2 hours at room temperature. BtnRG in DMSO was added at a 40 times molar excess of GlpG to the mixture during gentle vortexing. The labeling reaction was incubated at room temperature overnight in the dark with gentle stirring. After the reaction, excess free BtnRG labels were removed by extensive washing of the labeled proteins bound to Ni^2+^-NTA affinity resin with 50 mM Tris HCl buffer (pH 8.0), 200 mM NaCl, and 0.1% (w/v) DDM. After elution with 500 mM imidazole buffer, imidazole was removed by twice running a desalting column (Bio-Rad) equilibrated with 50 mM Tris HCl (pH 8.0), 200 mM NaCl, and 0.1% (w/v) DDM. The samples were concentrated using a centrifugal concentrator [Millipore; molecular weight cutoff (MWCO) = 30 kDa]. The final protein concentration was determined using OD_280nm_. The labeling efficiency of GlpG was determined using a SDS-PAGE gel shift assay (in the absence of a reducing agent) in the presence and absence of mSA-WT as previously described (*60*). To obtain the sterically denatured state in DDM micelles, 120 μl of GlpG variant 95_N_172_M_-BtnRG_2_ or 172_M_267_C_-BtnRG_2_ (25 μM) was incubated with a 5 times molar excess of mSA-WT in 20 mM Hepes (pH 7.5), 40 mM KCl, and 40 mM DDM at room temperature until the degree of denaturation reached a maximum for 3 days as measured by proteolytic activity of GlpG against the model substrate SN-LYTM2. Then, native (i.e., without mSA) and sterically denatured GlpG were transferred to 3% (w/v) DMPC:CHAPS bicelles (*q* = 1.5). The samples were lastly concentrated using a centrifugal concentrator unit (MWCO = 10 kDa) to 50 to 100 μM as measured by OD_280nm_. Glycerol was added to the final concentration of 10% (v/v) for cryoprotection.

Four-pulse DEER data were collected on a Q-band Bruker ELEXSYS 580 spectrometer using a 150-W amplifier and an E5106400 cavity resonator (Bruker Biospin). The samples were loaded into quartz capillaries and flash frozen in liquid nitrogen prior to data collection at 50 K. The interspin distances were determined from fits to the background-corrected dipolar evolution data using the model-free, nonnegative Tikhonov regularization algorithm on the LongDistances program (https://biochemistry.ucla.edu/Faculty/Hubbell/software.html).

### Fluorescence anisotropy of bicelles and liposomes and determination of the gel-fluid phase transition temperatures

DMPC:CHAPS bicelles [3.0% (w/v), *q* = 0.05, 0.50, 1.19, 1.26, 1.50, and 2.0 with the total amphiphile concentrations of 46 to 48 mM] and DMPC liposomes [3% (w/v), 44 mM DMPC] were prepared with diphenylhexatriene (DPH; the final concentration of 12 μM) incorporated into the bicelles and liposomes. The final volume of each sample was 1.0 ml in 20 mM Hepes buffer (pH 7.5, 40 mM KCl). The conditions of the stocks for preparation of DMPC:CHAPS bicelles and DMPC liposomes were 1.0 ml of DMPC (80 mg/ml) in chloroform, 1 ml of DPH (0.25 mg/ml) in chloroform, and 1 ml of 25% (w/v) CHAPS in 40 mM Hepes buffer (pH 7.5, 40 mM KCl). Proper volumes of the DMPC and DPH stocks were mixed to each glass test tube (13 mm by 100 mm) and dried under a gentle stream of N_2_ gas. Resulting DMPC/DPH films were further dried under vacuum overnight. The samples were then hydrated by the addition of 40 mM Hepes buffer (pH 7.5, 40 mM KCl) and resuspended by vortexing. Last, a proper volume of the CHAPS stock was added followed by vortexing and bath sonication (30 min at 25°C) to form the final bicelle solution. To prepare DMPC liposomes, hydrated lipid/DPH resuspension was extruded 21 times through a 0.2-μm pore-size polycarbonate membrane (Whatman) and stored at 4°C. Fluorescence anisotropy of DPH was measured using a 3 mm–by–3 mm quartz cuvette on a Jasco FP-8350 spectrofluorometer at λ_Ex_ = 350 nm and λ_Em_ = 430 nm with the excitation and emission slit widths of 5 nm (for liposomes, the excitation slit width was set to 2.5 nm). Fluorescence intensities were measured with the excitation and emission polarizers aligned in parallel and then vertical direction to each other. The *G*-factor was measured as a ratio of light scattering intensity (λ_Ex_ = 450 nm and λ_Em_ = 450 nm) measured with parallel to that with vertical polarizer alignment. At each temperature, anisotropy values were measured with the same sample three times (i.e., technical replicates) and averaged. Melting curves were generated for the bicelles and liposomes by measuring temperature-dependent fluorescence anisotropy of DPH over the temperature range from 3.0° to 42.5°C.

We obtained the gel-to-fluid phase transition temperature *T*_m_ by fitting the temperature-dependent fluorescence anisotropy data to a sigmoid function (Igor Pro 6.4, WaveMetrics). The fitted inflection point was taken as *T*_m_

$$r=r_{1}\cdot \frac{1}{1+\text{exp}\left[-\frac{1}{B}\cdot \left(T-T_{m}\right)\right]}+r_{2}$$ (6)

where *r* is the measured anisotropy, *T* is the temperature in degrees Celsius, *B* is a parameter related to the steepness of the transition, *r*_1_ is the total change in anisotropy during the transition, and *r*_2_ is the base anisotropy in the fluid phase. We observed that anisotropy values in the pre- and post-transition temperatures displayed an approximately linear dependence on temperature. To account for this, we set *r*_1_ = *m*_1_⋅*T* + *r*_1,0_ and *r*_2_ = *m*_2_⋅*T* + *r*_2,0_.

### Bicelle preparation for SAXS and Laurdan fluorescence

DMPC:CHAPS and DMPC:DHPC bicelles were formed with mixtures of DMPC (Avanti Polar Lipids), CHAPS (Anatrace), or DHPC (Avanti Polar Lipids). Bicelles were prepared from *q* (molar ratio of lipid to detergent) = 0.1 to 1.0 in increments of 0.1 and from *q* = 1.0 to 2.0 in increments of 0.25 (fluorescence) and 0.5 (SAXS). All samples for fluorescence were prepared at 3% (w/v) total amphiphile in phosphate-buffered saline (PBS; pH 7.4, 8 mM Na_2_HPO_4_, 2 mM KH_2_PO_4_, 137 mM NaCl, and 2.7 mM KCl). Samples for SAXS were prepared at 6% (w/v) total amphiphile in PBS. DMPC and Laurdan in a powder form were dissolved into chloroform stocks before mixing to a molar ratio of 1:400 (Laurdan:amphiphile). Excess chloroform was evaporated with N_2_ gas, and the samples were further dried under vacuum for >16 hours. CHAPS and DHPC detergents in a powder form were dissolved in PBS and subsequently added to the dried lipid film at amounts to achieve specific *q* values. After brought to a final volume of 0.25 ml, samples were vortexed for 60 s and subjected to three freeze-thaw cycles between liquid N_2_ and 40°C, with vortexing for 60 s after each thaw.

### SAXS collection and data processing

Synchrotron SAXS data were collected on the 12-ID-C beamline at the Advanced Photon Source of the Argonne National Laboratory. Samples were loaded into quartz capillary tubes (2.0-mm optical diameter) with 0.01-mm wall thickness (Charles Supper). The incident photon energy was 12 keV (wavelength = 1 Å), and the sample-to-detector distance was adjusted to provide a scattering wave vector range *Q* of 0.006 < *Q* < 1.079 Å^−1^. A mosaic x-ray charge-coupled device detector was used to acquire images with typical exposure times of 1.0 s. A temperature-controlled sample holder with 13 capillary slots was used to record data at 25°C. SAXS measurements with filtered buffer were collected in parallel for background subtraction. 2D scattering data were radially averaged upon acquisition to give the measured scattering intensity *I*(*Q*) as a function of *Q* (Å^−1^). SAXS profiles [*I*(*Q*) versus *Q* (Å^−1^)] from matched buffer conditions were subtracted from sample scattering profiles using SasView (M. Doucet, *et al.* SasView version 4.2.2; available from: http://sasview.org). *Q* values at the second maxima of the scattering profile were used to calculate the dominant headgroup to headgroup distance across the short dimension *L* of lipid-detergent assemblies according to the relationship *Q* = 2*p*/*L* (*p* = *h*/λ, where *p* is the momentum of x-ray, *h* is the Planck’s constant, and λ is the wavelength of x-ray).

### *GP* of Laurdan

DMPC:CHAPS bicelles were doped with a 1:400 molar ratio of Laurdan:amphiphile. Samples were transferred to a 96-well Costar black transparent bottom plate with a final sample volume of 200 μl. Laurdan fluorescence was measured on a Spectromax ID5 Plate Reader (Molecular Devices) using λ_Ex_ = 375 nm and λ_Em_ = 550 nm. Emissions were recorded from 400 to 550 nm in increments of 1 nm. *GP* of Laurdan was calculated using the following equation (*72*)

$$\mathrm{GP}=\frac{I_{440\text{nm}}-I_{490\text{nm}}}{I_{440\text{nm}}+I_{490\text{nm}}}$$ (7)

### Calculation of effective *q* values (*q*_eff_)

The actual lipid-to-detergent molar ratio in bicelles (*q*_eff_) was calculated in two ways. Primarily, the critical concentration of detergents that partition into the aqueous phase (*CBC*) was fixed to 6.5 mM for DHPC or 2.5 mM for CHAPS as experimentally derived (*55*, *57*)

$$q_{\text{eff}}=\frac{{\left[\text{lipid}\right]}_{\text{bicelle}}}{{\left[\text{detergent}\right]}_{\text{bicelle}}}=\frac{{\left[\text{lipid}\right]}_{\text{total}}}{{\left[\text{detergent}\right]}_{\text{total}}-\mathrm{CBC}}$$ (8)

where [lipid]_bicelle_ is the concentration of lipids that partition into the bicellar phase, [detergent]_bicelle_ is the concentration of detergents that partition into the bicellar phase, and [lipid]_total_ and [detergent]_total_ are the total concentrations of lipids and detergents dissolved in solution, respectively. *q*_eff_ was also calculated under the assumption that lipids and detergents are ideally mixed (*95*) (fig. S11)

$$\;\frac{1}{\mathrm{CBC}}=\frac{\chi_{\text{lipid}}}{{\mathrm{CMC}}_{\text{lipid}}}+\frac{\chi_{\text{detergent}}}{{\mathrm{CMC}}_{\text{detergent}}}$$ (9)

where χ_lipid_ and χ_detergent_ are the mole fractions of lipids and detergents out of the total amphiphile concentration, respectively, and *CMC*_lipid_ and *CMC*_detergent_ are the critical micelle concentrations of lipids and detergents, respectively (DMPC: 6 nM; DHPC: 15 mM; and CHAPS: 6 mM from https://avantilipids.com/tech-support/physical-properties/cmcs).

### Statistical analysis

Chow’s test (*78*) (Fig. 3, A and B) evaluates whether the true coefficients (i.e., slopes) in two linear regressions on different subgroups (i.e., the datasets with different degrees of burial of mutated residues) are equal. It tests whether the independent variables (i.e., mutation-induced stability changes in micelles) exert different impacts on the different subgroups of the population (i.e., mutation-induced stability changes in bicelles). The *F*-statistic (*F*) in Chow’s test is defined as *F* = [(*S*_C_ − (*S*_1_ + *S*_2_))/*k*]/[(*S*_1_ + *S*_2_)/(*N*_1_ + *N*_2_ − 2⋅*k*)], where *S*_C_, *S*_1_, and *S*_2_ are the sum of squared residuals for the combined subgroups, subgroup 1, and subgroup 2, respectively; *N*_1_ and *N*_2_ are the number of points in subgroup 1 and subgroup 2, respectively; and *k* and (*N*_1_ + *N*_2_ − 2⋅*k*) are the first and second degrees of freedom (the number of datasets, *k* = 2), respectively. The *P* value was calculated using the *F*-statistic and the degrees of freedom, where *P* = 1 − *f.dist*(*F*-statistic, *k*, *N*_1_ + *N*_2_ − 2⋅*k*, TRUE). On the 5% significance level, the null hypothesis cannot be rejected if *P* > 0.05.

### Cooperativity profiling

Specific residue interaction was perturbed by a single point mutation in the background of the double-biotin variants 95_N_172_M_-BtnPyr_2_ or 172_M_267_C_-BtnPyr_2_, which was set as “WT.” Then, the stability change induced by the same mutation was measured by steric trapping for each WT background ( $\begin{array}{c} ∆∆{G{}^{\circ}}_{N-D,\text{WT}-{\text{Mut}}^{N}}=∆{G{}^{\circ}}_{N-D,{\text{WT}}^{N}}-∆{G{}^{\circ}}_{N-D,{\text{Mut}}^{N}} \end{array}$ or $\begin{array}{c} ∆∆{G{}^{\circ}}_{N-D,\text{WT}-{\text{Mut}}^{C}}=∆{G{}^{\circ}}_{N-D,{\text{WT}}^{C}}-∆{G{}^{\circ}}_{N-D,{\text{Mut}}^{C}} \end{array}$ ). Then, the differential effect of the mutation on the stability of the two subdomains is quantified as follows (*60*)

$$\begin{array}{c} \begin{array}{c} ∆∆∆G=\left[∆{G{}^{\circ}}_{N-D,{\text{WT}}^{N}}-∆{G{}^{\circ}}_{N-D,{\text{Mut}}^{N}}\right]-\left[∆{G{}^{\circ}}_{N-D,{\text{WT}}^{C}}-∆{G{}^{\circ}}_{N-D,{\text{Mut}}^{C}}\right]\\ \;=∆∆{G{}^{\circ}}_{N-D,\text{WT}-{\text{Mut}}^{N}}-∆∆{G{}^{\circ}}_{N-D,\text{WT}-{\text{Mut}}^{C}} \end{array} \end{array}$$ (10)

We applied four standard cutoff values, ΔΔΔ*G* = −2*RT*, −*RT*, *RT*, and 2*RT* (*R* is the gas constant, and *T* is the absolute temperature). For a given ΔΔΔ*G* value, the cooperativity profile was assigned as follows: +2*RT* < ΔΔΔ*G*: highly localized in N-subdomain; +*RT* < ΔΔΔ*G* ≤ +2*RT*: moderately localized in N-subdomain; −*RT* ≤ ΔΔΔ*G* ≤ +*RT*: cooperative; −2*RT* ≤ ΔΔΔ*G* < −*RT*: moderately localized in C-subdomain; and ΔΔΔ*G* < −2*RT*: highly localized in C-subdomain.

### MD simulation of GlpG

MD simulation setups were based on the crystal structure of *E. coli* GlpG [Protein Data Bank (PDB) code: 2IC8] (*80*). The bicelle was approximated to a lipid bilayer composed of 315 DMPC molecules, which was constructed using the CHARMM-GUI membrane builder (*113*). Two micellar systems were built with 120 (DDM120) and 150 (DDM150) DDM molecules per micelle, modeled by symmetrically enclosing the TM domain of GlpG with DDM molecules (*114*). Each of the GlpG-bilayer and GlpG-micelle composite systems were immersed in the TIP3P water solvent, followed by charge neutralization and ionization with 150 mM NaCl. Each system was composed of >90,000 atoms in a 115 Å–by–115 Å–by 89 Å box. Independently, we prepared DDM120 and DDM150 micelles without GlpG as controls. All inter- and intramolecular interactions were enumerated under the CHARMM36 force field (*115*). Nonbonding vdW and short-range electrostatic interactions were treated with a typical cutoff distance of 12 Å, whereas the long-range electrostatic contributions were evaluated with the particle-mesh Ewald method. All simulations were carried out using GROMACS software (*116*) parallelized in the GPU-accelerated IBM Power8 machine. Each system was first subject to 10,000 steps of conjugate gradient energy minimization to remove any unfavorable atomic crash with lipids and GlpG, which were restrained to preserve their conformation and relative positions. The systems were pre-equilibrated along six scheduled steps as gradually removing the external restraints until no constraints. Simulations were proceeded with a 2-fs timestep in the semi-isotropic isobaric and isothermal ensemble of 1 atm (101.325 kPa) and 310 K, where the pressure and temperature were controlled by a Parrinello-Rahman barostat and Nosé-Hoover thermostat, respectively. Pressure was decoupled between the *xy* plane and the *z* axis, so the membrane normal fluctuated independently from the isotropic lateral motions (*xy* plane).

### Assessing the equilibration of protein and amphiphiles

Equilibration of GlpG conformation was examined by calculating the RMSD’s of all heavy atoms referenced to the crystal structure. Regarding the equilibration of amphiphile conformation, we assessed time-autocorrelated RMSD(τ) as a function of time lag τ by averaging over all lipid or detergent molecules in bulk as follows

$$\text{RMSD}\left(\tau \right)=\frac{1}{N_{L}}\sum_{i=1}^{N_{L}}<{\text{RMSD}}_{i}\left(t,t+\tau \right){>}_{t}$$ (11)

where *N_L_* is the number of lipid or detergent molecules and <*RMSD_i_* (*t*, *t* + τ)>*_t_* is the heavy-atom RMSD between the *i*th amphiphile’s conformations at the time *t* and *t* + τ, averaged over all available time *t*’s. Bulk lipid molecules were selected from ones not in contact with the protein over the analysis period, whereas bulk detergent molecules were from the control micelles in the absence of GlpG. Before the RMSD calculation, the amphiphiles under comparison at each *t* and *t* + τ were structurally aligned with each other by transrotating heavy-atom conformations.

To assess the solvation dynamics of interfacial amphiphiles on GlpG, we computed the autocorrelation function on time, *c*(τ) for amphiphile heavy atoms within 5 Å from GlpG as follows

$$c\left(\tau \right)=\frac{1}{N_{c}}\sum_{i=1}^{N_{c}}<\;c_{i}\left(t,t+\tau \right)\;{>}_{t}$$ (12)

where *N*_c_ is the number of contact events. A single contact event was defined as a consecutive contact of an amphiphile molecule with no noncontacting time gap longer than the amphiphile relaxation time as measured above. The autocorrelation function at the time τ of the *i*th contact event, <*c_i_* (*t*, *t* + τ)>*_t_*, was defined by <*q_i_*(*t*)⋅*q_i_*(*t* + τ)/*q_i_*^2^(*t*)>*_t_* representing the normalized product of heavy atom contact numbers of an amphiphile molecule in the *i*th contact event, *q_i_*(*t*) and *q_i_*(*t* + τ), at two time moments, *t* and *t* + τ, respectively, averaged over the time *t*.

### Residence time of amphiphiles

Residence time (τ_R_) was defined as the time when the amplitude of contact autocorrelation reached 1/*e* of the initial value without any assumption on dissociation mechanisms based on Eq. 12. Dissociation dynamics of an amphiphile from GlpG was assessed as a whole molecule or parts (i.e., headgroup and tail). The tail of DMPC was defined as the atoms in two aliphatic chains (C22-C214 and C32-C314), whereas that of DDM included all carbons in the dodecyl chain (C1-C12), thus leaving the rest as the headgroup. As a control, we assessed self-dissociation of an amphiphile from another amphiphile, where the pairs were selected from the molecules in contact with each other in bulk. DMPC molecules were selected from ones with no explicit contact with the protein in the bilayer, and DDM molecules were from the micelles in the absence of GlpG. In each system, the residence time for self-dissociation (i.e., the separation between two amphiphiles) served as a reference for evaluating the preference of an amphiphile for the protein relative to other amphiphiles.

### Solvation free energy ( $∆{G{}^{\circ}}_{\text{Solv}}$ ) of amphiphiles on GlpG

The average residence time (τ_R,P·L_) of amphiphiles on GlpG was computed based on the time-autocorrelation function for each amphiphile contacting GlpG. This analysis is extended over all peripheral area of GlpG, covering the entire network of possible amphiphile-protein binding events

P·L(1)→koff,P·L(1)P+L(1)P·L(2)→koff,P·L(2)P+L(2)⋮P·L(i)→koff,P·L(i)P+L(i)⋮P·L(Nc)→koff,P·L(Nc)P+L(Nc) (13)

where P·L_(*i*)_ is the amphiphile-protein complex at the site (*i*) on the protein and L_(*i*)_ represents the amphiphile at the same site. The corresponding dissociation rate constant, *k*_off, P·L(*i*)_, was obtained from the relationship, *k*_off,P·L(*i*)_ = 1/τ_R,P·L(*i*)_, where τ_R,P·L(*i*)_ denotes the residence time of an amphiphile *L*_(*i*)_ at site (*i*) using Eq. 12. Meanwhile, dissociation reactions of amphiphile-amphiphile complexes in the bulk was considered

$$L\cdot L_{\left(j\right)}\overset{k_{\text{off},L\cdot L_{\left(j\right)}}}{\to }L_{\left(j\right)}+L_{\left(j\right)}$$ (14)

L·L_(*j*)_ denotes the *j*th amphiphile-amphiphile complex found in the bulk where *j* = 1,…, *N*_L·L_ with the corresponding dissociation constant, and *k*_off,L·L(*j*)_ = 1/(τ_R,L·L(*j*)_), where τ_R,L·L(*j*)_ is the residence time of the same amphiphile-amphiphile complex. The averaged dissociation rate constant, *k*_off,L·L_, was obtained from the residence time, τ_R,L·L_, which was averaged over the contact autocorrelation function analysis (Eq. 12) of all available amphiphile-amphiphile complexes.

Thus, the solvation free energy, $∆{G{}^{\circ}}_{\text{Solv}\left(i\right)}$ , of an amphiphile at a specific site i on GlpG can be obtained from the following relationship

$$\begin{array}{c} ∆{G{}^{\circ}}_{\text{Solv}\left(i\right)}=-\mathrm{RT}\text{ln}\frac{k_{\text{off},L\cdot L}}{k_{\text{off},P\cdot L_{\left(i\right)}}}=-\mathrm{RT}\text{ln}\frac{\tau_{R,P\cdot L_{\left(i\right)}}}{\tau_{R,L\cdot L}} \end{array}$$ (15)

However, we were interested in the interactions between the protein and amphiphile molecules across all possible contact sites rather than at specific sites. Thus, the averaged solvation free energy, $∆{G{}^{\circ}}_{\text{Solv}}$ , was evaluated by incorporating all site-specific solvation free energies, $∆{G{}^{\circ}}_{\text{Solv}\left(i\right)}$ , as follows

$$∆{G{}^{\circ}}_{\text{Solv}}=-\mathrm{RT}\text{ln}\left(\frac{1}{N_{c}}\;\sum_{i}e^{-∆{G{}^{\circ}}_{\text{Solv}\left(i\right)}/\mathrm{RT}}\right)$$ (16)

where *N*_c_ is the number of potential interaction sites. This formulation accounts for thermal fluctuations and the ensemble of possible amphiphile positions on the protein, resulting in a comprehensive description of $∆{G{}^{\circ}}_{\text{Solv}}$ . The summation over different sites allows for the incorporation of the extensive network of interactions and the competition between amphiphile-amphiphile and amphiphile-protein associations, not merely the exchange at a single site. This is linked to the dissociation reactions of amphiphiles occurring at various sites on the protein through the following relationship

∆G°Solv=−RTln(1Nc∑ie−∆G°Solv(i)/RT)=−RTln(1Nc∑ikoff,L·Lkoff,P·L(i))=−RTln(1Nc∑iτR,P·L(i)τR,L·L)=−RTln(1τR,L·L∑iτR,P·L(i)Nc)=−RTlnτR,P·LτR,L·L=−RTlnkoff,L·Lkoff,P·L=−RTlnKSolv (17)

Notably, the average residence time of amphiphiles on the protein, $\frac{\sum_{i}\tau_{R,P\cdot L_{\left(i\right)}}}{N_{c}}$ , was obtained from the contact autocorrelation analysis (Eq. 12) of amphiphile-protein interactions. In summary, this analysis accounts for the residence times of all amphiphiles interacting with the protein (τ_R,P·L_) and compares it with that of amphiphiles interacting with each other (τ_R,L·L_), as expressed in the following equation

$$\begin{array}{c} ∆{G{}^{\circ}}_{\text{Assoc}}=-\mathrm{RT}\text{ln}K_{\text{Assoc}}=-\mathrm{RT}\text{ln}\frac{k_{\text{off},L\cdot L}}{k_{\text{off},P\cdot L}}=-\mathrm{RT}\text{ln}\frac{\tau_{R,P\cdot L}}{\tau_{R,L\cdot L}} \end{array}$$ (18)

Thus, $∆{G{}^{\circ}}_{\text{Solv}}$ is not merely a representative of an exchange energy at a single site but considers the solvation free energies for protein-amphiphile interactions occurring at all potential interaction sites presented by the protein.

### Expression and purification of OmpLA

We expressed and purified OmpLA following established protocols (*88*, *117*). The HMS174 *E. coli* cells with OmpLA plasmid were inoculated in 500 ml of LB media. At OD_600nm_ = 1.0, the cells were induced with 100 μM IPTG. After 6 hours of expression, the cells were harvested and lysed in 30 ml of lysis buffer (50 mM Tris and 40 mM EDTA, pH 8.0) by an Emusiflex pressure homogenizer. Thirty-five microliters of Brij-35 [30% aqueous solution (w/w)] was added to 35 ml of cell lysate before centrifugation. The pellet was collected after spinning and washed three times with wash buffer (10 mM Tris and 1 mM EDTA, pH 8.0). The purified inclusion body was aliquoted and stored at −20°C.

### Folding of OmpLA

OmpLA was folded into 1,2-diundecanoyl-*sn*-glycero-3-phosphocholine (DC11PC) or 1,2-dilauroyl-*sn*-glycero-3-phosphocholine (DC12PC) vesicles following established protocols (*88*, *117*). The inclusion body of OmpLA was dissolved in 8 M GdnHCl with 100 mM citrate buffer (pH 3.8). The protein concentration was adjusted to 100 μM after centrifugation and filtering of the supernatant with unfolded OmpLA to remove undissolved aggregates. The OmpLA stock was first diluted to 6 μM in 2.5 M GdnHCl with 1.4 mM sulfobetaine-14 using 100 mM citrate buffer (pH 3.8). Diluted OmpLA was further dropwisely diluted to 2 μM in 1 M (folding direction) or 5 M (unfolding direction) GdnHCl, in the presence of 4 mM DC12PC or DC11PC vesicles prepared by extrusion in citrate buffer. The dilutions were performed on a hot plate while stirring at 400 rpm and 42°C. After overnight incubation at 37°C, the samples were diluted for the second time to a range of final GdnHCl concentrations from 1 to 5 M and then incubated for another 40 hours at 37°C. The unfolded fractions of OmpLA were determined by the intrinsic fluorescence of lipid-facing Trp residues.

### Fitting titration data

Fluorescence data were normalized and averaged before fitting. The normalized fluorescence data were fitted to a three-state model as described previously (*88*, *117*)

$$Y_{\text{obs}}=\frac{Y_{N}+Y_{I}K_{N-I}+Y_{U}K_{N-I}K_{I-U}}{1+K_{N-I}+K_{N-I}K_{I-U}}$$ (19)

where *Y*_obs_ is the observable, which is the normalized fluorescence; *Y*_N_, *Y*_I_, and *Y*_U_ are the baselines for the native (N), intermediate (I), and unfolded (U) states (*Y*_X_ = *I*_X_ + *S*_X_·[D], where X = N, I, or U; *I*_X_ and *S*_X_ are the intercept and slope of the baseline, respectively; and [D] is the concentration of GdnHCl); and *K*_N-I_ and *K*_I-U_ are the equilibrium constants of the N to I and I to U transitions, respectively. By expressing the free energy change of each transition as a linear function of [D], the equilibrium constants were written as

$$K_{N-I}=\text{exp}\left(\frac{-\left(∆{G{}^{\circ}}_{N-I,l,w}-m_{N-I}\left[D\right]\right)}{\mathrm{RT}}\right)$$ (20)

$$K_{I-U}=\text{exp}\left(\frac{-\left(∆{G{}^{\circ}}_{I-U,l,w}-m_{I-U}\left[D\right]\right)}{\mathrm{RT}}\right)$$ (21)

where $∆{G{}^{\circ}}_{N-I,l,w}$ and $∆{G{}^{\circ}}_{I-U,l,w}$ are the transition free energies in water, *R* is the gas constant; *T* is the temperature, and *m*_N-I_ and *m*_I-U_ are the dependence of the transition free energies on GdnHCl concentrations. For the titrations with DC12PC, we used fixed *m* values previously determined by global fitting on WT and many variants (i.e., *m*_N-I_ = 2.0 kcal mol^−1^ M^−1^ and *m*_I-U_ = 7.2 kcal mol^−1^ M^−1^). For the titrations with DC11PC, we floated both *m* values during fitting.

### MD simulations of OmpLA

We performed all-atom MD simulation with the CHARMM36m force field using NAMD. We made the initiation files using CHARM-GUI with a similar setup as previously described (*117*). Briefly, we built the system of OmpLA (PDB code: 1QD5) (*118*) embedded in the DC12PC or DC11PC bilayer (~75 lipid molecules at each leaflet) with 0.1 M KCl at 1 atm (101.325 kPa) and 37°C. After six initial equilibration steps, we continued to equilibrate the system for another 50 ns of production run. At least 150-ns run was performed for both DC12PC and DC11PC systems. We collected the data from the last 100-ns simulations for analysis. The simulation trajectories of OmpLA and lipids were analyzed with MDAnalysis (*119*) and MOSAICS (*120*). Specifically, the “membrane protein tilt angle” and “average lipid conformation” tools were used from MOSAICS. For the average lipid conformation analysis, the membrane was projected onto a grid lattice around the protein. The coordinates of every lipid molecule that visited a certain lattice point were time-averaged over the trajectory. The resulting visualization included nonphysical structures due to the averaging out of the rotational dynamics of lipids, but it represented the overall position and shape of the lipids in the system.
