# Supplementary material for: The lipid bilayer strengthens the cooperative network of membrane proteins
Source: Sci Adv. 2025 Jul 2;11(27):eadv9568. doi: 10.1126/sciadv.adv9568 (PMC12219509; doi:10.1126/sciadv.adv9568)
Supplement: Supplementary file 1 — Figs. S1 to S18 Tables S1 to S5 Legend for dataset S1 [file sciadv.adv9568_sm.pdf]

Science Advances
AAAS

Supplementary Materials for

The lipid bilayer strengthens the cooperative network of membrane proteins

Shaima Muhammednazaar *et al.*

Corresponding author: Heedeok Hong, honghd@msu.edu; Seung-gu Kang, sg.kang@skku.edu;
Linda Columbus, columbus@virginia.edu; Karen G. Fleming, karen.fleming@jhu.edu

Sci. Adv. **11**, eadv9568 (2025)
DOI: 10.1126/sciadv.adv9568

The PDF file includes:

Figs. S1 to S18
Tables S1 to S5
Legend for dataset S1

Other Supplementary Material for this manuscript includes the following:

Dataset S1

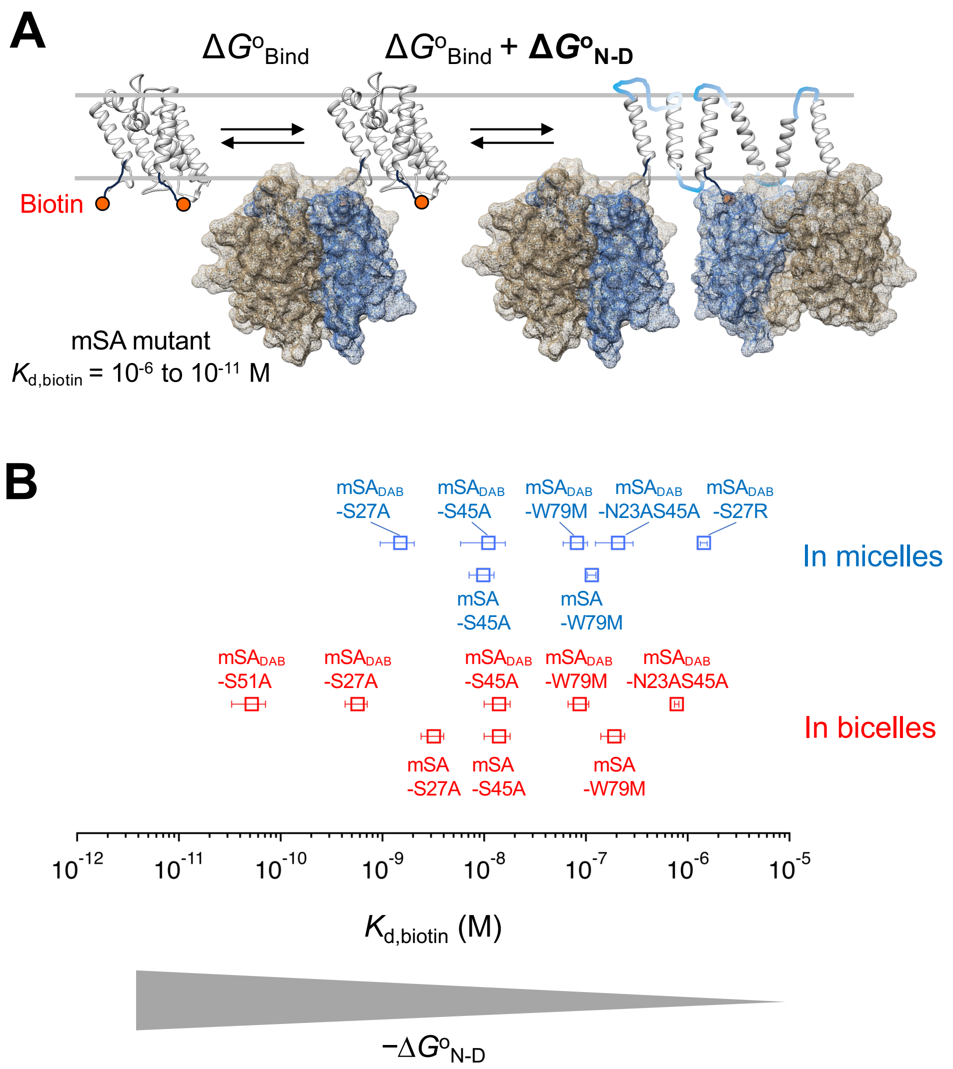

Fig. S1. Principles of steric trapping. (A) The reaction scheme for measuring the thermodynamic stability ($\Delta G^{o}_{\text{N-D}}$) of GlpG. The subunits of monovalent streptavidin (mSA) are color-coded (the active subunit in blue and inactive subunit in grey). **(B)** (*Top*) The intrinsic biotin affinities ($K_{\text{d,biotin}}$) of mSA variants in DDM micelles and DMPC:CHAPS bicelles. mSA variant with a reduced biotin affinity is used to achieve the reversible coupling between mSA binding and GlpG denaturation. Site-directed mutagenesis on the active subunit of mSA modifies the $K_{\text{d,biotin}}$ (*60, 61*). $K_{\text{d,biotin}}$'s are measured using direct titrations of the singly biotinylated variant (172$_{\text{M}}$-BtnPyr) with quencher-labeled mSA (mSA$_{\text{DAB}}$) or a competition assay between mSA$_{\text{DAB}}$ and unlabeled mSA (see **fig. S4**). For determining the stability of a given membrane protein, a mSA variant is chosen when inducing an optimal attenuation of the second mSA binding phase ($\Delta G^{o}_{\text{Bind}}$ + $\Delta G^{o}_{\text{N-D}}$) within the practical [mSA$_{\text{DAB}}$] range of 0 µM to ~60 µM, that is, the simultaneous binding of the mSA variant to the double biotin labels on GlpG effectively competes with the folding of GlpG (i.e., the second reaction step in **fig. S1A**). (*Bottom*) Thus, mSA variant with a higher biotin affinity (i.e., a lower $K_{\text{d,biotin}}$) is used to measure higher stability ($-\Delta G^{o}_{\text{N-D}}$) and *vice versa*. Stability measurable with a given $K_{\text{d,biotin}}$ of mSA variant is represented by the vertical width in the grey arrowhead.

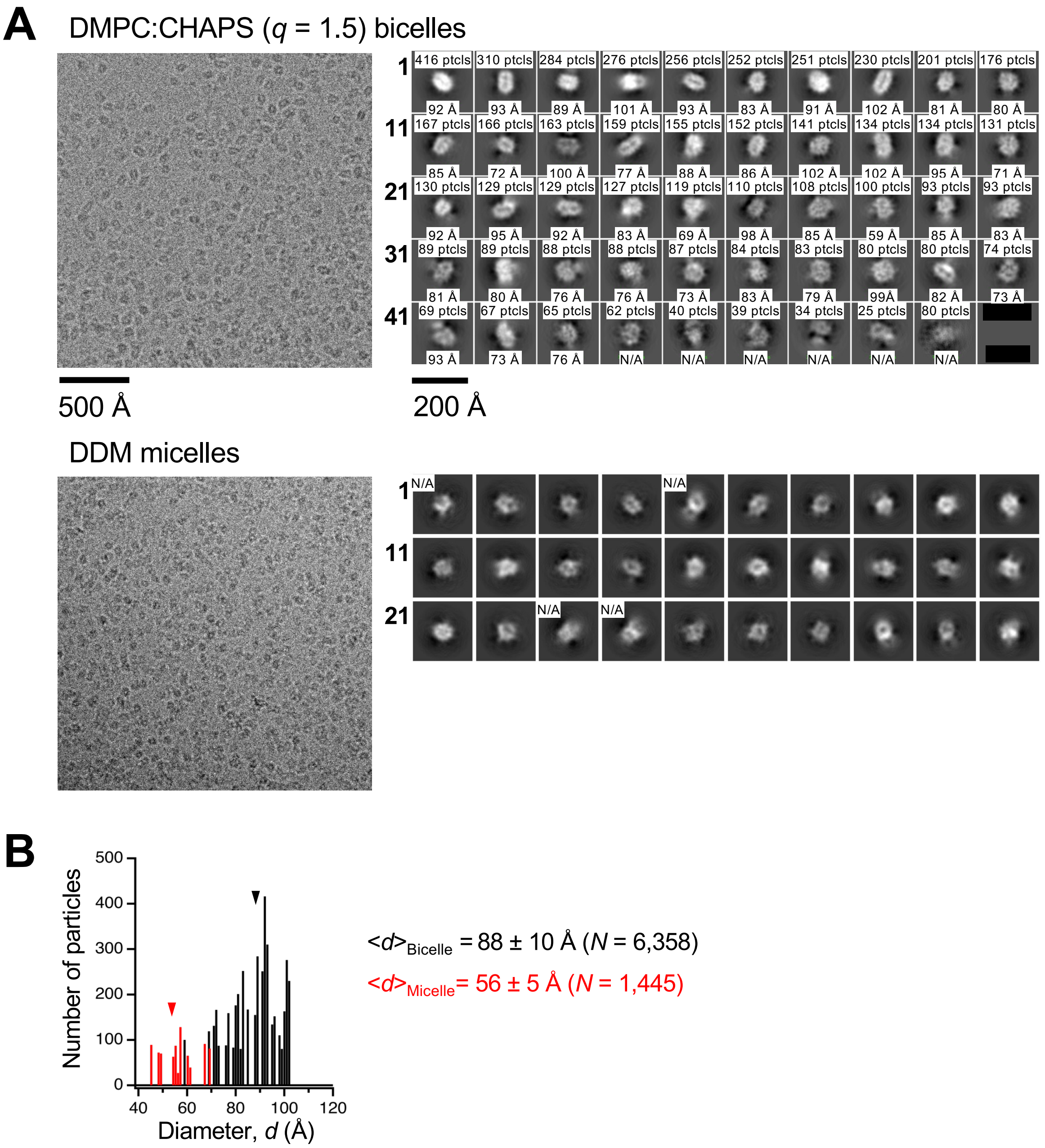

Fig. S2. Cryo-EM analysis of bicelles and micelles without GlpG. (A) Selected raw images (*left*) and 2D class averages (*right*). For bicelles (*top*), forty-nine 2D-class average images were obtained from $N = 6{,}358$ particles (ptcls). For micelles, thirty 2D-class averages were obtained from $N = 1{,}445$ particles. Bicelles and micelles were formed in 20 mM HEPES buffer (pH 7.5, 40 mM KCl, and 1 mM DTT). In each 2D-class average panel for bicelles, the number of particles selected for averaging and the maximal particle length are shown. Maximal particle lengths were not determined for the panels marked with "N/A" due to the blurring of particle borders or the overlapping of multiple particles. **(B)** Size analysis based on 2D-class averages. Errors denote means ± SD. The mean diameter values for micelles ($\langle d \rangle_{\mathrm{micelles}}$) and bicelles ($\langle d \rangle_{\mathrm{bicelles}}$) are marked as arrowheads.

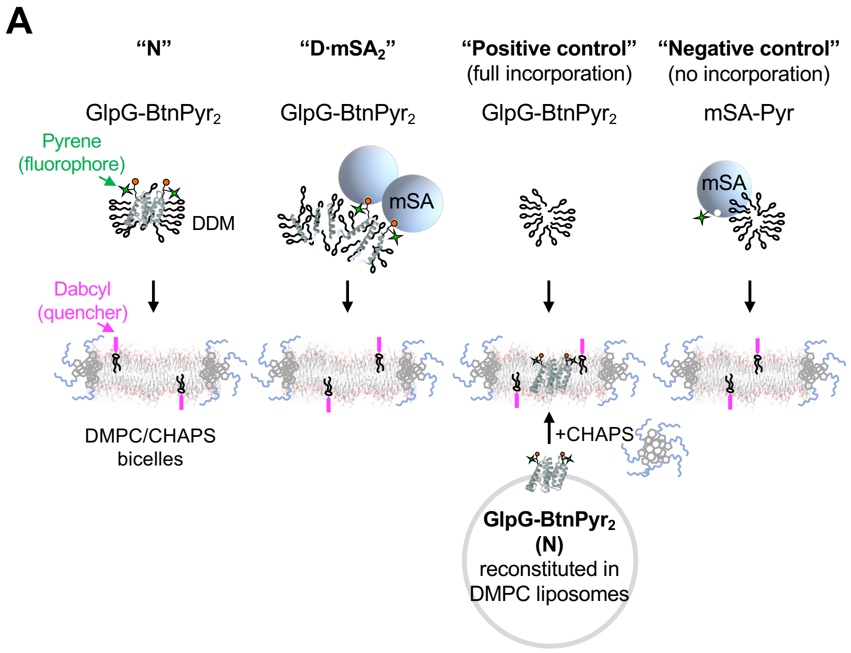

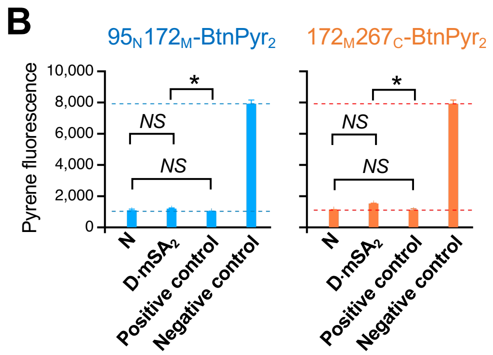

C

Construct	Incorporation ratio (%) N injected	Incorporation ratio (%) D·mSA$_2$ injected
95$_N$172$_M$-BtnPyr$_2$	99.1 ± 1.1	97.6 ± 0.6
172$_M$267$_C$-BtnPyr$_2$	99.5 ± 1.3	93.7 ± 1.3

Fig. S3. Incorporation of native and sterically denatured GlpG into bicelles. (A) Schematic description of a fluorescence quenching assay to measure the incorporation of native ("N": the native, doubly biotinylated variants, GlpG-BtnPyr$_2$) and sterically denatured GlpG ("D·mSA$_2$") in DDM micelles into bicelles (3 w/v-%). As a "positive control" representing full incorporation of the protein, GlpG-BtnPyr$_2$ was reconstituted in DMPC liposomes first and then the resulting proteoliposomes were solubilized by CHAPS to form bicelles. As a "negative control" representing no incorporation, the water-soluble mSA-Y83C variant labeled with thiol-reactive pyrene-maleimide was added to bicelles. In the samples for both "positive" and "negative" controls, DDM was added to the final concentration comparable to the "N" and "D·mSA$_2$" samples (0.01 w/v-% to 0.02 w/v-%). **(B)** The assay results. Incorporation of GlpG-BtnPyr$_2$ to bicelles induced quenching of pyrene fluorescence. Error bars denote ± SEM (N = 3 biological replicates). The p-values from two-tailed, pairwise student's t-tests are shown (*NS*: p>0.05; *: p<0.05). This test was chosen because there was no assumption for the direction of changes in fluorescence signal from the means. **(C)** Incorporation ratios of native and sterically denatured GlpG from the micellar to the bicellar phase. The incorporation ratio = [F ("negative control") – F ("N")]/[F ("negative control") – F ("positive control")]. F indicates fluorescence intensity at 390 nm in each sample. Errors denote ± SEM (N = 3 biological replicates).

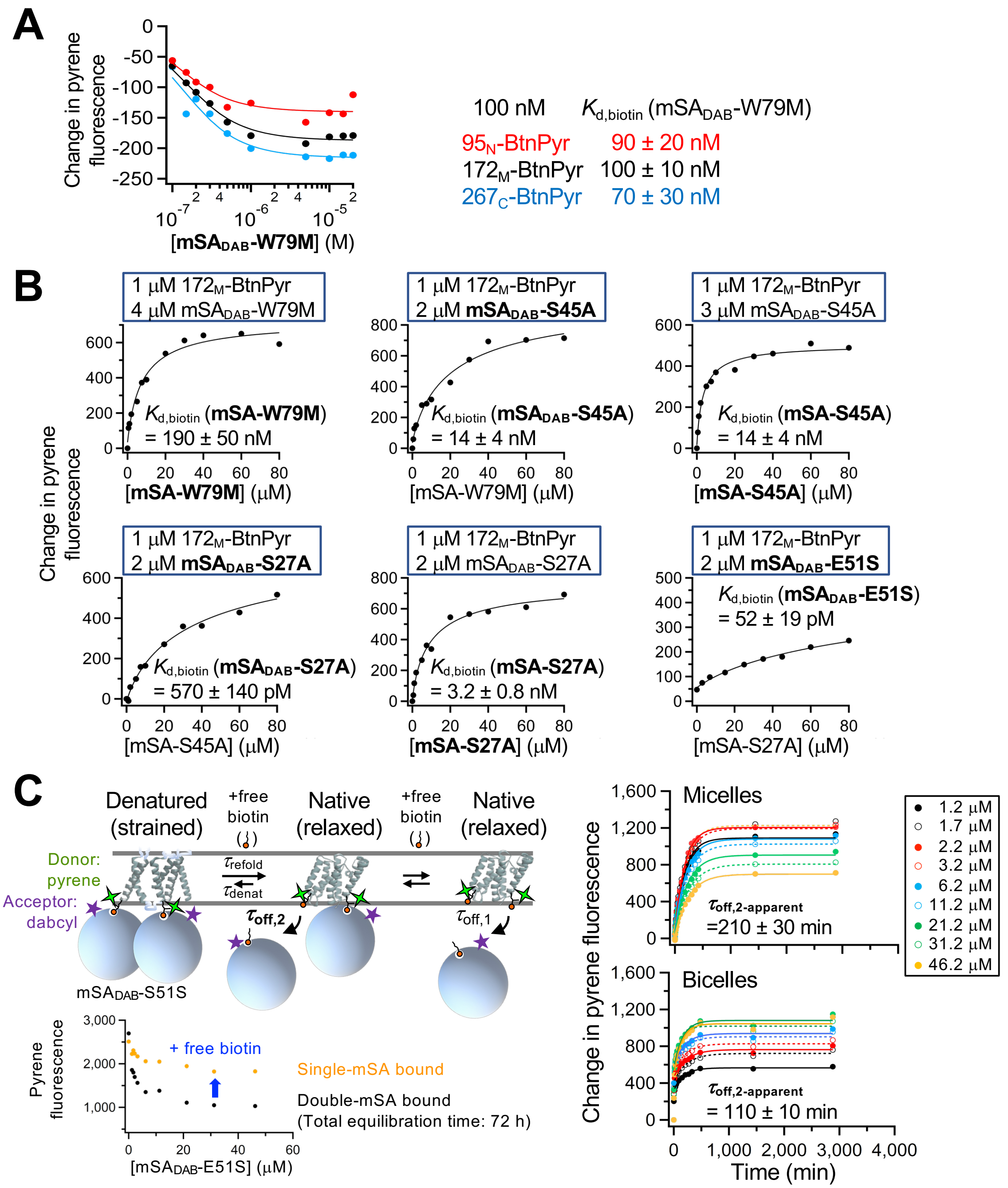

Fig. S4. Binding affinities of mSA variants to BtnPyr on GlpG. **(A)** Binding isotherms between the weakly binding variant mSA-W79M labeled with the dabcyl quencher (mSA_{DAB}-W79M) and GlpG variants with a single biotin label located at three different positions. Errors denote means ± SD from fitting. **(B)** Competition assay results to determine $K_{d,biotin}$'s between higher-affinity mSA variants and a single-biotin variant of GlpG (172_M-BtnPyr). Errors denote means ± SD from fitting. **(C)** Measuring the lifetime of the second bound mSA_{DAB}-S51S ($\tau_{off,2}$) on GlpG using dequenching of pyrene fluorescence upon addition of excess free biotin (2 mM). Errors denote means ± SD from fitting.

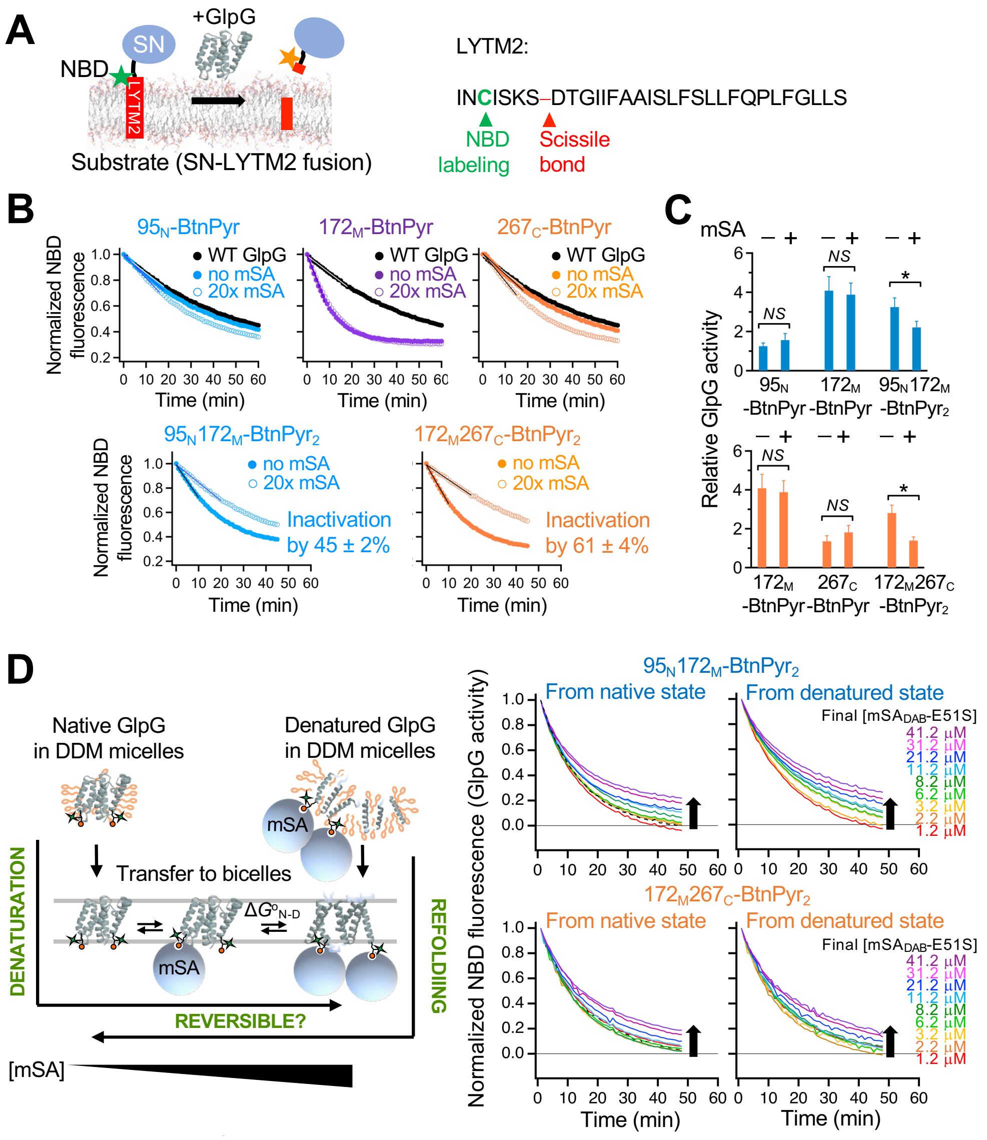

Fig. S5. Activity assays to monitor GlpG denaturation via steric trapping. (A) Description of an assay to measure the proteolytic activity of GlpG using the TM model substrate LYTM2 (LYTM2: the second TM segment of the *E. coli* lactose permease; SN: staphylococcal nuclease fusion). The cysteine residue five-residue upstream from the scissile bond was labeled with the environment-sensitive NBD fluorophore. The cleavage of LYTM2 by GlpG induces quenching of NBD fluorescence. **(B, C)** Inactivation (i.e., denaturation) occurs when the binding of mSA to the doubly biotinylated variants of GlpG is saturated, not when mSA binds to the individual singly biotinylated variants. The *p*-values were obtained from two-tailed, pairwise student's *t*-tests (*NS*: $p>0.05$; *: $p<0.05$). This test was chosen because there was no assumption for the direction of changes in fluorescence signal from means. The observed incomplete inactivation is due to the incomplete double labeling of biotin (**fig. S6C**). Errors denote ± SEM ($N = 3$ biological replicates). (**D**) (*Left*) Testing the reversibility of GlpG folding. Refolding and denaturation of GlpG were monitored by GlpG activity with increasing concentrations of mSA_{DAB}-S51S in bicelles. (*Right*) The assay results.

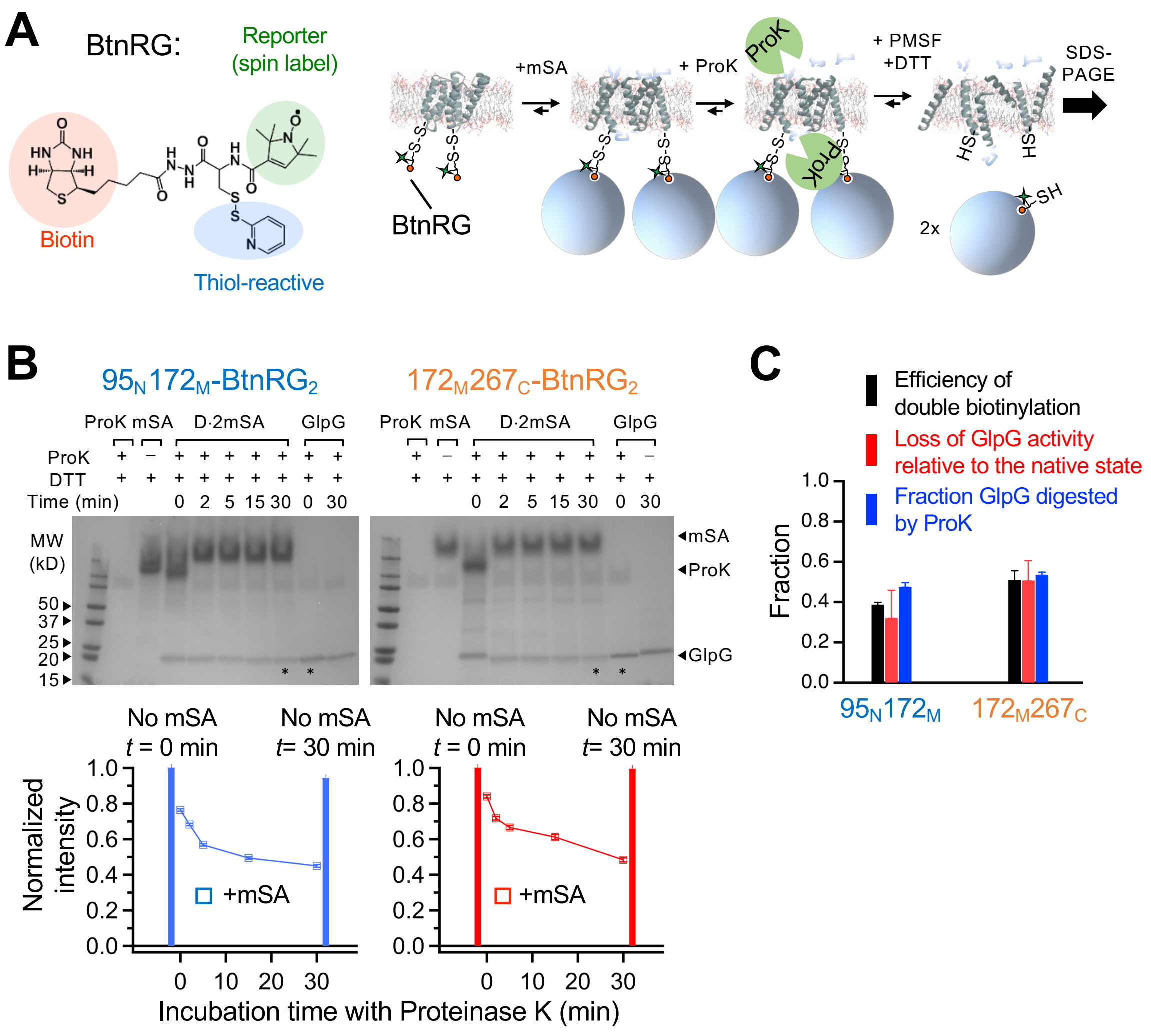

Fig. S6. Denaturation of GlpG induced by steric trapping monitored by proteolysis. (A) (*Left*) Structure of the thiol-reactive biotin label BtnRG-TP. The thiopyridine (TP) group reacts with the thiol group in the cysteine residue to form a disulfide linkage between GlpG and biotin. (*Right*) Strategy to detect sterically denatured GlpG through proteolysis by Proteinase K (ProK). GlpG doubly labeled with BtnRG is denatured via steric trapping using mSA-WT. ProK would cleave the unfolded and solvent-accessible regions in denatured GlpG as the incubation time with ProK increases. At each incubation time, the protease inhibitor PMSF and the reducing agent DTT were successively added to inactivate ProK and to break the disulfide linkages between GlpG and the biotin labels with bound mSA, respectively. The final products were analyzed by SDS-PAGE. **(B)** (*Top*) Selective digestion of sterically denatured GlpG monitored by SDS-PAGE with increasing incubation times with ProK. (*Bottom*) The band intensities that correspond to GlpG on SDS-PAGE gels were analyzed using the ImageJ program. As controls, the intensities of GlpG without mSA (i.e., native GlpG) at the times 0 min and 30 min with or without ProK are shown. **(C)** Correlation between the efficiency of double biotinylation, the activity loss induced by steric trapping, and digestion by ProK (all in the fraction of GlpG). Incomplete digestion in the presence of excess mSA is due to the incomplete double-biotin labeling of GlpG. Error bars denote ± SEM ($N = 3$ biological replicates).

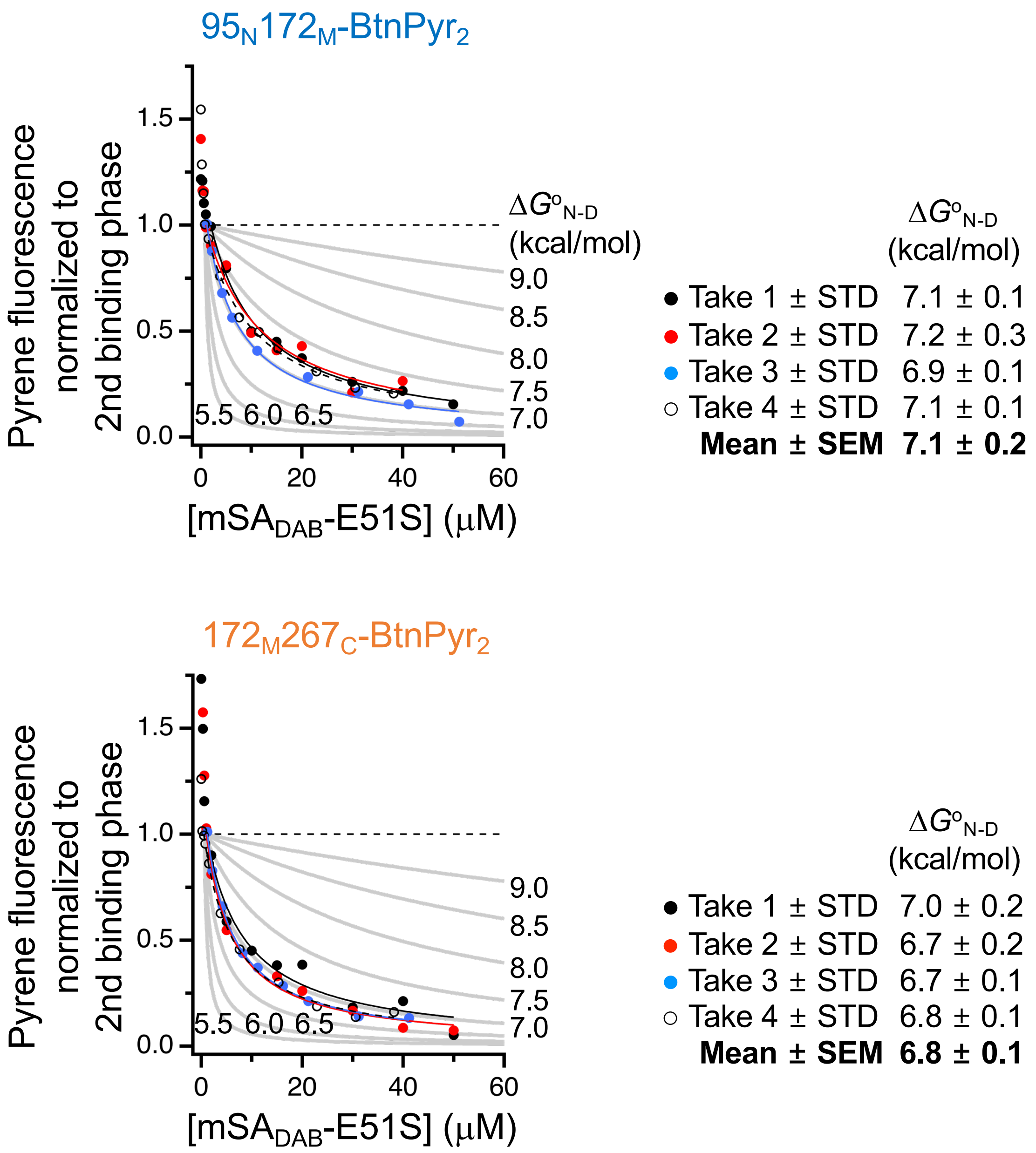

Fig. S7. Precision of stability measurements using steric trapping. Binding isotherms from four independent stability measurements (i.e., $N = 4$ biological replicates) in 3 w/v-% DMPC:CHAPS bicelles ($q = 1.5$) at 24.5 ± 0.5 °C. The doubly biotinylated variant of 1 μM GlpG (95_N172_M–$BtnPyr_2$ or 172_M267_C–$BtnPyr_2$) was titrated with increasing concentrations of mSA_{DAB}-E51S. Binding was monitored with pyrene fluorescence. In each isotherm, fluorescence intensities were normalized with the amplitude of the attenuated second binding phase.

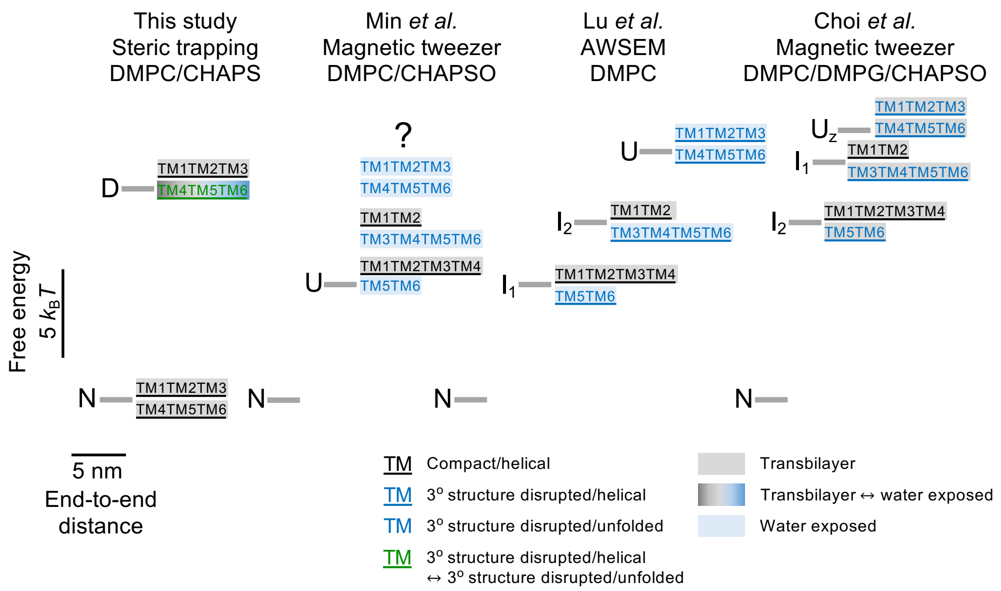

Fig. S8. Comparison of the folding free energy landscapes of GlpG in the bilayer measured with various methods. The positions of the free energy level (in k_BT) and the degree of compactness (the end-to-end distance between the N- and C-termini) of each state are scaled relative to the native state ("N"). The conformational features of each state were described regarding the tertiary interaction, ("compact" vs "3° structure disrupted"), the secondary structure ("helical" *vs* "unfolded"), or burial in the membrane ("transbilayer" *vs* "exposed to water"). "D": the denatured state; "I": the intermediate state; "U": the unfolded state. The stability of GlpG determined directly under native conditions ($\Delta G^o_{N\text{-}D}$ = ~–12k_BT, "This study") in bicelles was larger than the stability value in the same neutral bicelles in a single-molecule magnetic tweezer study (–6.5k_BT, "Min *et al.*") (*65*). In the latter, $\Delta G^o_{N\text{-}D}$ is obtained by extrapolating the unfolding and refolding rates measured in two distinct force ranges (12 pN to 30 pN for unfolding and 2 pN to 7 pN for refolding) to zero force. In the higher force range, GlpG unfolds via a single cooperative step or via multiple steps involving one or two intermediates to a fully stretched coil (*65*). In the lower force range, the conformation of the starting unfolded state prior to refolding has not been defined ("?"). A computational study ("Lu et al. AWSEM") predicts that the unfolded state at low force is I_1 (TM1–TM4 folded) (*66*). Notably, our $\Delta G^o_{N\text{-}D}$ value is similar to the free energy difference between the native state and I_2 (TM1–TM2 folded) from the same simulation (–10k_BT) (*66*) as well as to the free energy difference between the native state and I_1 (TM1–TM2 folded) from a more recent magnetic tweezer study in the negatively charged bicelles (–13k_BT) at low force ("Choi et al.") (*67*). The end-to-end distances for "N" and "D" states in "This study" were taken from our previous work measured in the negatively charged bicelles (DMPC:DMPG:CHAPS) using DEER (*10*). In that study, we have shown that while both N- (TM1–TM3) and C- (TM4–TM6) subdomains in sterically denatured GlpG are expanded relative to the native state (*10*), the compactness of N-subdomain is close to the collapse limit and that of C-subdomain is close to the full-expansion limit, resembling the conformational ensemble of I_2 from "Lu et al." (*66*) and of I_1 from "Choi et al." (*67*). Thus, the discrepancy in stability is likely due to the differences in conformation between the denatured states in the steric trapping ("This study") and magnetic tweezer ("Min et al.") studies.

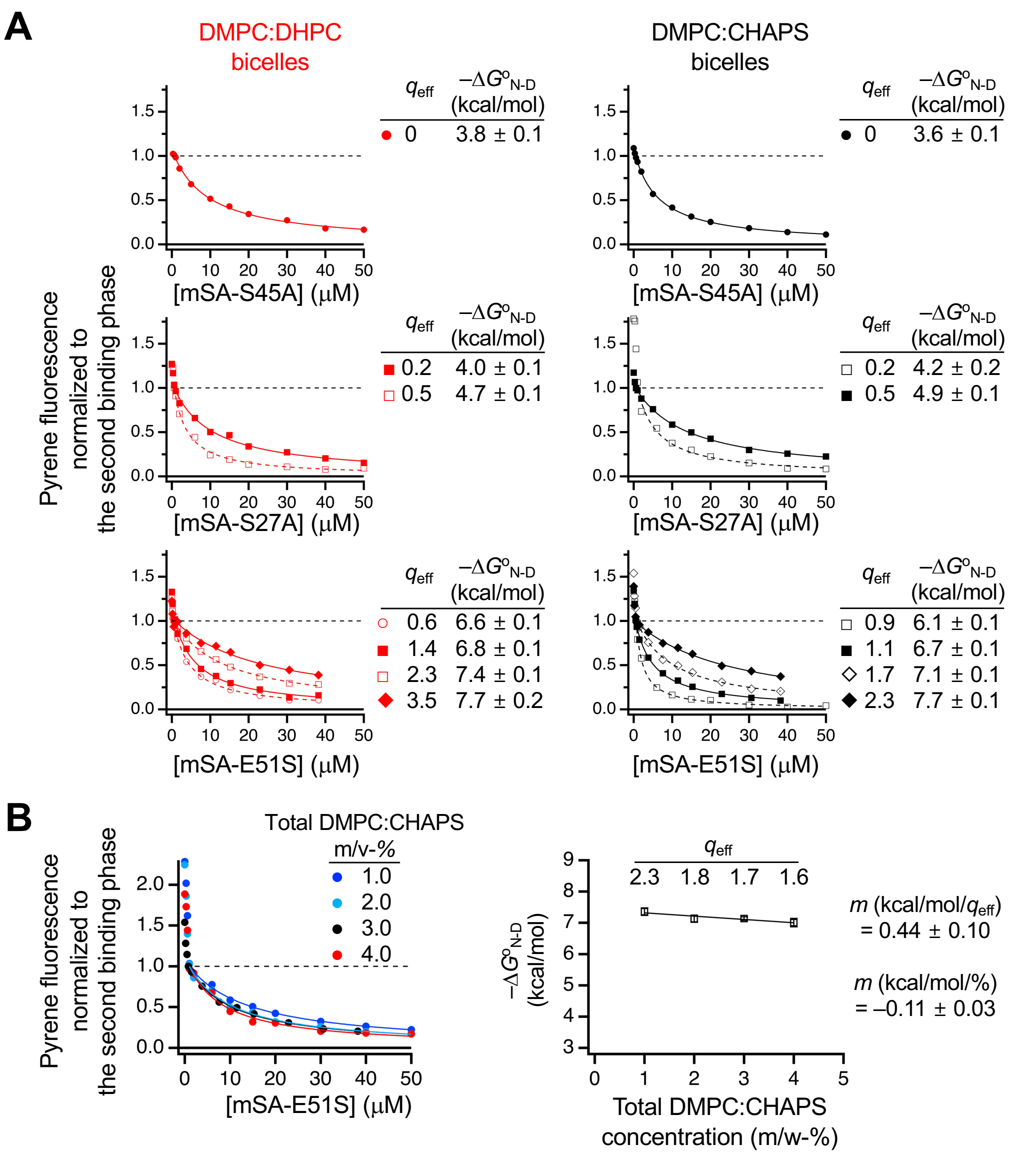

Fig. S9. GlpG stability with increasing lipid contents in bicelles. q_{eff} values were calculated using the fixed free detergent concentrations ([DHPC]$_{free}$ = 6.5 mM and [CHAPS]$_{free}$ = 2.5 mM) (**Eq. 8**). **(A)** Binding isotherm data for obtaining the $\Delta G^o_{N\text{-}D}$ of GlpG (95_N172_M–$BtnPyr_2$) at various q_{eff}-values in the two types of bicelles (DMPC:DHPC-*left* and DMPC:CHAPS-*right*). The total amphiphilic concentration was fixed to 3 w/v-% and the molar ratio of lipid to detergent (i.e., *q*-value) was varied to yield desired q_{eff} values. Errors denote means ± SD from fitting. **(B)** (*Left*) Binding isotherms for obtaining the $\Delta G^o_{N\text{-}D}$ of GlpG (95_N172_M–$BtnPyr_2$) at various total amphiphile concentrations (DMPC and CHAPS) at a fixed *q*-value = 1.5. (*Right*) The dependence of GlpG stability ($-\Delta G^o_{N\text{-}D}$) on the total amphiphile concentration (DMPC and CHAPS) at a fixed *q*-value = 1.5. The "*m*" represents the fitted linear slope of $-\Delta G^o_{N\text{-}D}$ as a function of q_{eff} or w/v-%. Errors denote means ± SD from fitting.

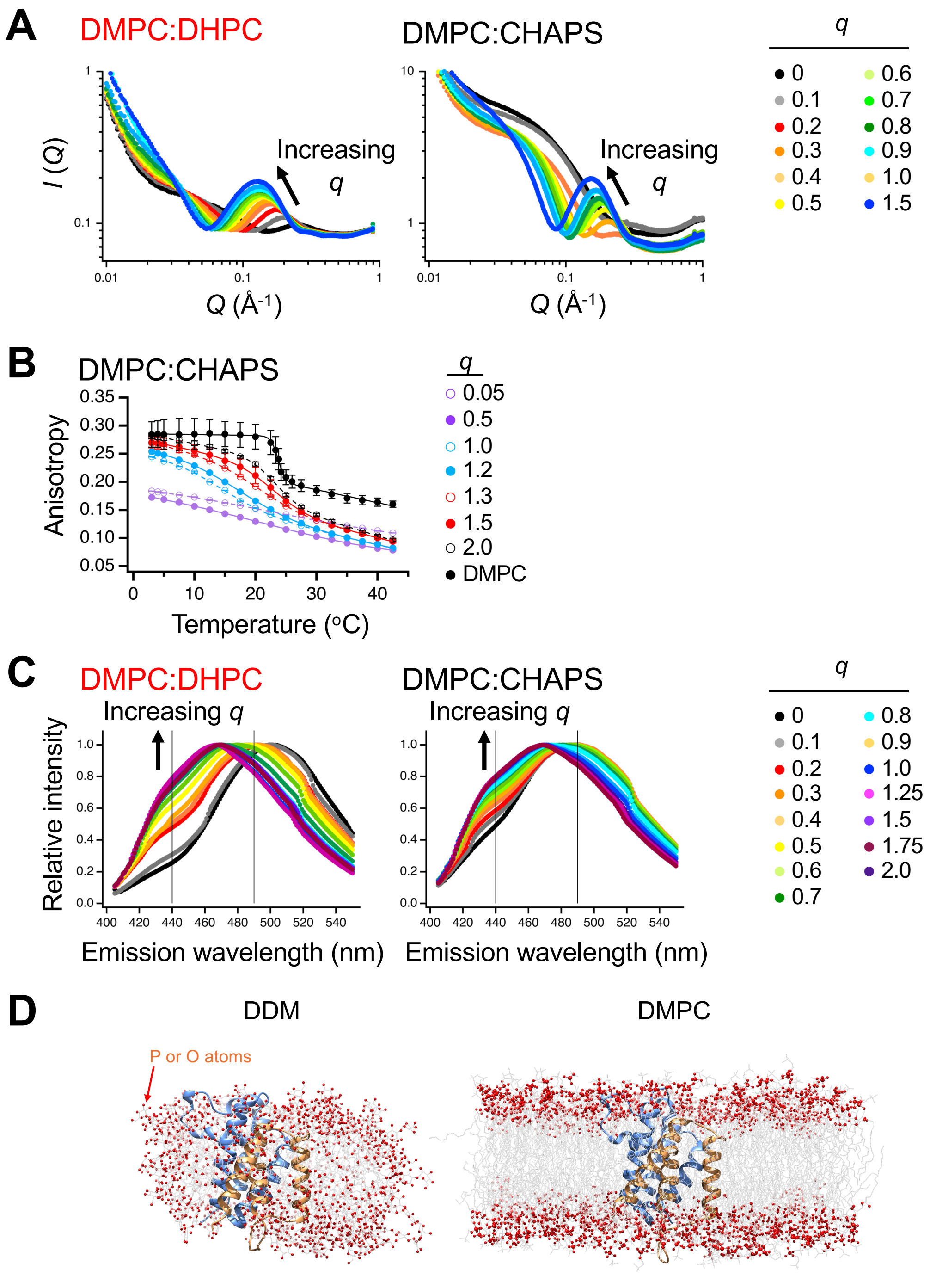

Fig. S10. Characterization of bicelles using SAXS, fluorescence anisotropy, and generalized polarization. (A) SAXS scattering profiles for DMPC:DHPC and DMPC:CHAPS bicelles. **(B)** Temperature-dependent fluorescence anisotropy of DPH incorporated into DMPC:CHAPS bicelles and large unilamellar DMPC liposomes. **(C)** Fluorescence spectra of Laurdan incorporated in DMPC:DHPC and DMPC:CHAPS bicelles. The vertical lines indicate the emission wavelengths of 440 nm and 490 nm, whose intensities were used for calculating the generalized polarization (*GP*, **Eq. 7**). **(D)** Structural snapshots from MD simulations of the GlpG–DDM micelle and GlpG–DMPC bilayer complexes after 2 μs of product runs.

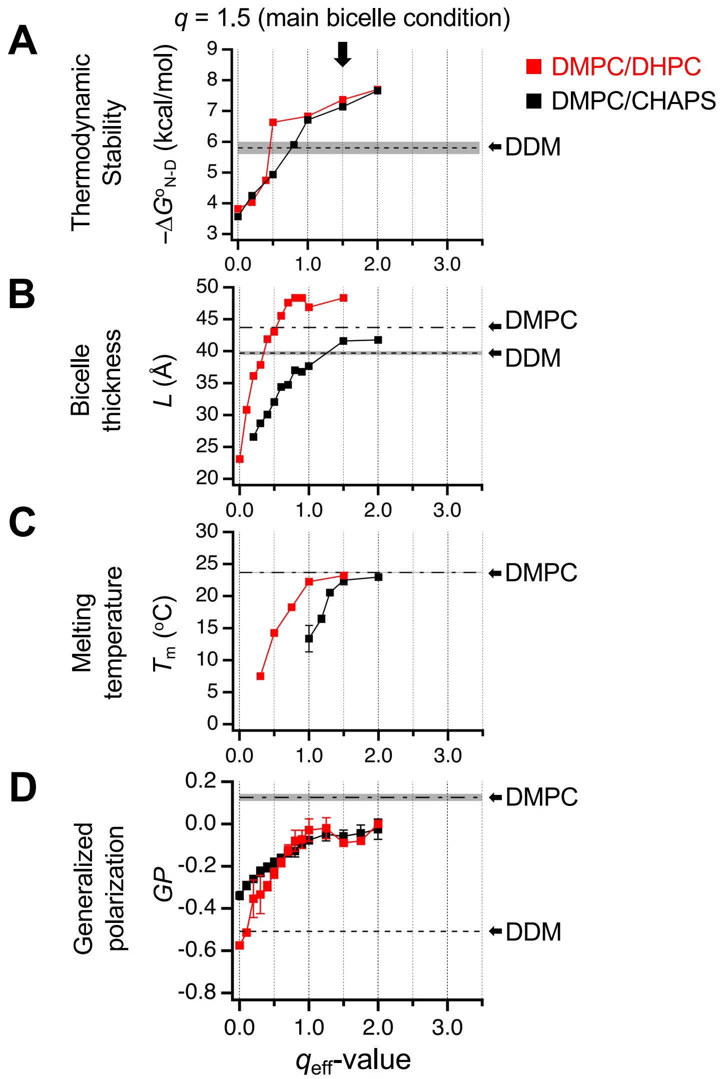

Fig. S11. GlpG stability and physical parameters of bicelles with increasing lipid contents (q_{eff}) in DMPC:CHAPS and DMPC:DHPC bicelles. Here, q_{eff} was calculated using **Eq. 9** assuming that lipid and detergents are ideally mixed. **(A)** Thermodynamic stability ($-\Delta G^{o}_{N\text{-}D}$) of the doubly biotinylated variant of GlpG, $95_{N}172_{M}$–BtnPyr$_2$. Errors denote means ± SD from fitting. **(B)** Disk thickness (L) measured by SAXS. Thickness of DDM micelles was adapted from Ref. (*64*). **(C)** The gel–fluid phase transition temperature (T_m) determined by fluorescence anisotropy of DPH incorporated in bicelles and DMPC liposomes. The data for DHPC:DMPC bicelles was adapted from Ref. (*54*). Errors denote ± SEM (N = 3 biological replicates). **(D)** Generalized polarization (GP) of Laurdan fluorescence for determining the amphiphile–amphiphile packing strength. Errors denote ± SEM (N = 3 biological replicates).

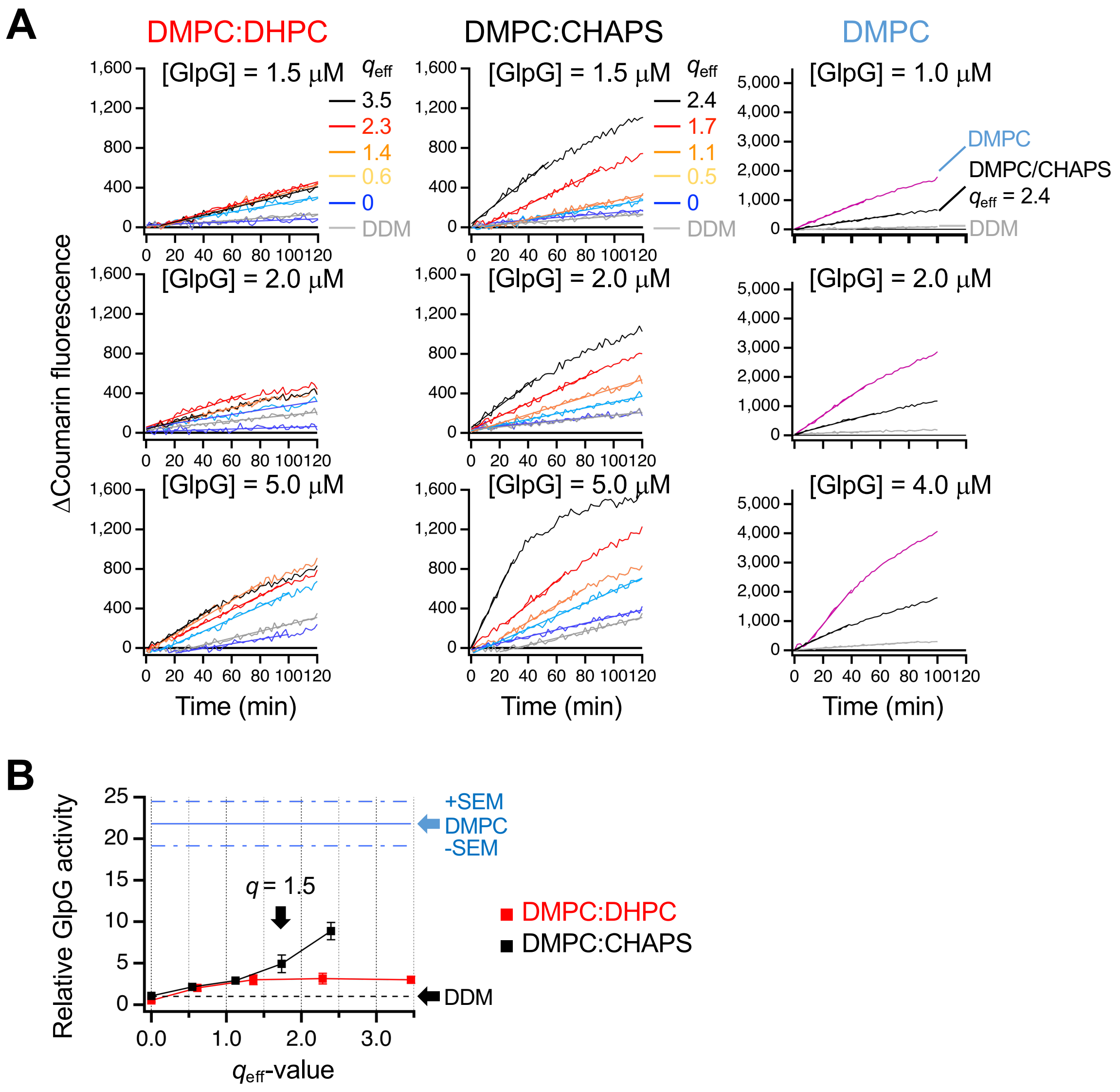

Fig. S12. GlpG activity with increasing lipid contents in bicelles. (A) Proteolytic activity of GlpG (95_N172_M–BtnPyr$_2$) measured as a function of q_{eff} in the two types of bicelles (DMPC:CHAPS and DMPC:DHPC; 3 w/v-%). Here, q_{eff} was calculated using **Eq. 8**. An internally quenched water-soluble peptide, mca-RPKPYAv/WM-K(dnp), was used as a model substrate (mca: 7-methoxycoumarin; dnp: dinitrophenol; v: norvaline; "/": the scissile peptide bond). GlpG activity was also measured in DDM (3 w/v-%) micelles and DMPC (3 w/v-%) liposomes as references. **(B)** Activity in bicelles is represented as the relative activity to that in DDM micelles. The q_{eff}-value of primary bicelles in this study (q = 1.5) is marked with a block arrow. The relative activity of GlpG reconstituted in pure DMPC liposomes is shown as a horizontal reference line. Errors denote ± SEM (N = 3 biological replicates).

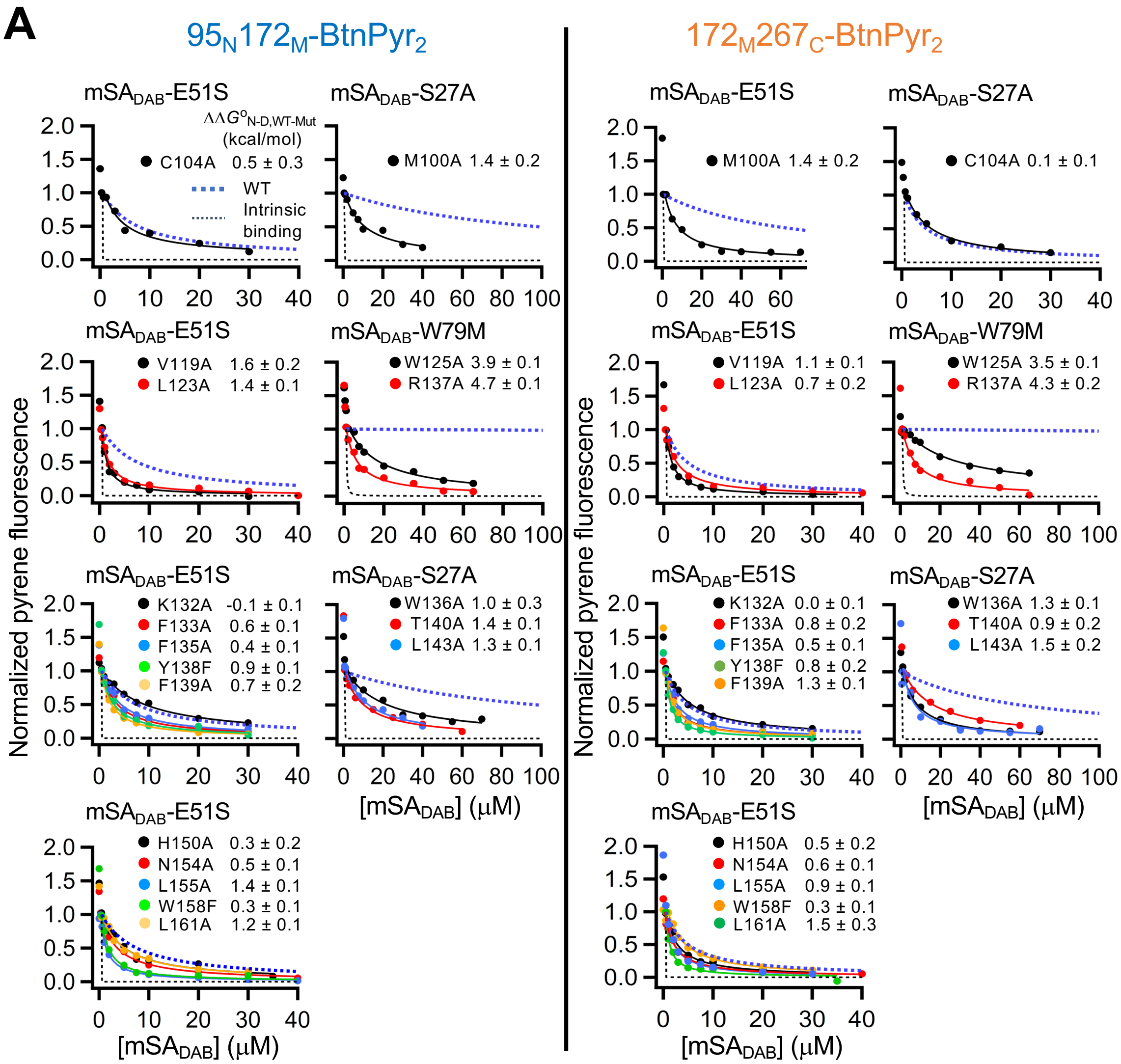

Fig. S13. Binding isotherms between mSA and the doubly biotinylated variant of GlpG WT and mutants to determine $\Delta G^{o}_{N\text{-}D}$ in DMPC:CHAPS bicelles using steric trapping. Binding was measured using quenching of pyrene fluorescence from the BtnPyr labels on GlpG by the dabcyl quencher conjugated to mSA (mSA_{DAB}). In each plot, fluorescence intensities were normalized to the total intensity change of the second binding phase. For a given mSA variant (shown at the top of each plot), the binding data with WT GlpG and the predicted unhindered binding (i.e., the intrinsic binding of mSA variant to the biotin labels) are shown as blue and black dashed lines, respectively. Stability differences between WT and mutant GlpG ($\Delta\Delta G^{o}_{N\text{-}D,WT\text{-}Mut} = \Delta G^{o}_{N\text{-}D,WT} - \Delta G^{o}_{N\text{-}D,Mut}$) are shown. For a given GlpG mutant, an optimal mSA variant was selected based on the criteria that the attenuated second binding phase is extended in the [mSA_{DAB}] range up to 40 μM or 80 μM. For a given mSA variant, *the more attenuated second binding phase indicates the higher stability of the protein.* **(A)** Binding isotherms for the GlpG variants bearing a mutation on the segments TM1, L1, and TM2 in the protein. Errors denote means ± SD from fitting. (Continued in the next page)

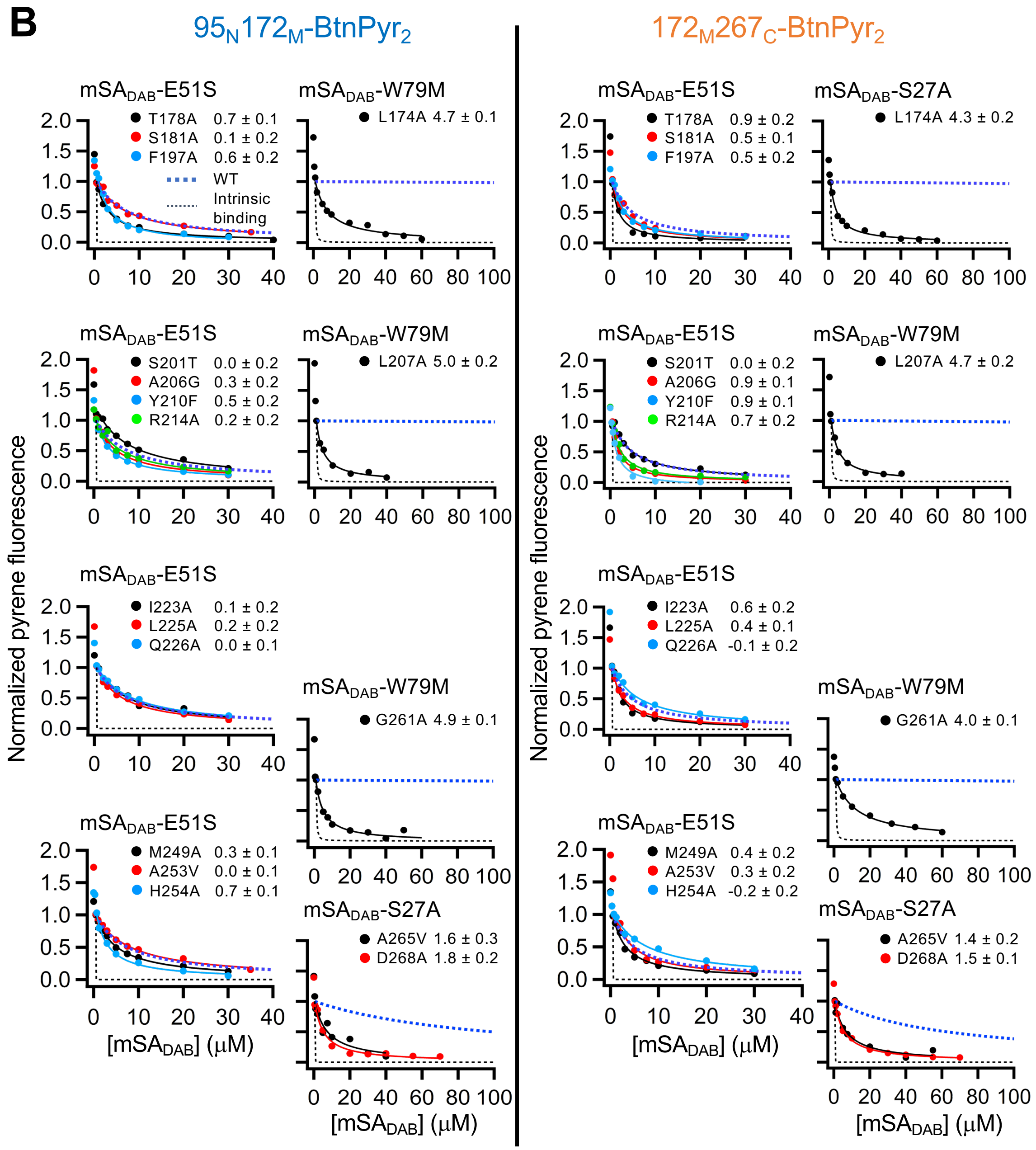

Fig. S13. (Continued from the previous page) **(B)** Binding isotherms for the GlpG variants bearing a mutation on the segments TM3, TM4, TM5 and TM6 in the protein. Errors denote means ± SD from fitting.

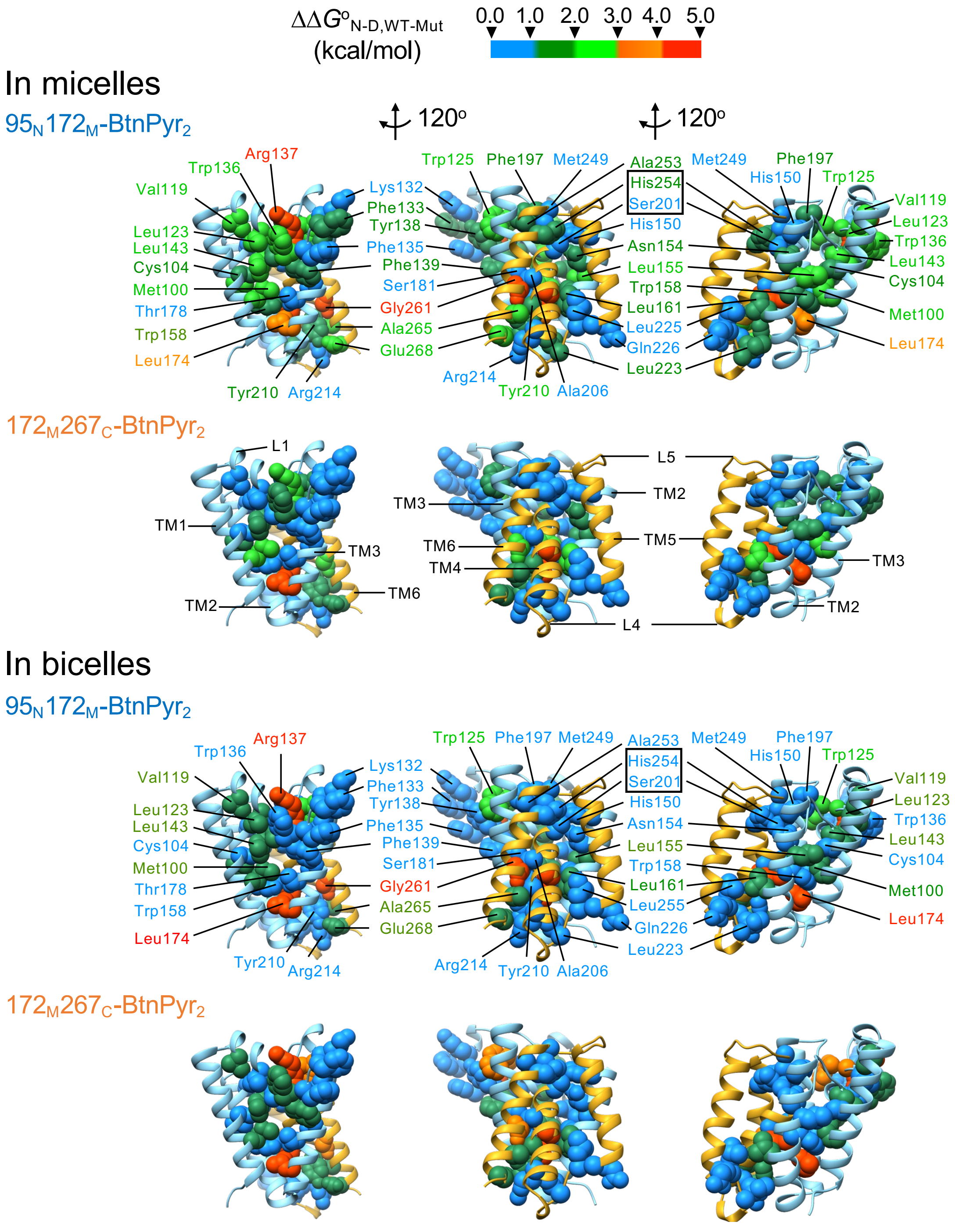

Fig. S14. Mapping of mutation-induced stability changes onto the GlpG structure. Mutation-induced stability changes ($\Delta\Delta G^{o}_{\text{N-D,WT-Mut}} = \Delta G^{o}_{\text{N-D,WT}} - \Delta G^{o}_{\text{N-D,Mut}}$) in N and C subdomains of GlpG measured with the doubly biotinylated variants, $95_{N}172_{M}$-BtnPyr$_2$ and $172_{M}267_{C}$-BtnPyr$_2$, respectively, in micelles and bicelles. Under each condition, $\Delta\Delta G^{o}_{\text{N-D,WT-Mut}}$ values are color-coded (*Top*) as a heat map on the structure.

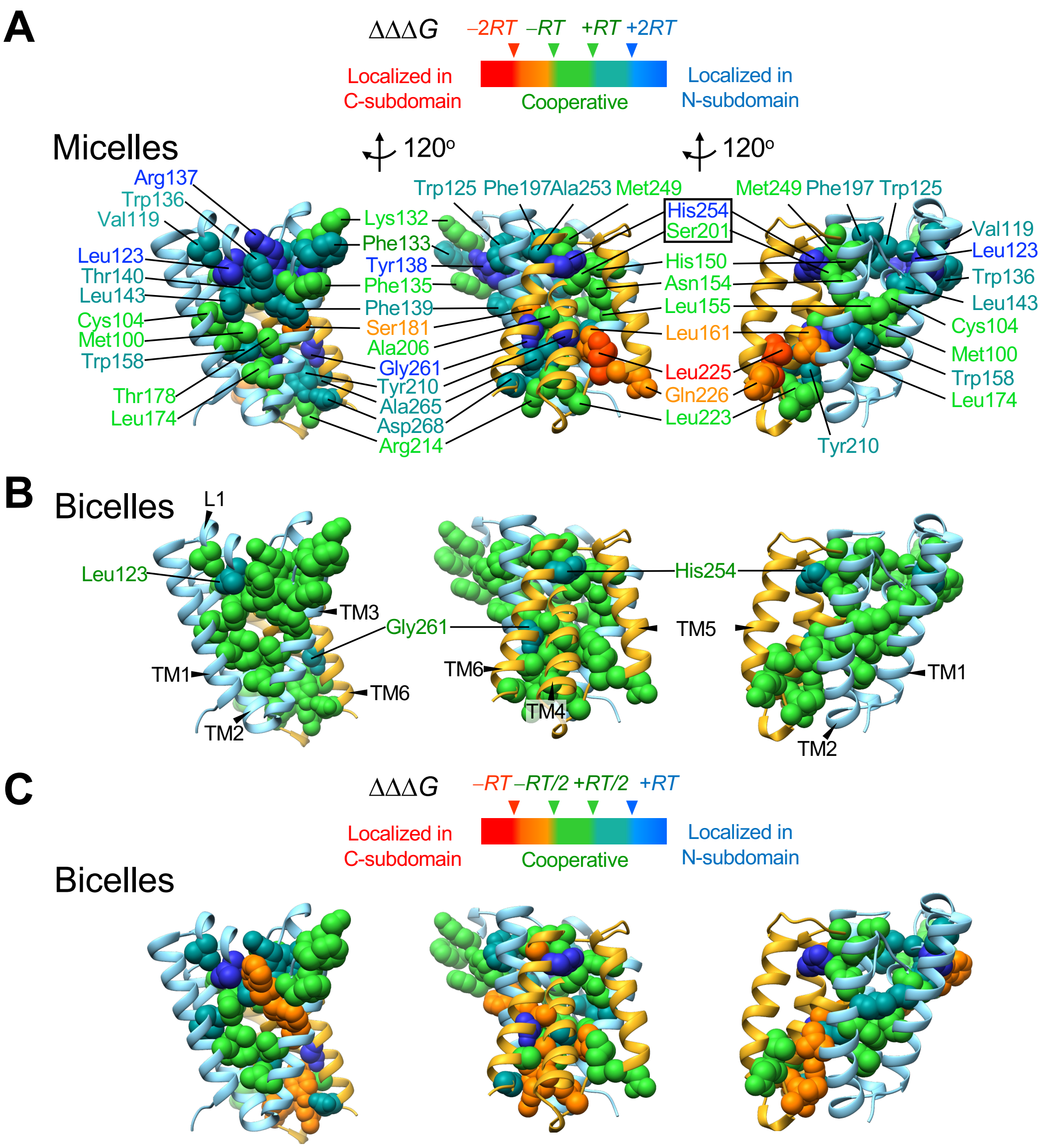

Fig. S15. Cooperativity profiles in micelles are partially conserved in bicelles. (A, B) Comparison of the cooperativity profiles between micelles **(A)** and bicelles **(B)** based on the standard cut-off values, –2*RT*, –*RT*, +*RT*, and +2*RT* (i.e., the *RT* scale, where *R*: gas constant; *T*: absolute temperature). **(C)** Cooperativity profiles in bicelles using narrowly spaced cut-off values, –*RT*, –1/2·*RT*, +1/2·*RT*, and +*RT* (i.e., the 1/2·*RT* scale). Except for several residues (Phe135, Phe136, Ala203, Ala206, Leu225, Gln226, and Arg214), the profiles reconstructed using the 1/2·*RT* scale in bicelles resemble those using the *RT* scale in micelles. The preserved features include: the cooperative packing core (formed by TM1, TM2, and TM3), the cooperative cluster in the active site (Ser201, His150, and Asn154), the localized cluster in L1, and the overpropagated cluster at the TM4–TM6 interface.

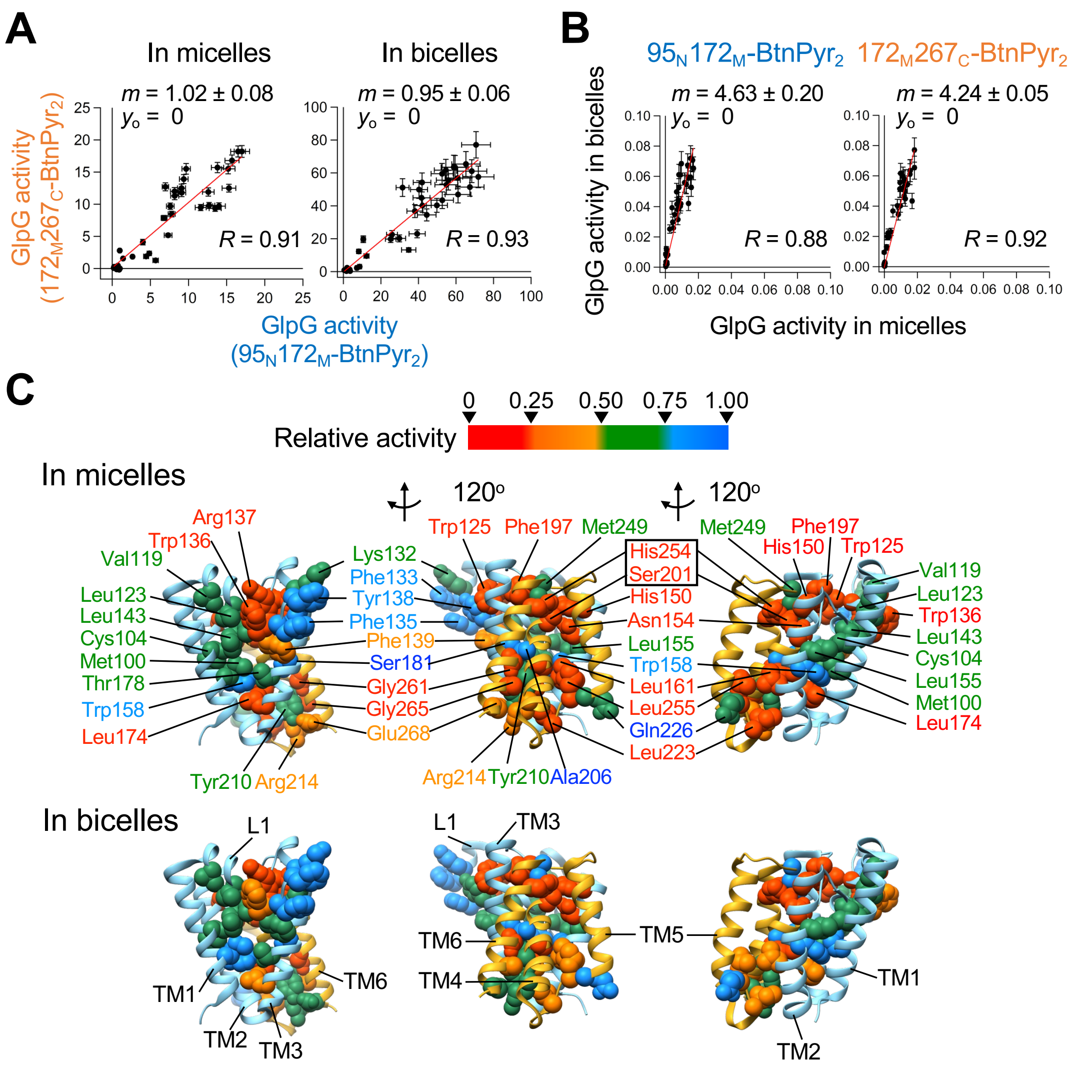

Fig. S16. Proteolytic activity of GlpG WT and mutants for the TM model substrate LYTM2. (A) The effect of the position of the biotin pair (i.e., 95_N172_M-BtnPyr$_2$ *vs* 172_M267_C-BtnPyr$_2$) on GlpG activity. In both micelles and bicelles, the correlation slopes (m) are close to unity, indicating that the location of the biotin pair does not affect GlpG activity. Activity values in **tables S2 and S3** were used. Errors denote ± SEM (N = 3 biological replicates). **(B)** The effect of the hydrophobic environment (i.e., micelles *vs* bicelles) on GlpG activity. All activity values correspond to the fractional substrate turnover rate (min^{-1}) normalized to the initial substrate concentration (10 μM) in DDM micelles (5 mM) or DMPC:CHAPS bicelles (3 w/v-%, q = 1.5) as measured by NBD fluorescence (**fig. S5**). Errors denote ± SEM (N = 3 biological replicates). **(C)** Mapping of mutation-induced activity changes onto the structure of GlpG. The activities of each mutant measured in the backgrounds of 95_N172_M-BtnPyr$_2$ and 172_M267_C-BtnPyr$_2$ were normalized to the corresponding activities of WT (95_N172_M-BtnPyr$_2$ and 172_M267_C-BtnPyr$_2$, respectively, without additional mutation), and then averaged for structural mapping.

A

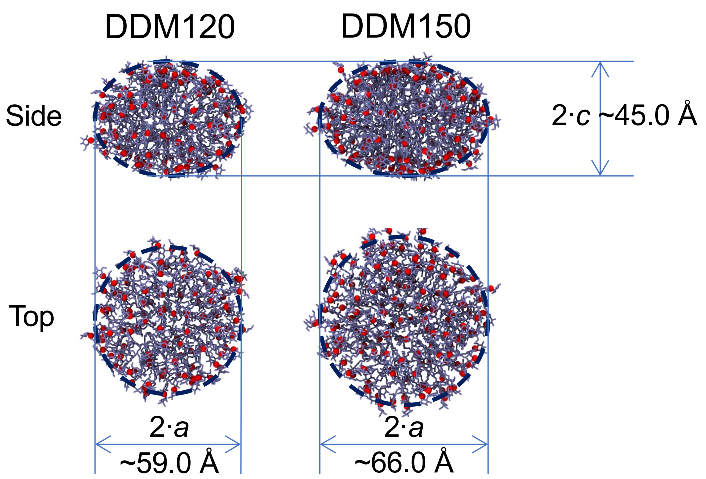

B

Oblate structure	DDM120	DDM150	SAXS ($N_{A,DDM}$ = 135-149)
Radius, *a*	29.5 ± 0.8 Å	32.9 ± 0.8 Å	33.7 ± 0.4 Å
Radius, *c*	22.6 ± 1.1 Å	22.5 ± 0.9 Å	21.5 ± 0.4 Å
Oblateness	0.23 ± 0.06	0.31 ± 0.04	0.36 ± 0.01
Area per DDM	77.3 ± 1.6 Å^2	72.1 ± 1.7 Å^2	76.3 ± 4.0 Å^2

Fig. S17. Modelling of micelles for MD simulation. (A) Two micellar systems in this study, one with 120 DDM molecules (DDM120) and the other with 150 DDM molecules (DDM150). The shapes of both micellar systems were approximated to oblate spheroids as described by the equation below:

$$\frac{x^2}{a^2} + \frac{y^2}{a^2} + \frac{z^2}{c^2} = 1$$

, where *x*, *y*, and *z* are the cartesian coordinates in the 3D-space. The semi-axes *a* and *c* are aligned along each symmetry axis, each indicating the equatorial radius in the *xy*-plane and the distance from the spheroid center to the pole along the symmetry axis of *z*. $a>c$ forms an oblate spheroid, while $a<c$ corresponds to a prolate spheroid. All coordinates of DDM 2O4 atoms in a micelle were used to describe the spheroidal shape of the micelle, which were then subjected to a parametric fitting for obtaining the semi-axes, *a* and *c*. We found that both DDM120 and DDM150 create oblate spheroidal shapes (i.e., $a>c$), from which the effective cross-sectional area per DDM molecule was evaluated using the equation, $A_{oblate} = 2pa^2 + pc^2/e \cdot \ln[(1 + e)/(1 - e)]$ (the eccentricity, *e*, is defined as $e = [1 - c^2/a^2]^{1/2}$) (*114*). **(B)** As the number of DDM molecules increases from 120 to 150, the equatorial dimension increases from *a* = 29.5 Å to 32.9 Å while the axial dimension ($2 \cdot c$) remained nearly constant at *c* = ~22.5 Å. The area per DDM at the micellar surface is larger in DDM120 providing room for each DDM molecule to relax fast in the micelles compared to that in DDM150. The experimental values obtained from small-angle X-ray scattering (SAXS) are also shown for comparison (*64*). In the SAXS data, the *a* and *c* values are obtained by adding the core radius and shell thickness based on the two-component core-shell models (*64*).

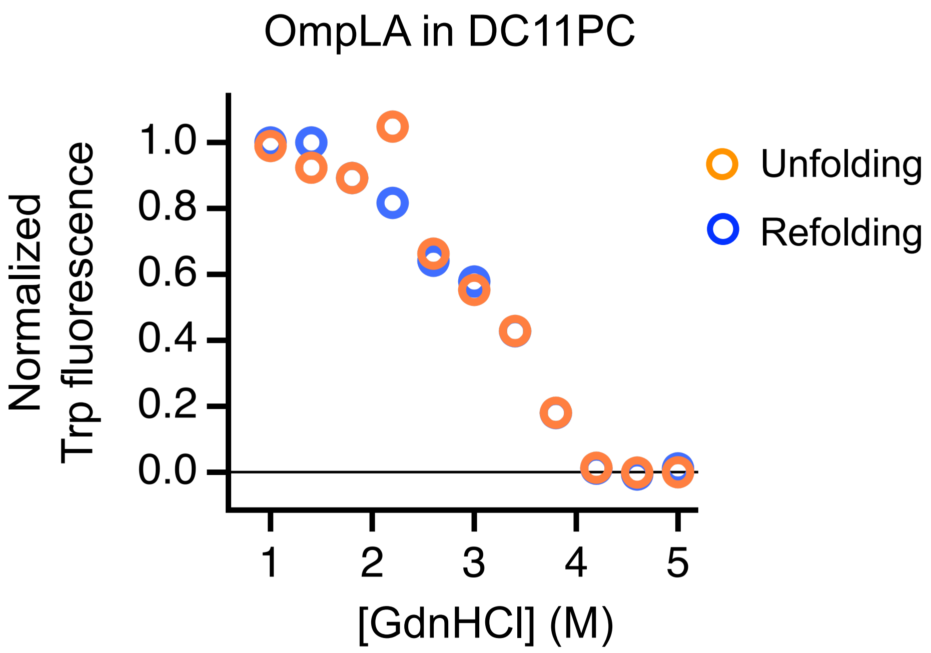

Fig. S18. The reversibility of OmpLA folding in DC11PC bilayers. The unfolding and folding titrations of OmpLA show no hysteresis indicating the reversibility of folding.

Bicelles (DMPC:CHAPS, q = 1.5)							
	r_{Prob} (Å)	r_{Median} (Å)	r_{Mean} (Å)	σ_{Mean} (Å)	χ^2	Modulation depth	Distance limit (Å)
95_N172_M	24.4	28.2	32.7	12.2	1.15	0.352	59
$95_N172_M\cdot mSA_2$	56.9	42.4	37.5	14.9	1.30	0.063	57
172_M267_C	24.4	29.4	33.8	14.5	1.18	0.250	60
$172_M267_C\cdot mSA_2$	55.6	48.4	41.2	14.9	1.31	0.208	60

Micelles (DDM)*							
	r_{Prob} (Å)	r_{Median} (Å)	r_{Mean} (Å)	σ_{Mean} (Å)	χ^2	Modulation depth	Distance limit (Å)
95_N172_M	26.5	27.2	28.7	5.9	1.45	0.015	54
$95_N172_M\cdot mSA_2$	53.4	47.4	43.6	12.4	1.43	0.008	54
172_M267_C	27.8	25.2	25.2	4.1	3.19	0.032	54
$172_M267_C\cdot mSA_2$	53.0	52.6	50.9	7.5	1.72	0.019	54

*The data in micelles was adapted from Ref. (*60*).

Table S1. Fitted parameters of DEER data for native and sterically denatured GlpG in bicelles and micelles. The background-subtracted dipolar evolution data was fitted with the model-free, non-negative regularization. r_{Prob}: the most probable distance; r_{Med}: the median distance; r_{Mean}: the mean distance; σ_{Mean}: the standard deviation of the mean. χ^2 indicates the goodness of fit. Modulation depth, which ranges from 0 to 1, represents the fraction of interacting spin pairs that lie within the detectable distance limit of the experiment. For a given DEER experiment, distance limit is the interspin distance, below which modeled distances can be determined with confidence. Distance limits depend on variations in DEER experimental parameters, sample quality, and amount.

Secondary structure	Mutation	Location	f_{ASA}	N-subdomain (95_N172_M)		C-subdomain (172_M267_C)		$\Delta\Delta\Delta G^o$	Cooperativity profile
				$\Delta\Delta G^o_{WT-Mut}$	Rel.Activity	$\Delta\Delta G^o_{WT-Mut}$	Rel.Activity		
TM1	M100A	N	0.14	1.4 ± 0.2	0.86 ± 0.09	1.4 ± 0.2	0.74 ± 0.08	0.0 ± 0.2	Cooperative
	C104A	N	0	0.5 ± 0.3	0.84 ± 0.09	0.1 ± 0.4	0.58 ± 0.06	0.4 ± 0.4	Cooperative
L1	V119A	N	0.05	1.6 ± 0.2	0.59 ± 0.07	1.1 ± 0.1	0.70 ± 0.07	0.5 ± 0.2	Cooperative
	L123A	N	0.23	1.4 ± 0.1	0.57 ± 0.06	0.7 ± 0.2	0.65 ± 0.07	0.7 ± 0.2	Moderate/N
	W125A	N	0.09	3.9 ± 0.1	0.17 ± 0.02	3.5 ± 0.1	0.12 ± 0.01	0.5 ± 0.1	Cooperative
	K132A	N	0.56	-0.1 ± 0.1	0.79 ± 0.09	0.0 ± 0.1	0.72 ± 0.08	-0.1 ± 0.2	Cooperative
	F133A	N	0.80	0.6 ± 0.1	0.83 ± 0.10	0.8 ± 0.2	0.82 ± 0.09	-0.2 ± 0.2	Cooperative
	F135A	N	0.73	0.4 ± 0.1	1.02 ± 0.12	0.5 ± 0.1	0.75 ± 0.09	-0.1 ± 0.2	Cooperative
	W136A	N	0.46	1.0 ± 0.3	0.22 ± 0.02	1.3 ± 0.1	0.30 ± 0.03	-0.3 ± 0.3	Cooperative
	R137A	N	0.04	4.7 ± 0.1	0.01 ± 0.01	4.3 ± 0.2	0.01 ± 0.01	0.4 ± 0.2	Cooperative
	Y138F	N	0.25	0.9 ± 0.1	0.75 ± 0.08	0.8 ± 0.2	0.56 ± 0.07	0.1 ± 0.2	Cooperative
	F139A	N	0.38	0.7 ± 0.2	0.63 ± 0.07	1.3 ± 0.1	0.45 ± 0.05	0.5 ± 0.2	Cooperative
	T140A	N	0.20	1.4 ± 0.1	0.59 ± 0.07	0.9 ± 0.2	0.62 ± 0.07	0.5 ± 0.2	Cooperative
	L143A	N	0.25	1.3 ± 0.1	0.78 ± 0.09	1.5 ± 0.2	0.68 ± 0.08	-0.2 ± 0.2	Cooperative
TM2	H150A	N	0.01	0.3 ± 0.2	0.10 ± 0.01	0.5 ± 0.2	0.03 ± 0.00	-0.2 ± 0.3	Cooperative
	N154A	N/Interface	0	0.5 ± 0.1	0.12 ± 0.01	0.6 ± 0.1	0.04 ± 0.09	-0.1 ± 0.1	Cooperative
	L155A	N	0.15	1.4 ± 0.1	0.64 ± 0.07	0.9 ± 0.1	0.79 ± 0.08	0.5 ± 0.2	Cooperative
	W158F	N/Interface	0	0.3 ± 0.1	0.84 ± 0.10	0.3 ± 0.1	0.82 ± 0.09	0.0 ± 0.2	Cooperative
	L161A	N/Interface	0	1.2 ± 0.1	0.36 ± 0.04	1.5 ± 0.3	0.26 ± 0.03	-0.3 ± 0.3	Cooperative
TM3	L174A	N/Interface	0	4.5 ± 0.2	0.49 ± 0.05	4.8 ± 0.1	0.17 ± 0.02	-0.3 ± 0.2	Cooperative
	T178A	N	0.11	0.7 ± 0.1	0.77 ± 0.09	0.9 ± 0.2	0.66 ± 0.07	-0.2 ± 0.2	Cooperative
	S181A	N/Interface	0	0.1 ± 0.2	0.92 ± 0.11	0.5 ± 0.1	0.85 ± 0.09	-0.4 ± 0.2	Cooperative
	F197A	N	0.01	0.6 ± 0.2	0.02 ± 0.00	0.5 ± 0.2	0.00 ± 0.00	0.1 ± 0.2	Cooperative
TM4	S201T	C/Interface	0	0.0 ± 0.2	0.05 ± 0.01	0.0 ± 0.2	0.01 ± 0.00	0.0 ± 0.3	Cooperative
	A206G	C	0	0.3 ± 0.2	1.07 ± 0.12	0.9 ± 0.1	0.92 ± 0.10	-0.6 ± 0.2	Cooperative
	L207A	C/Interface	0	5.0 ± 0.2	0.12 ± 0.01	4.7 ± 0.1	0.16 ± 0.02	0.3 ± 0.2	Cooperative
	Y210F	C	0	0.5 ± 0.2	0.86 ± 0.10	0.9 ± 0.1	0.61 ± 0.07	-0.4 ± 0.2	Cooperative
	R214A	C/Interface	0.10	0.2 ± 0.2	0.70 ± 0.08	0.7 ± 0.2	0.52 ± 0.05	-0.5 ± 0.3	Cooperative
TM5	I223A	C/Interface	0.00	0.1 ± 0.2	0.56 ± 0.06	0.6 ± 0.2	0.30 ± 0.03	-0.6 ± 0.2	Moderate/C
	L225A	C/Interface	0.03	0.2 ± 0.1	0.42 ± 0.05	0.4 ± 0.1	0.26 ± 0.03	-0.3 ± 0.1	Cooperative
	Q226A	C	0.57	0.0 ± 0.1	0.95 ± 0.11	-0.1 ± 0.2	0.67 ± 0.07	0.1 ± 0.2	Cooperative
TM6	M249A	C/Interface	0.01	0.3 ± 0.1	0.97 ± 0.11	0.4 ± 0.2	0.79 ± 0.09	-0.1 ± 0.2	Cooperative
	A253V	C/Interface	0	0.0 ± 0.1	0.03 ± 0.01	0.3 ± 0.2	0.03 ± 0.00	-0.3 ± 0.2	Cooperative
	H254A	C	0	0.7 ± 0.1	0.05 ± 0.01	-0.2 ± 0.2	0.01 ± 0.00	0.9 ± 0.2	Moderate/Over
	G261A	C	0	4.9 ± 0.1	0.05 ± 0.01	4.0 ± 0.1	-0.02 ± 0.01	1.0 ± 0.2	Moderate/Over
	A265V	C	0	1.6 ± 0.3	0.37 ± 0.04	1.4 ± 0.2	0.29 ± 0.03	0.2 ± 0.4	Cooperative
	D268A	C/Interface	0.15	1.8 ± 0.2	0.54 ± 0.06	1.5 ± 0.1	0.48 ± 0.05	0.3 ± 0.2	Cooperative

Table S2. Mutation-induced changes in thermodynamic stability ($\Delta\Delta G^o_{WT-Mut}$) and activities of GlpG mutants relative to WT in DMPC:CHAPS bicelles. f_{ASA}: the fraction of buried residue area. In the “Location” column, “N” and “C” denote N- and C-subdomains, respectively. In the “Cooperativity profile” column, “Moderate/N”, “Moderate/C”, and “Moderate/Over” indicate moderately localized in N and C subdomains, and moderately overpropagated, respectively. Errors denote means ± SD from fitting. $\Delta\Delta G^o_{WT-Mut}$ and $\Delta\Delta\Delta G^o$ values are in kcal/mol.

Secondary structure	Mutation	Location	f_{ASA}	N-subdomain (95_N172_M)		C-subdomain (172_M267_C)		$\Delta\Delta\Delta G^o$	Cooperativity profile
				$\Delta\Delta G^o_{WT\text{-}Mut}$	Rel.Activity	$\Delta\Delta G^o_{WT\text{-}Mut}$	Rel.Activity		
TM1	M100A*	N	0.14	3.0 ± 0.3	0.55 ± 0.06	2.2 ± 0.3	0.64 ± 0.05	0.5 ± 0.4	Cooperative
	C104A*	N	0	1.2 ± 0.3	0.69 ± 0.04	0.9 ± 0.1	0.70 ± 0.05	0.3 ± 0.3	Cooperative
L1	V119A	N	0.05	2.0 ± 0.2	0.57 ± 0.06	0.9 ± 0.1	0.76 ± 0.05	1.1 ± 0.2	Moderate/N
	L123A	N	0.23	2.1 ± 0.2	0.55 ± 0.06	0.9 ± 0.1	0.68 ± 0.05	1.2 ± 0.2	Local/N
	W125A	N	0.09	2.8 ± 0.3	0.04 ± 0.10	1.7 ± 0.2	0.00 ± 0.01	1.1 ± 0.3	Moderate/N
	K132A	N	0.56	0.2 ± 0.3	0.71 ± 0.06	0.4 ± 0.1	0.52 ± 0.05	-0.2 ± 0.3	Cooperative
	F133A	N	0.80	1.3 ± 0.3	0.84 ± 0.06	0.5 ± 0.2	0.87 ± 0.05	0.8 ± 0.3	Cooperative
	F135A	N	0.73	0.4 ± 0.2	0.93 ± 0.06	0.2 ± 0.1	0.69 ± 0.05	0.2 ± 0.3	Cooperative
	W136A	N	0.46	2.7 ± 0.2	0.00 ± 0.02	1.7 ± 0.1	0.00 ± 0.03	1.0 ± 0.2	Moderate/N
	R137A	N	0.04	4.1 ± 0.2	0.01 ± 0.01	2.8 ± 0.1	0.01 ± 0.01	1.3 ± 0.2	Local/N
	Y138F*	N	0.25	1.8 ± 0.2	0.95 ± 0.06	0.6 ± 0.1	0.93 ± 0.05	1.2 ± 0.2	Local/N
	F139A	N	0.38	2.0 ± 0.2	0.47 ± 0.06	1.0 ± 0.1	0.47 ± 0.05	1.0 ± 0.2	Moderate/N
	T140A*	N	0.20	1.6 ± 0.2	0.85 ± 0.06	0.7 ± 0.1	0.60. ± 0.03	0.9 ± 0.2	Moderate/N
	L143A*	N	0.25	2.3 ± 0.2	0.76 ± 0.06	1.4 ± 0.1	0.65 ± 0.05	0.9 ± 0.2	Moderate/N
TM2	H150A	N	0.01	0.0 ± 0.3	0.05 ± 0.08	0.3 ± 0.2	0.02 ± 0.13	-0.3 ± 0.3	Cooperative
	N154A*	N/Interface	0	1.2 ± 0.2	0.01 ± 0.04	1.2 ± 0.3	0.01 ± 0.02	0.0 ± 0.4	Cooperative
	L155A	N	0.15	2.2 ± 0.2	0.75 ± 0.05	1.6 ± 0.2	0.60 ± 0.03	0.6 ± 0.3	Cooperative
	W158F*	N/Interface	0	1.0 ± 0.2	0.92 ± 0.06	0.1 ± 0.1	0.85 ± 0.05	0.9 ± 0.2	Moderate/N
	L161A*	N/Interface	0	2.0 ± 0.3	0.16 ± 0.06	2.7 ± 0.3	0.10 ± 0.06	-0.7 ± 0.4	Moderate/C
TM3	L174A*	N/Interface	0	3.7 ± 0.2	0.35 ± 0.06	3.3 ± 0.1	0.07 ± 0.07	0.4 ± 0.2	Cooperative
	T178A*	N	0.11	0.6 ± 0.2	0.77 ± 0.06	0.3 ± 0.1	0.66 ± 0.07	0.3 ± 0.2	Cooperative
	S181A*	N/Interface	0	-0.6 ± 0.2	1.03 ± 0.06	0.6 ± 0.1	1.00 ± 0.05	-1.2 ± 0.2	Moderate/Over
	F197A	N	0.01	1.7 ± 0.2	0.01 ± 0.03	0.6 ± 0.1	0.00 ± 0.07	1.1 ± 0.2	Moderate/N
TM4	S201T*	C/Interface	0	0.4 ± 0.2	0.02 ± 0.01	0.8 ± 0.2	0.00 ± 0.03	-0.4 ± 0.3	Cooperative
	A206G	C	0	0.4 ± 0.2	0.09 ± 0.09	0.6 ± 0.1	0.09 ± 0.06	-0.2 ± 0.2	Cooperative
	L207A*	C/Interface	0	4.1 ± 0.3	0.12 ± 0.01	2.7 ± 0.1	0.16 ± 0.02	1.4 ± 0.3	Local/N
	Y210F*	C	0	1.9 ± 0.2	0.50 ± 0.07	1.2 ± 0.1	0.66 ± 0.05	0.8 ± 0.2	Moderate/N
	R214A	C/Interface	0.10	0.9 ± 0.2	0.41 ± 0.06	0.6 ± 0.1	0.43 ± 0.05	0.3 ± 0.3	Cooperative
TM5	I223A	C/Interface	0.00	1.0 ± 0.3	0.24 ± 0.06	0.5 ± 0.1	0.23 ± 0.11	0.5 ± 0.3	Cooperative
	L225A*	C/Interface	0.03	-0.7 ± 0.2	0.27 ± 0.07	1.0 ± 0.1	0.10 ± 0.06	-1.6 ± 0.2	Local/C
	Q226A*	C	0.57	0.2 ± 0.2	0.82 ± 0.06	0.8 ± 0.2	0.51 ± 0.05	-0.6 ± 0.3	Moderate/C
TM6	M249A	C/Interface	0.01	0.3 ± 0.2	0.59 ± 0.06	0.5 ± 0.2	0.85 ± 0.05	-0.2 ± 0.3	Cooperative
	A253V*	C/Interface	0	1.5 ± 0.2	0.06 ± 0.01	0.9 ± 0.1	0.00 ± 0.06	0.6 ± 0.3	Moderate/Over
	H254A	C	0	1.5 ± 0.2	0.05 ± 0.01	-0.3 ± 0.1	0.01 ± 0.05	1.8 ± 0.3	Over
	G261A*	C	0	4.0 ± 0.2	0.05 ± 0.01	2.7 ± 0.1	-0.01 ± 0.06	1.3 ± 0.2	Over
	A265V*	C	0	2.3 ± 0.2	0.30 ± 0.06	1.3 ± 0.1	0.13 ± 0.05	1.0 ± 0.2	Moderate/Over
	D268A*	C/Interface	0.15	2.4 ± 0.2	0.44 ± 0.07	1.3 ± 0.1	0.28 ± 0.05	1.1 ± 0.2	Moderate/Over

Table S3. Mutation-induced changes in thermodynamic stability ($\Delta\Delta G^o_{WT\text{-}Mut}$) and activities of GlpG mutants relative to WT in DDM micelles. f_{ASA}: the fraction of buried residue area. In the "Location" column, "N" and "C" denote N- and C-subdomains, respectively. In the "Cooperativity profile" column, "Moderate/N", "Moderate/C", and "Moderate/Over" indicate moderately localized in N and C subdomains, and moderately overpropagated, respectively. The stabilities of the mutants marked with asterisks have previously been reported (*60*). The reproducibility of the previously reported data was confirmed in this study. Errors denote means ± SD from fitting. $\Delta\Delta G^o_{WT\text{-}Mut}$ and $\Delta\Delta\Delta G^o$ values are in kcal/mol.

A

Whole	A_1 (%)	$\tau_{R,1}$ (ns)	A_2 (%)	$\tau_{R,2}$ (ns)	A_3 (%)	$\tau_{R,3}$ (ns)	A_∞ (%)	$<\tau_R>$ (ns)	Adj-R^2	$\tau_{R,1/e}$ (ns)
Lip–Lip	14.2 ± 0.4	1.3 ± 0.1	39.8 ± 0.5	21 ± 1	46.0 ± 0.5	82 ± 1	0.0 ± 0.0	46 ± 1	0.999	36 ± 1
Prot–Lip	29.4 ± 0.6	19 ± 1	54.3 ± 0.5	112 ± 2	11.1 ± 0.6	527 ± 30	1.3 ± 0.1	132 ± 7	0.999	83 ± 1
$\Delta G^o_{Solv,Lip}$ = –0.62 ± 0.03 kcal/mol from $<\tau_R>$						$\Delta G^o_{Solv,Lip}$ = –0.50 ± 0.02 kcal/mol from $\tau_{R,1/e}$				
Det–Det120	30.5 ± 0.9	4.1 ± 0.2	50.3 ± 1.3	28 ± 1	18.1 ± 1.8	85 ± 4	0.0 ± 0.0	31 ± 2	0.998	22 ± 1
Prot–Det120	23.8 ± 0.4	4.1 ± 0.1	35.8 ± 0.3	58 ± 1	39.7 ± 0.3	302 ± 2	0.4 ± 0.0	143 ± 2	0.999	92 ± 1
$\Delta G^o_{Solv,Det120}$ = –0.90 ± 0.05 kcal/mol from $<\tau_R>$						$\Delta G^o_{Solv,Det120}$ = –0.85 ± 0.03 kcal/mol from $\tau_{R,1/e}$				
Det–Det150	16.6 ± 1.5	3.2 ± 0.4	39.4 ± 1.2	18 ± 1	43.7 ± 1.5	66 ± 1	0.0 ± 0.0	37 ± 2	0.998	29 ± 1
Prot–Det150	25.8 ± 0.3	6.2 ± 0.1	48.6 ± 0.4	83 ± 1	24.1 ± 0.5	307 ± 4	0.1 ± 0.0	118 ± 3	0.999	81 ± 1
$\Delta G^o_{Solv,Det150}$ = –0.69 ± 0.03 kcal/mol from $<\tau_R>$						$\Delta G^o_{Solv,Det150}$ = –0.61 ± 0.02 kcal/mol from $\tau_{R,1/e}$				

B

Headgroup	A_1 (%)	$\tau_{R,1}$ (ns)	A_2 (%)	$\tau_{R,2}$ (ns)	A_3 (%)	$\tau_{R,3}$ (ns)	A_∞ (%)	$<\tau_R>$ (ns)	Adj-R^2	$\tau_{R,1/e}$ (ns)
Lip–Lip	37.0 ± 0.5	2.2 ± 0.1	31.9 ± 2.4	27 ± 1	31.1 ± 2.6	64 ± 2	0.0 ± 0.0	29 ± 3	0.999	22 ± 1
Prot–Lip	33.1 ± 0.4	8.9 ± 0.2	51.8 ± 0.3	104 ± 1	9.8 ± 0.3	725 ± 39	0.5 ± 0.1	135 ± 6	0.998	64 ± 1
$\Delta G^o_{SolvEx,LipHead}$ = –0.91 ± 0.06 kcal/mol from $<\tau_R>$					$\Delta G^o_{SolvEx,LipHead}$ = –0.63 ± 0.03 kcal/mol from $\tau_{R,1/e}$					
Det–Det120	15.0 ± 2.7	1.5 ± 0.3	45.0 ± 2.4	6.1 ± 0.3	40.1 ± 0.7	34 ± 1	0.0 ± 0.0	17 ± 1	0.999	11 ± 1
Prot–Det120	31.3 ± 0.8	3.3 ± 0.1	38.7 ± 0.7	28 ± 1	28.6 ± 0.9	110 ± 2	1.5 ± 0.0	44 ± 2	0.998	27 ± 1
$\Delta G^o_{SolvEx,Det120Head}$ = –0.58 ± 0.03 kcal/mol from $<\tau_R>$					$\Delta G^o_{SolvEx,Det120Head}$ = –0.53 ± 0.06 kcal/mol from $\tau_{R,1/e}$					
Det–Det150	13.3 ± 1.1	0.1 ± 0.3	44.5 ± 1.1	6.4 ± 0.2	41.9 ± 0.6	38 ± 1	0.0 ± 0.0	19 ± 1	0.998	12 ± 1
Prot–Det150	21.0 ± 0.7	0.9 ± 0.1	40.5 ± 0.5	18 ± 1	38.2 ± 0.5	106 ± 1	0.3 ± 0.0	48 ± 1	0.999	30 ± 1
$\Delta G^o_{SolvEx,Det150Head}$ = –0.56 ± 0.02 kcal/mol from $<\tau_R>$					$\Delta G^o_{SolvEx,Det150Head}$ = –0.54 ± 0.02 kcal/mol from $\tau_{R,1/e}$					

C

Tail	A_1 (%)	$\tau_{R,1}$ (ns)	A_2 (%)	$\tau_{R,2}$ (ns)	A_3 (%)	$\tau_{R,3}$ (ns)	A_∞ (%)	$<\tau_R>$ (ns)	Adj-R^2	$\tau_{R,1/e}$ (ns)
Lip–Lip	25.2 ± 0.5	1.4 ± 0.1	30.8 ± 0.5	19 ± 1	44.1 ± 0.5	81 ± 1	0.0 ± 0.0	42 ± 1	0.999	30 ± 1
Prot–Lip	33.8 ± 0.4	5.5 ± 0.1	44.3 ± 0.5	79 ± 1	17.2 ± 0.5	323 ± 8	1.6 ± 0.0	97 ± 3	0.999	58 ± 1
$\Delta G^o_{SolvEx,LipTail}$ = –0.50 ± 0.02 kcal/mol from $<\tau_R>$					$\Delta G^o_{SolvEx,LipTail}$ = –0.39 ± 0.02 kcal/mol from $\tau_{R,1/e}$					
Det–Det120	48.5 ± 0.7	1.6 ± 0.1	29.4 ± 0.8	17 ± 1	21.9 ± 1.0	67 ± 2	0.0 ± 0.0	21 ± 1	0.997	9 ± 1
Prot–Det120	32.1 ± 0.4	3.6 ± 0.1	29.8 ± 0.3	61 ± 1	36.5 ± 0.3	388 ± 3	0.5 ± 0.0	164 ± 2	0.999	87 ± 1
$\Delta G^o_{SolvEx,Det120Tail}$ = –1.22 ± 0.03 kcal/mol from $<\tau_R>$					$\Delta G^o_{SolvEx,Det120Tail}$ = –1.34 ± 0.07 kcal/mol from $\tau_{R,1/e}$					
Det–Det150	36.2 ± 1.7	0.9 ± 0.1	28.7 ± 1.7	8.8 ± 1.0	34.6 ± 1.2	55 ± 2	0.5 ± 0.1	22 ± 1	0.998	11 ± 1
Prot–Det150	37.0 ± 0.3	4.6 ± 0.1	36.6 ± 0.3	76 ± 1	23.9 ± 0.4	314 ± 3	0.2 ± 0.0	107 ± 2	0.999	58 ± 1
$\Delta G^o_{SolvEx,Det150Tail}$ = –0.94 ± 0.04 kcal/mol from $<\tau_R>$					$\Delta G^o_{SolvEx,Det150Tail}$ = –0.98 ± 0.05 kcal/mol from $\tau_{R,1/e}$					

Table S4. Fitted parameters of time-dependent contact autocorrelation data with a triple exponential decay function for the whole (A), headgroup (B), and tail (C) regions of lipid (Lip) or detergent (Det) molecules on GlpG (Prot) and on themselves. *A*: % amplitude; τ_R: residence time; $<\tau_R>$: the amplitude-weighted average residence time; Adj-R^2: the adjusted *R*-square; ΔG^o_{Solv}: the solvation free energy of amphiphile molecules on the protein; $\tau_{R,1/e}$: the resident time at which the amplitude of contact autocorrelation decays to 1/*e* of the initial value. Errors denote means ± SD from fitting.

	$\Delta G^{o}_{N\text{-}I,l,w}$ (kcal/mol)	$m_{N\text{-}I}$ (kcal/mol·M^{-1})	$\Delta G^{o}_{I\text{-}U,l,w}$ (kca/mol)	$m_{I\text{-}U}$ (kcal/mol·M^{-1})	$\Delta G^{o}_{N\text{-}U,l,w}$ (kcal/mol)
DC12PC	5.4 ± 0.5	2.0	26.4 ± 0.1	7.2	31.8 ± 0.6
DC11PC	9.3 ± 4.8	4.7 ± 2.6	16.4 ± 1.4	4.2 ± 0.4	25.7 ± 6.2

Table S5. Equilibrium folding data of OmpLA. The fitted stabilities (ΔG^{o}) in the absence of GdnHCl and *m*-values (the dependence of ΔG^{o} on GdnHCl concentration) were obtained from a three-state model. For the titrations with DC12PC, we used fixed *m* values previously determined by global fitting on WT and many variants ($m_{N\text{-}I}$ = 2.0 kcal·mol^{-1}·M^{-1} and $m_{I\text{-}U}$ = 7.2 kcal·mol^{-1}·M^{-1}). For the titrations with DC11PC, we floated both *m*-values during fitting.

Data S1. Source data and statistical analysis results. Excel spreadsheets contain the numerical data used to construct all plots in the Main Text and Supplementary Materials. This Excel file also contain the statistical analysis results using Chow's test (Figs. 3A and 3B) and student's *t*-test (fig. S3B and fig. S5C).
